# RAB14 promotes epithelial-mesenchymal transition in bladder cancer through autophagy‑dependent AKT signaling pathway

**DOI:** 10.1038/s41420-023-01579-8

**Published:** 2023-08-09

**Authors:** Huanhuan Deng, Leihong Deng, Haichao Chao, Zhaojun Yu, Jianbiao Huang, Zhen Song, Lifen Peng, Tao Zeng

**Affiliations:** 1 Medical College of Nanchang University, Nanchang, Jiangxi 330006 China; 2 Department of Ultrasound Medicine, The First Affiliated Hospital of Nanchang University, Nanchang, Jiangxi 330006 China; 3 Department of Urology, The Second Affiliated Hospital of Nanchang University, Nanchang, Jiangxi 330006 China; 4 Department of Clinical trial center, Jiangxi Provincial People’s Hospital, No. 152, Aiguo Road, Nanchang, Jiangxi 330006 China

**Keywords:** Oncogenesis, Bladder cancer

## Abstract

Bladder cancer (BLCA) is the 9th most common cancer of mortality. Autophagy and epithelial to mesenchymal transition (EMT) have an essential role in cancer invasion and metastasis. However, the relationship between autophagy and EMT is still poorly understood in BLCA. Functional enrichment and pathway network analysis were carried out. Comprehensive protein-protein interactions (PPI) networks were proposed to prioritize candidate autophagy-related genes. Furthermore, an autophagy-related signature and a nomogram model were established by integrating clinical information and this signature risk score to evaluate candidate autophagy-related genes. RAB14 expression and its association with pathological information and survival were evaluated in samples from TCGA dataset. Knocking down RAB14 in T24 cells was constructed, and immunofluorescence staining, transmission electron microscopy, immunohistochemistry and western blotting and a series of functional assays were performed to evaluate the migration, invasion, EMT and autophagy abilities of BLCA cells. The autophagy-related gene RAB14 was the only candidate gene identified by three kinds of analytic approaches. RAB14 was highly upregulated in BLCA and correlated with clinical outcomes based on TCGA BLCA datasets. Knocking down RAB14 was found to inhibit EMT and autophagy in T24 cells. RAB14 levels were positively related to those of LC3B and Beclin1, two genes with critical roles in the autophagy process, and the correlation was further confirmed in clinical tissue specimens by IHC and western blot analysis. In addition, RAB14-promoted EMT, migration, and invasion in T24 cells could be partially reversed by autophagy activator, rapamycin. The effects of RAB14 on autophagy was associated with level of p-Akt, indicating that they were possibly mediated via PI3K/AKT signaling. These findings indicated that autophagy-related gene RAB14-promoted EMT, migration and invasion of bladder cancer via the Akt-associated autophagic pathway.

## Introduction

Bladder cancer (BLCA) is the 9^th^ most common cause of mortality, which is estimated to affect approximately 500,000 patients with BLCA are newly diagnosed each year [1, 2]. Although many efforts have been made to develop new treatments and diagnosis, 5-year survival rate of BLCA has been less than 30% [3, 4]. Thus, there is an imperative need for discovering an ideal novel target of therapeutic intervention in bladder cancer.

Epithelial–mesenchymal transition (EMT) has an essential role in cancer invasion and metastasis [5]. During EMT, epithelial cells lose their epithelial properties via acquiring migratory and invasive mesenchymal characteristics [6]. Moreover, epithelial cells will undergo skeletal structure changes (e.g., E-cadherin and Vimentin) [7]. The decrease in E-cadherin and Vimentin gain are two crucial markers of EMT in tumor cells [8], which is a potential mechanism for the invasion-metastasis cascade of tumor cells [9]. Substantial evidence indicates that autophagy is closely related to the process of EMT by regulating metabolic stress and other microenvironmental change in various cancers [10]. However, the role between EMT and autophagy in bladder cancer remains to be investigated.

Autophagy is an evolutionary conserved mechanism for degradation of misfolded proteins and damaged organelles, and its dysfunction has been contributed to several pathological conditions including cancer metastasis [11]. Autophagy has “double-edged sword” effects in cancer cells, either promoting tumor growth under metabolic stress or suppressing tumor progression via protecting the genomic integrity [12, 13]. Therefore, based on the role of autophagy in cancer, identifying the role of autophagy in tumor progression and metastasis would be beneficial to target tumors. Current evidence has indicated that autophagy could maintain cells survival, but an unrestrained autophagy may lead to cell death [14]. However, the exact effects of autophagy on the EMT in BLCA remain unknown.

In this study, the autophagy-related gene RAB14 was the only candidate gene identified by three kinds of analytic approaches. RAB14 was highly upregulated in BLCA and correlated with clinical outcomes based on TCGA BLCA datasets. Based on the hypothesis that autophagy-related gene RAB14 abnormal expression may mediate a crosstalk of the autophagy and EMT during bladder cancer metastasis, we focused on investigating effects of RAB14 by AKT pathway, as well as on EMT phenotype of bladder cancer by inducing autophagy via Akt/mTOR axis. Our results may provide a novel diagnostic biomarker and potential therapeutic target for BLCA.

## Results

### Identification and screening of DEGs

**Fig. 1:**
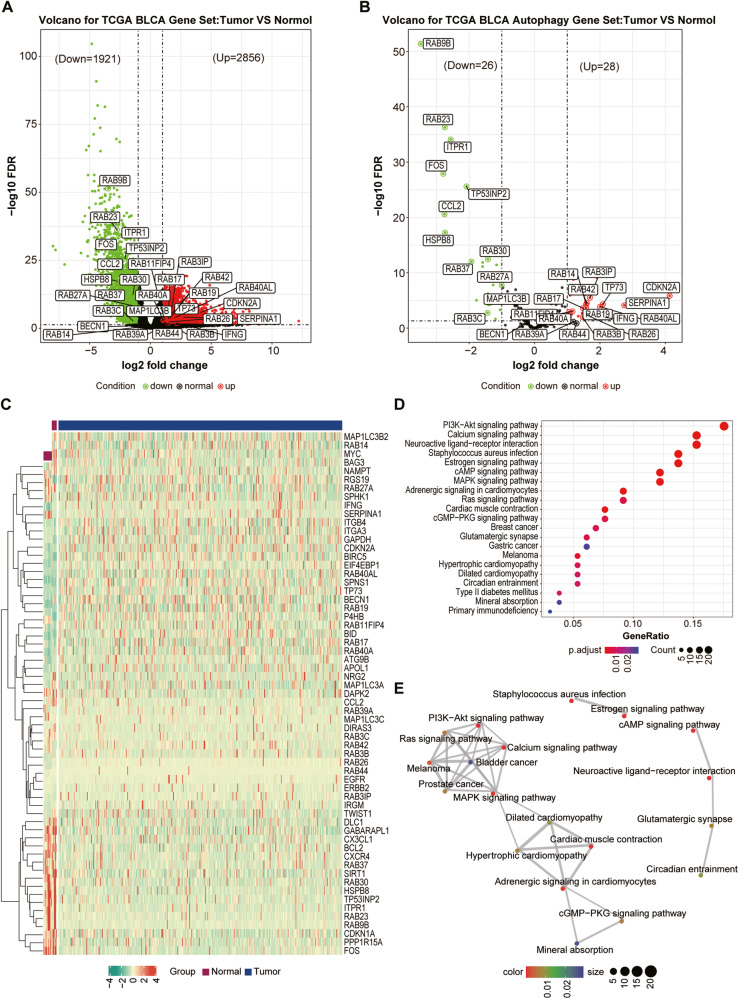
Identification and screening of Differentially Expressed Genes in BCLA. **A** Volcano plot of DEGs between BCLA patients and individuals. The red and green points represent up- and down-regulated genes, respectively. **B** Volcano plot of autophagy-related DEGs between BCLA patients and individuals. The red and green points represent up- and down-regulated genes, respectively. **C** Heatmap of 54 autophagy-related DEGs. **D** KEGG enrichment analysis of 54 autophagy-related DEGs. **E** The constructed pathway network plots of 54 autophagy-related DEGs.

To improve our understanding of the molecular basis in BLCA patients, first we used gene expression profiles from BLCA patients in TCGA database. The results showed that 2856 upregulated and 1921 down-regulated DEGs were obtained by comparing BLCA tissues versus normal bladder tissues (Fig. 1A, |log_2_ FC | ≥ 1, FDR < 0.05). The autophagy-related genes were downloaded from Human Autophagy Database (HADb) and 28 upregulated and 26 down-regulated of autophagy-related genes were shown in volcano plots (|log_2_ FC | ≥ 1, FDR < 0.05) and the heatmap (Fig. 1B, C and Table S1).

Functional enrichment analyses were then carried out based on these up- and down-regulated autophagy-related genes. KEGG enrichment results shown that these genes were involved in multiple significant pathways, including PI3K-Akt signaling pathway, calcium-signaling pathway, neuroactive ligand-receptor interaction, Ras signaling pathway and cAMP signaling pathway (Fig. 1D and Table S1). Furthermore, the constructed pathway network plots were analyzed by KEGG and four significant pathways closely related to BLCA were identified, including PI3K-Akt signaling pathway, calcium-signaling pathway, Ras signaling pathway and MAPK signaling pathway (Fig. 1E and Table S1), suggesting these pathway may have important roles in BLCA.

### Identification of the candidate causative gene RAB14

**Fig. 2:**
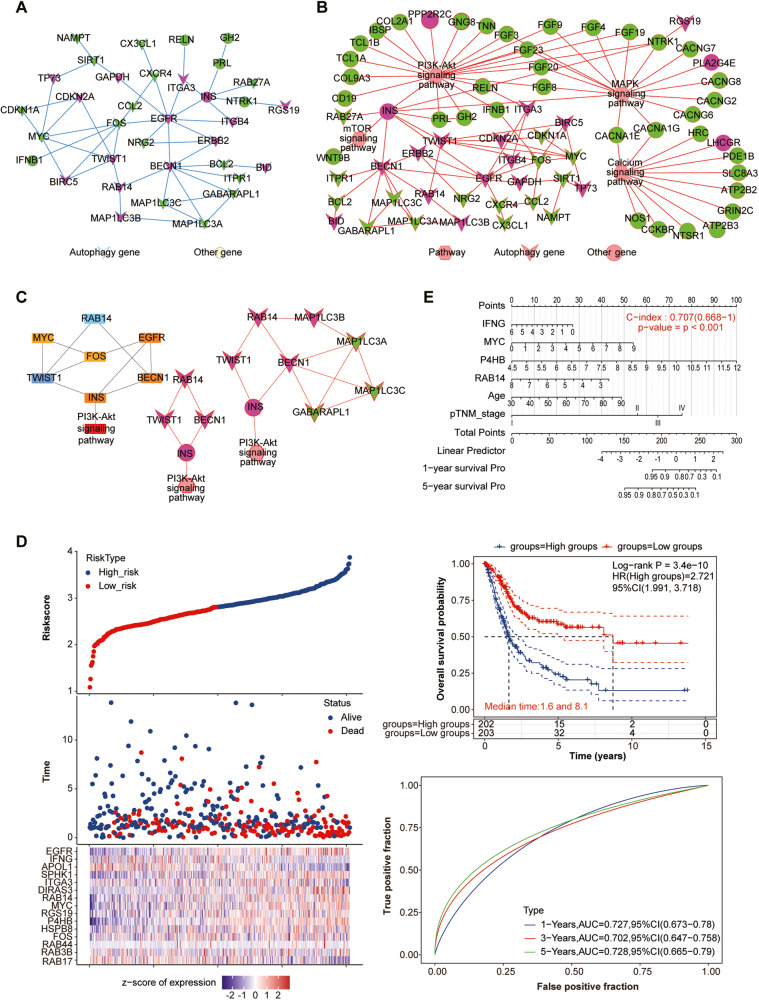
Identification of the candidate causative gene RAB14. **A** PPI networks of 54 autophagy-related genes. **B** PPI networks of 54 autophagy-related genes and 4777 DEGs. **C** Key subnetworks and hub genes of 54 autophagy-related genes. **D** LASSO Cox regression analysis of 54 autophagy-related genes. **E** A nomogram model for OS prediction of 54 autophagy-related DEGs.

Comprehensive PPI networks were proposed to prioritize 54 candidate autophagy-related genes in BLCA. 14 upregulated and 15 down-regulated genes were identified (Fig. 2A and Table S2). We combined these 54 genes with 4777 DEGs in BLCA to construct PPI networks for further screening (Fig. 2B and Table S2). Further, the key subnetworks and hub genes were determined by the Cytoscape plugin “MCODE” and “CytoHubba” on the PPI network (Fig. 2C and Table S2). Sub-networks with the high score were selected in the PPI networks of 54 above genes and the hub genes were identified, including RAB14, TWIST1, BECN1, FOS, MYC, INS and EGFR (Fig. 2C). For the PPI networks of all DEGs, the hub genes were identified, including RAB14, TWIST1, BECN1, MAP1LC3B, MAP1LC3A, MAP1LC3C, INS and GABARAPL1 (Fig. 2C). Considering the genes identified by two PPI networks, RAB14, TWIST1, BECN1 and INS were identified (Fig. 2C and Table S2).

Furthermore, LASSO Cox regression analysis were proposed to prioritize 54 candidate autophagy-related genes and a fourteen autophagy-related-gene signature was identified (Fig. 2D and Table S2), whose risk score of each patient was generated using the following risk score formula: Risk score = (0.0711) × RAB14 + (0.0121) × FOS + (0.0185) × HSPB8 + (0.2439) × P4HB + (0.0376) × RGS19 + (0.0407) × MYC + (0.1031) × DIRAS3 + (-0.0324) × ITGA3 + (0.0242) × SPHK1 + (-0.0854) × APOL1 + (-0.0937) × IFNG+ (0.0557) × EGFR + (-0.0651) × RAB17 + (-0.0036) × RAB3B + (-0.0052) × RAB44. Kaplan–Meier (KM) curves of these fourteen genes were shown in Fig. 2E. Finally, a nomogram for OS prediction was constructed using the six prognostic factors and a four autophagy-related genes were identified, including RAB14, IFNG, MYC and P4HB (Fig. 2F and Table S2).

Interestingly, the autophagy-related gene RAB14 is the only candidate gene identified in combination of three different analytic approaches. Therefore, *RAB14* was selected as the target-of-interest in our following validation experiments.

### RAB14 was highly upregulated in BLCA and correlates with clinical outcomes

**Fig. 3:**
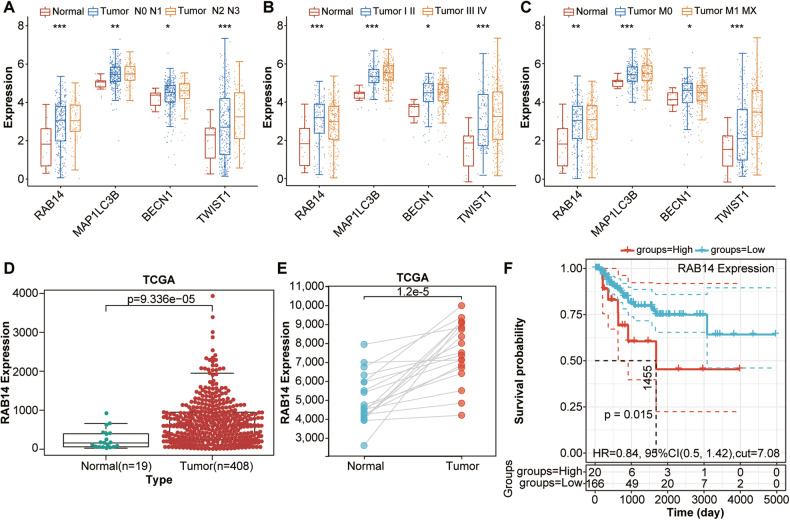
RAB14 was highly upregulated in BLCA and correlated with clinical outcomes. **A**–**C** The expression of RAB14-related genes, including RAB14, TWIST1, BECN1 and MAPILC3B, were analyzed between tumor and normal tissues. **D** The expression of RAB14 was significantly higher in BLCA than normal adjacent tissues. **E** The expression of RAB14 was significantly higher in BLCA compared to paired normal tissues. **F** Kaplan–Meier curves showed high RAB14 expression had worse prognosis of BLCA.

To explore the potential prognostic value of RAB14 in BLCA, the expression of RAB14-related genes (RAB14, TWIST1, BECN1 and MAPILC3B) were analyzed between tumor and normal tissues. The expression of RAB14 was significantly higher in BLCA than normal tissues, while the expression of TWIST1, BECN1 and MAPILC3B was significantly lower than normal tissues (Fig. 3A–D). In addition, the expression of RAB14 was significantly higher in BLCA compared to adjacent tissues and their paired normal tissues (Fig. 3E, F). Kaplan–Meier (KM) curves showed that Patients with higher expression levels of RAB14 have worse prognosis (Fig. 3G). Taken together, our results indicated that RAB14 might serve as an effective indicator for poor prognosis of BLCA.

### RAB14-promoted epithelial‑mesenchymal transition (EMT) in bladder cancer

**Fig. 4:**
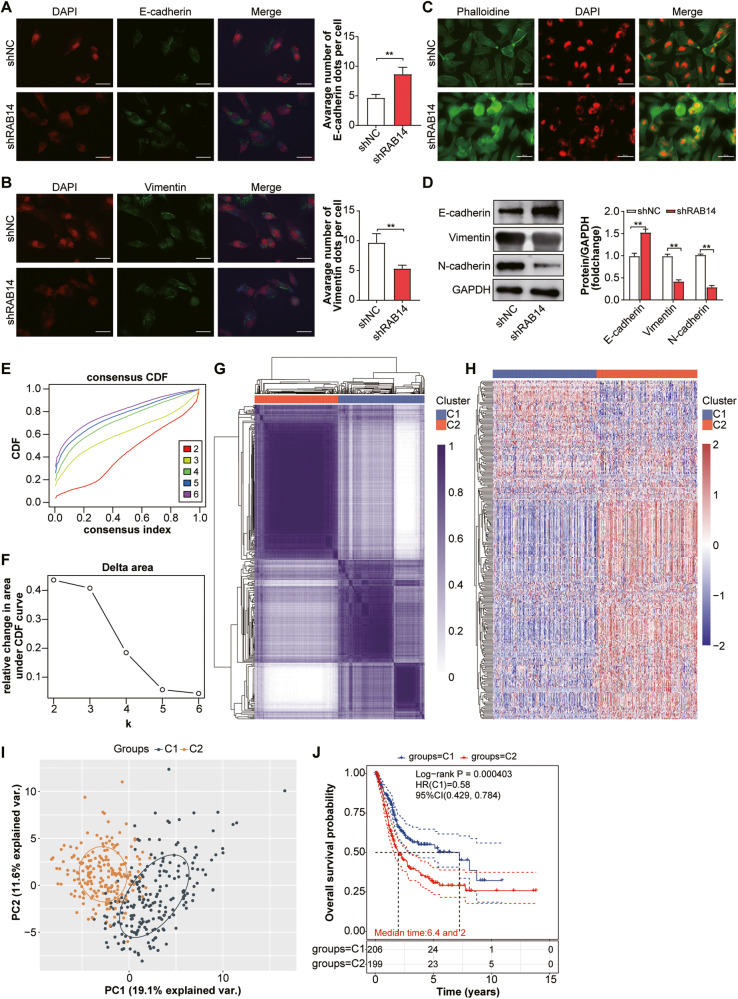
RAB14-promoted epithelial‑mesenchymal transition (EMT) in bladder cancer. **A** Immunofluorescence staining of E-cadherin in shRAB14 T24 cells and control. **B** Immunofluorescence staining of Vimentin in shRAB14 T24 cells and control. **C** Immunofluorescence analysis of cellular morphology in shRAB14 T24 cells and control. **D** Western blot of E-cadherin, N-cadherin and Vimentin. **E** Consensus clustering cumulative distribution function (CDF). **F** Relative change in the area under the CDF curve. **G** Consensus clustering matrix of two clusters. **H** Heatmap of two different clusters (C1, C2) based on RAB14-related EMT gene expression. **I** Principal components analysis (PCA) of two different clusters (C1, C2) from TCGA BLCA samples for *k* = 2. **J** Kaplan–Meier curve of overall survival of patients with BCLA in two clusters. *P < 0.05, **P < 0.001.

To identify whether cancer migration and invasion are involved in EMT, we explore the effect of RAB14 on EMT markers, including epithelial marker (E-cadherin) and mesenchymal marker (vimentin). Immunofluorescence staining revealed that E-cadherin was upregulated and Vimentin was significantly down-regulated in shRAB14 T24 cells compared to control (Fig. 4A, B). The shRAB14 T24 cells had a cobblestone-like appearance typical of epithelial cells and fibroblastic cellular morphology with F-actin fibers visible in immunofluorescence (IF) analysis, whereas shNC T24 cells had an exhibited an elongated, spindle-like of normal epithelial cells (Fig. 4C).

Next, we examined the expression pattern of classic EMT markers. Epithelial markers (E-cadherin) and mesenchymal markers (N-cadherin and Vimentin) were further detected by Western blot in T24 cells. Our data showed that E-cadherin was increased in the shRAB14 T24 cells, while N-cadherin and Vimentin were decreased (Fig. 4D and Supplementary Fig. S1A). These results indicated that RAB14 might promote EMT in BLCA.

To explore the potential prognostic value of RAB14-related genes in BLCA, RAB14-related genes were applied to classify the TCGA BCLA patients by a consensus clustering analysis. Based on the relationship between the expression of these genes and BCLA subtypes, BCLA patients were divided into two clusters (C1 and C2; Fig. 4E, F and Table S3). Interestingly, the Kaplan–Meier overall survival curves further showed that C1 had a longer OS time than C2 [hazards ratio (HR): 0.58, 95% confidence interval (CI): 0.429-0.784, *P* = 4.03e-4] in Fig. 4G. Taken together, our data indicated that RAB14 expression might be associated with clinical outcomes through promoting EMT in BLCA.

### RAB14 expression was correlated with autophagy-related genes

**Fig. 5:**
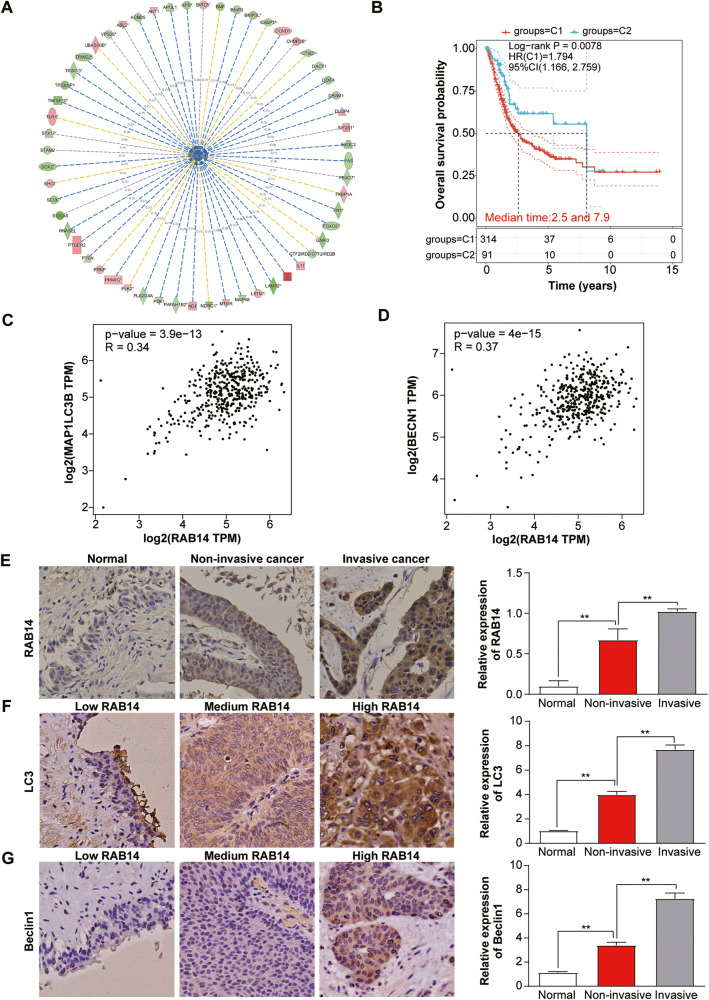
The expression of RAB14 was correlated with autophagy-related genes. **A** IPA data analysis showing downregulation of RAB14 and significantly suppressed autophagy. **B** Kaplan–Meier curve of RAB14-related autophagy genes predicted overall survival of BCLA patients from two clusters. **C** RAB14 was positively correlated with the autophagy-related gene LC3B from BLCA-related gene microarray data in the TCGA database. **D** RAB14 was positively correlated with the autophagy-related gene Beclin1 from BLCA-related gene microarray data in the TCGA database. **E** IHC staining of RAB14 and its expression levels in normal bladder epithelium, NMIBLCA and MIBLCA tissue specimens. **F** IHC staining of LC3B and its expression levels in the same sample set. **G** IHC staining of Beclin1 and its expression levels in the same sample set.

Autophagy is closely related to the process of EMT by regulating metabolic stress and other microenvironmental change in various cancers [10]. To explore the relationship between RAB14 and autophagy in BLCA, we first constructed a RAB14-silenced cellular model in T24 cells, and primary data regarding gene expression profiles was obtained by gene chip technology. Subsequently, we made pathway predictions through Gene Ontology (GO) enrichment analysis and gene function network mapping, and further integrated functional analysis via Ingenuity Pathway (IPA) and relevant literature. We found that these DEGs were mainly related to cell proliferation, autophagy, apoptosis, signal transduction, tumor invasion and metastasis. Among these systemic functions, autophagy was the one most significantly inhibited and its Z-score ranked first in all functions and diseases, strongly suggesting that RAB14 was closely related to autophagy in BLCA cells (Fig. 5A). Furthermore, a RAB14-related-autophagy gene signature was able to identify BLCA subgroups and predict prognosis of BLCA (Fig. 5B). RAB14 was positively correlated with the autophagy-related genes LC3B and Beclin1 from BLCA-related gene microarray data in the TCGA database (Fig. 5C, D). These data indicated that RAB14-related autophagy genes might be associated with clinical outcomes of BLCA.

To further investigate the expression of RAB14 and autophagy-associated proteins LC3B and Beclin1 in carcinoma and adjacent normal tissues in BLCA patients, IHC staining of 80 BLCA tumor tissue samples and paired BLCA tissue was performed, then the correlation analysis was used in the analysis of the correlation between RAB14 and autophagy genes (LC3B and Beclin1). BLCA tissue specimens for IHC staining were classified into muscle-invasive BLCA (MIBLCA), non-muscle-invasive BLCA (NMIBLCA), and adjacent normal bladder tissue samples with clinical features. IHC showed that the positive expression rates of RAB14, LC3 and Beclin1 proteins in BLCA were 76.25% (61/80), 65.0% (52/80) and 61.25% (49/80) respectively, while the positive expression rates in adjacent tissues were 23.75 (19/80), 35.0% (28/80) and 38.75% (31/80) (Fig. 5E–G). Furthermore, High RAB14, LC3 and Beclin1 expression was positively correlated with malignant features such as lymph node metastasis (*P* < 0.01), tumor staging (*P* < 0.05), tumor differentiation (*P* < 0.05). However, no difference was found for age, gender. Moreover, the correlation analysis displayed that RAB14 levels were positively associated with those of LC3B and Beclin1 in BLCA tissues. These results suggest that RAB14, LC3 and Beclin1 may promote BLCA progression.

### Knocking down RAB14 inhibited autophagy in BLCA cells

**Fig. 6:**
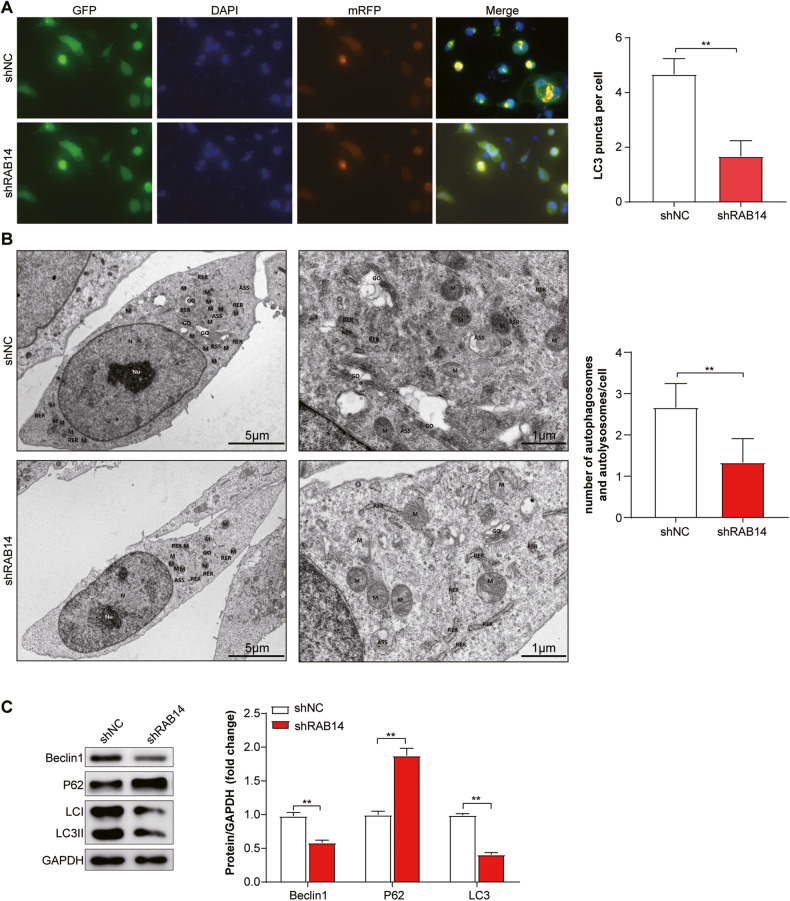
Knocking down RAB14 inhibited autophagy in BLCA cells. **A** Immunofluorescent staining showing the number of LC3 punctas in the T24-mRFP-GFP-LC3 KD cells and their control cells. Cells were stained by indirect immunofluorescence using anti-LC3 antibody, and the number of LC3 puncta per cell was quantified. Scale bars = 100 μm. **B** Representative TEM ( × 20,000) images of T24 cells and quantification of autophagic vacuoles (autophagosomes and autolysosomes) in subsets of 10 randomly-selected cells of each type. Red arrows, autophagic vacuoles. **C** Western blotting assay showed that the expression of LC3B and Beclin1 decreased and P62 showed the opposite change with the knocking down of RAB14. *P < 0.05, **P < 0.001.

We investigated the role of LHPP in autophagy in BLCA cells using short hairpin RNA (shRNA)–mediated knockdown. We knocked down RAB14 in T24 cells, which transfected by autophagy double-labeled adenovirus (mRFP-GFP-LC3). Screen stable cell line (T24-mRFP-GFP-LC3) was screened through labels of mRFP and GFP. The dynamic process of autophagosome-autolysosomes and autophagosomes formation were observed by lalser confocal microscope and electron microscope. Meanwhile, the autophagy flow and its strength were monitored and quantified by counting the number of different color spots. As shown in Fig. 6A, B, knockdown RAB14 decreased autolysosomes and autophagosomes in T24 cells compared with the control group under both electron and lalser confocal microscope. The number of LC3 punctas per cell was reduced by more than 2-fold (*P* < 0.05) due to knockdown RAB14, indicating that RAB14 may promote the formation of autophagy. Western blotting assay showed that the expression of LC3B and Beclin1 decreased and P62 showed the opposite change with the knocking down of RAB14 (Fig. 6C Supplementary Fig. S1B).

### The activation of autophagy reversed RAB14-promoted EMT, migration and invasion in bladder cancer cells

**Fig. 7:**
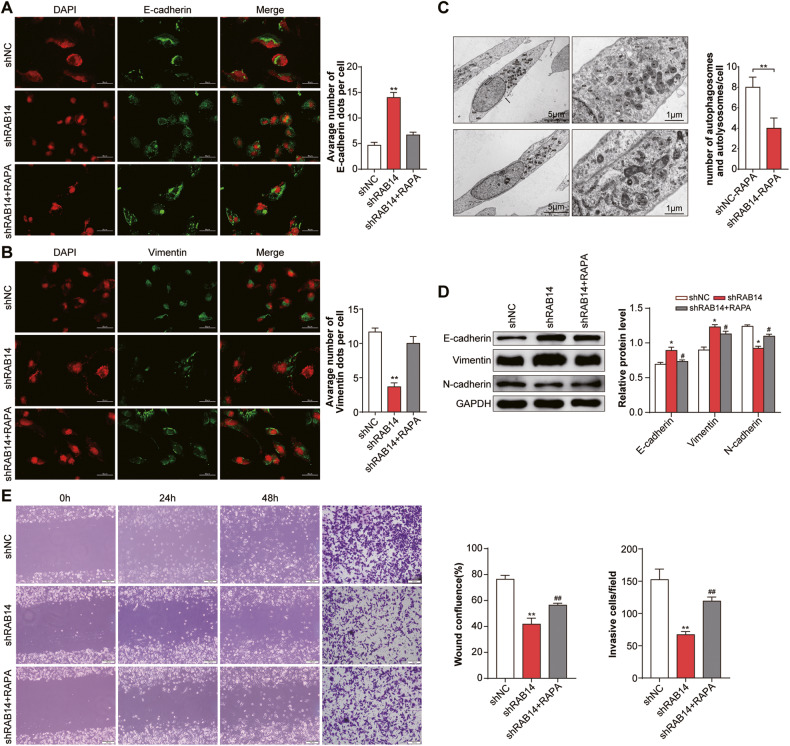
The activation of autophagy reversed RAB14-promoted EMT, migration and invasion in bladder cancer cells. **A** Immunofluorescence staining of E-cadherin. T24 cells were treated with rapamycin (RAPA), which is a specific inhibitor of mTOR protein, to study the effect of autophagy. Immunofluorescent staining showed that shRAB14 + RAPA group showed similar levels of E-cadherin as shNC group, but it showed E-cadherin was down-regulated compared with shRAB14 group. **B** Immunofluorescence staining of Vimentin. Immunofluorescent staining showed that shRAB14 + RAPA group showed similar levels of vimentin as shNC group, but vimentin was significantly upregulated compared with shRAB14 group. **C** Representative TEM ( × 20,000) images of T24 cells and quantification of autophagic vacuoles (autophagosomes and autolysosomes) in subsets of 10 randomly-selected cells of each type. **D** Western blotting assay of E-cadherin, N-cadherin and Vimentin. **E** Cell migration and invasion were ananlyzed. *P < 0.05, **P < 0.001.

As for knocking down RAB14 inhibited autophagy in BLCA cells, we further investigated whether activation of autophagy promoted EMT. T24 cells were treated with rapamycin (RAPA), which is a specific inhibitor of mTOR protein, to study the effect of autophagy. Immunofluorescent staining showed that shRAB14 + RAPA group showed similar levels of E-cadherin and vimentin as shNC group, but it showed E-cadherin was down-regulated and vimentin was significantly upregulated compared with shRAB14 group (*P* < 0.05) (Fig. 7A, B). As shown in Fig. 7C, shRAB1414 + RAPA group decreased autolysosomes and autophagosomes in T24 cells compared with the control group under both electron microscope (Fig. 7C). In T24 cells co-treated with shRAB14 and RAPA, western blotting assay further showed downregulation of E-cadherin expression and upregulation of N-cadherin and Vimentin expression compare with shRAB14 (Fig. 7D and Supplementary Fig. S1C).

In T24 cells co-treated with shRAB14 and RAPA, Enhanced cell migration and invasion were observed in shRAB14 group but not control cells, and shRAB1414-mediated anti-metastasis effects were reversed (Fig. 7E). This data indicated that RAB14-promoted EMT, migration, and invasion in T24 cells could be reversed by the activation of autophagy. Collectively, our results suggested that the promotion effects of RAB14 on the migration and invasion abilities of bladder cancer cells likely worked through the autophagy-EMT pathway.

### RAB14 might promote autophagy in BLCA via regulating the AKT signaling pathway

**Fig. 8:**
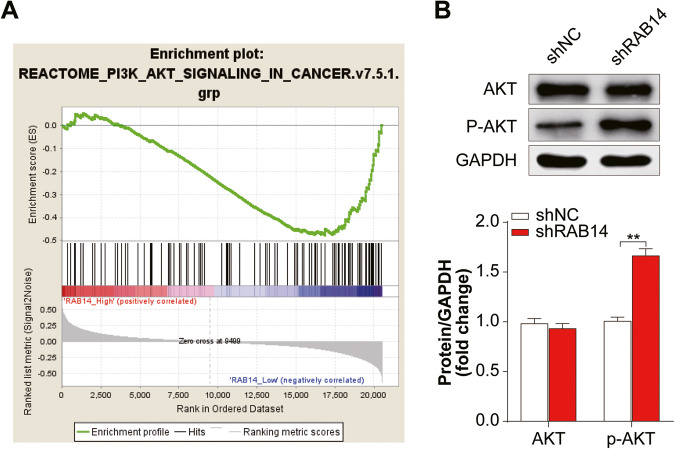
RAB14 might promote autophagy in BLCA via regulating the AKT signaling pathway. **A** Gene Set Enrichment Analysis showing a positive correlation between RAB14 expression and PI3K/AKT signaling pathway in BLCA. **B** Levels of AKT, p-AKT, LC3 proteins were assessed in T24 cells stably transfected with shRAB14. Data are shown as mean ± SD; ns no statistically significant difference, *P < 0.05, **P < 0.01.

The GSEA analysis results showed that RAB14 negatively regulated PI3K/AKT signaling pathways (NES = -1.45, *P* = 0.039; Fig. 8A). Furthermore, KEGG enrichment analysis of autophagy-related genes showed that PI3K-Akt signaling pathway might involved in BLCA (Fig. 1D, E). We analyzed the expression level of p-AKT in shRAB1414 group by western blot analysis. While the overall level of AKT remained unchanged, the amount of p-AKT in shRAB1414 group was significantly higher than the control group (*P* < 0.05; Fig. 8B and Supplementary Fig. S1D), suggesting that RAB14 may be able to inhibit phosphorylation of AKT.

## Discussion

Recent studies have shown that autophagy and EMT have an essential role in cancer invasion and metastasis [5, 11]. However, the relationship between autophagy and EMT is still poorly understood. Herein, we aimed to find a strong autophagy-related predictor of poor prognosis of BLCA with the help of high-throughput sequencing technology. Interestingly, the autophagy-related gene RAB14 was the only candidate gene identified by three different kinds of analytic approaches. RAB14 was highly upregulated in BLCA and correlated with clinical outcomes based on TCGA BLCA datasets. Therefore, RAB14 were finally determined as the target-of-interest in our following validation experiments.

RAB14, as an important member of RAS oncogene family, was the last member of the RAB11 subfamily and identified together with RAB1, RAB5 and RAB7 in the proteome of endosomes isolated from migrating cells [15]. RAB14 has been proved as an oncogene in various cancer types [16–19]. Overexpression of RAB14 led to the proliferation and invasion of non-small cell lung cancers by regulating Yap signaling pathway [17]. Knocking down RAB14 repressed the proliferation and migration of OSCC cells and enhanced their chemosensitivity [16]. These studies indicated that RAB14 might act as an oncogene in human carcinomas. RAB14 levels correlated with lymph node metastasis, poor differentiation, a high-grade tumor stage, as well as unfavorable prognostic outcomes for BLCA patients in our previous study [20].

EMT plays a crucial role in the regulation of invasion and metastasis [21]. In this study, we found that Knocking down RAB14 led to increased expression of E-cadherin and reduced N-cadherin, Vimentin expression in BLCA cells. Therefore, our analyses suggest that autophagy-related gene RAB14 positively regulates EMT in BLCA. It has been suggested that RAB7 regulate autophagy pathway to facilitate cancer cell invasions as well as metastasis [22], we supposed that RAB14 could promote cell migration and invasion by regulating autophagy. Specifically, our data demonstrated that knocking down RAB14 decreased expression of classical autophagy markers such as LC3 and Beclin1. Moreover, we observed decreased numbers of autophagosomes and autolysosomes in T24 cells transfected with shRAB14. To our knowledge, for the first time, we demonstrated that RAB14 regulated cancer cell autophagy.

To further investigate whether autophagy had an effect on BLCA cell migration and invasion, we respectively dealt with cells with rapamycin as an autophagy activator and then detect the migration capacity alteration of BLCA cells. We found that activation of autophagy in BLCA cells could reverse the observed defects in migration and invasion upon knocking down RAB14. As a result, the promotion effects of RAB14 on the migration and invasion of BLCA acted through the autophagy-EMT pathway.

The PI3K/AKT/mTOR signaling pathway could regulate apoptosis and autophagy in cancer cells [23]. AKT was involved in inactivation of pro-apoptotic factors, procaspase-9 and Bad via phosphorylation to regulate apoptosis. It also suppresses GSK3 to initiate cell cycle progression by regulating RB hyperphosphorylation as well as inactivation [24]. Moreover, AKT phosphorylates p21 and suppresses its anti-proliferative effects by retaining it in the cytoplasm. PIP3, a second messenger, is important for AKT translocation to plasma membranes where its phosphorylation and activation are mediated by PDK1 and PDK2 [25]. Moreover, autophagy-associated proteins, including ULK1, Beclin1, and ATG1 enhance autophagic initiation as well as autophagosome nucleation [13]. The mTOR suppresses autophagy via phosphorylation of the autophagy-associated proteins [26]. We established that that R knocking down RAB14 suppressed PI3K and p-AKT levels, promoting autophagy. Therefore, our data suggested that RAB14 suppression inhibits the Akt pathway, thereby inducing autophagy and inhibited the malignant progression of BLCA.

In conclusion, we established a novel mechanism of RAB14 in regulation of PI3K/Akt-associated autophagy and downstream process of cell migration as well as invasion. However, how autophagy regulates invasion and metastasis of bladder tumors remains to be further studied. Combined targeting of RAB14 and Akt pathway is a possible strategy for dysregulation of cancer metastasis, one of the major causes of poor prognostic outcomes among BLCA patients.

## Materials and methods

### Screening of differentially expressed genes and enrichment analysis

The RNA-sequencing data with the corresponding clinical information were downloaded from TCGA BLCA dataset (https://portal.gdc.cancer.gov/). The autophagy-related genes were downloaded from Human Autophagy Database (http://www.autophagy.lu/). Differentially expressed genes (DEGs) were screened using the Limma R package. DEGs between groups were selected using the criteria (|log_2_ FC | ≥ 1, FDR < 0.05). All the data were visualized by plotting volcano plots using the “ggplot2” R package. The heatmap was constructed by the “pheatmap” R package (version 1.0.12).

KEGG pathway enrichment analysis was performed using the cluster Profiler R package. The results were visualized using the “ggplot2” R package. The constructed pathway network plots were conducted using R cluster Profilter. Significantly enriched pathways featured *P* < 0.001.

### Identification of the candidate causative gene

Comprehensive protein-protein interactions (PPI) networks were proposed to prioritize candidate autophagy-related genes in BLCA using the string database (https://www.string-db.org/). Combined with all the DEGs in BLCA, candidate autophagy-related genes were further constructed the PPI networks. The key subnetworks and hub genes were determined by the Cytoscape plugin “MCODE” and “CytoHubba” on the PPI network using Cytoscape v3.8 (confidence > 0.9).

Using the R software package ConsensusClusterPlus (v1.54.0) for consistency analysis, the maximum number of clusters is 6, and 80% of the total sample is drawn 100 times, clusterAlg = “hc” innerLinkage = “ward.D2”. The R software package pheatmap (v1.0.12) was used for clustering the heatmaps. Kaplan–Meier (KM) curves of candidate autophagy-related genes were generated using R packages “survival”.

Finally, a predictive nomogram model was constructed based on the results of the multivariate Cox analysis using the “rms” R package. We conducted 1- and 5-year OS calibrations to determine the predictive accuracy of the nomogram model.

### Correlation analysis of RAB14 expression and clinicopathological characteristics

To explore the potential prognostic value of RAB14 in BLCA, the expression of RAB14-related genes (RAB14, TWIST1, BECN1 and MAPILC3B) were analyzed between tumor and normal tissues from TCGA BLCA database. In addition, the expression of RAB14 was analyzed in BLCA compared to adjacent tissues and their paired normal tissues from TCGA BLCA database. Kaplan–Meier (KM) curve of RAB14 was generated using R packages “survival”.

For the potential prognostic value of RAB14-related EMT genes, a predictive model was constructed. Using the R software package ConsensusClusterPlus (v1.54.0) for consistency analysis, the maximum number of clusters is 6, and 80% of the total sample is drawn 100 times, clusterAlg = “hc” innerLinkage = “ward.D2”. The R software package pheatmap (v1.0.12) was used for clustering the heatmaps. Kaplan–Meier (KM) curves of RAB14-related EMT genes were generated using R packages “survival”.

### Tissue specimens and cell lines

Eighty BLCA tissue samples in which 30 were paired with adjacent normals were acquired from patients that underwent radical cystectomy at the Second Affiliated Hospital of Nanchang University from January 2012 to December 2016. Prior to surgery, patients had not been administered with radio- or chemo- therapy. Written informed consents were acquired from all participants while the Ethical Committee and Institutional Review Board of The Second Affiliated Hospital of Nanchang University approved this study [approval No. Review (2011) No. (101)]. Histopathologic analyses of the tissue samples were assessed and verified by two pathologists, based on the World Health Organization and the Nevin staging system criteria.

### Cell cultivation and transfection

Human BLCA cell line T24 was procured from the China Academia Sinica Cell Repository (Shanghai, China). Culture of T24 cells was done in RPMI 1640 (Gibco, Grand Island, NY, USA) with 10% fetal calf serum (FCS; Gibco) at 37 °C in a 5% CO_2_ incubator. Then, cell transfections with plasmids expressing short-hairpin RNA of RAB14 (shRAB14; LV-RAB14-RNAi 36194-1, Genechem, Shanghai, China), plasmids expressing empty vector (LV-psc4504-1-Vector), or with GFP-RFP-LC3 were done using Lipofectamine 2000 (Invitrogen Preservation, Carlsbad, CA, USA) as instructed by the manufacturer to assess knockdown efficiency by western blot (WB) analysis. Subsequently, shRAB14 (LV-sh-36194-1) and its negative control (shNC) were packaged into lentiviral particles as instructed by the manufacturer, and transduced T24 cells, which were obtained for subsequent experiments.

### Affymetrix gene expression profile chip detection and bioinformatics analysis

Total RNA was extracted in triplicate from highly aggressive cells transfected with shRAB14 (RAB14-KD-1) and the negative control shRNA (NC) (RAB14-KD-1 vs. NC). Only total RNA of high quality and integrity was subjected to further processing after purification, defined as a 260/280 absorption ratio of >1.8 on spectrophotometry using the a NanoDrop 1000 spectrophotometer (NanoDrop, Wilmington, DE, USA), and a relative intensity noise (RIN) value of >8.0 on an electrophoretic analysis using Bioanalyzer 2100 (Agilent Technologies, Santa Clara, CA, USA). In vitro transcription was performed using the Ambion MessageAmp Premier Enhanced assay protocol (Ambion, Austin, TX, USA) starting with 500 ng of purified total RNA. Confirmation of cRNA diversity was obtained using the Bioanalyzer 2100 to generate an electrophoretogram for each in vitro transcription (IVT) reaction regarding the sample yield, integrity, and size diversity against Universal Human Reference RNA (Stratagene, La Jolla, CA, USA). Fifteen micrograms of purified, amplified, and biotin-labeled cRNA was fragmented and hybridized onto Affymetrix Human Genome HGU133A 2.0 arrays (Affymetrix, Santa Clara, CA, USA) for 18 h. Arrays were washed, stained, and scanned on the Affymetrix Fluidics Station 450 and Scanner 3000 immediately after completion of hybridization. Array data were normalized using a robust log-scale multi-array analysis and were analyzed by R-Project software. Gene expression was considered significant when the multiple of change value was >2.0 (RAB14-KD-1 vs. NC) and *p* < 0.05. Identification of cancer development and MAPK pathways was determined by a gene ontology analysis based on classification of gene numbers and was used to perform a functional enrichment analysis. Analyses included biological processes, cellular components, and molecular functions. For statistical analysis of gene oncology, a gene set enrichment analysis and Fisher’s exact analyses were performed. To investigate the relationship between RAB14 and autophagy in bladder cancer, We downloaded gene expression data from TCGA (https://portal.gdc.cancer.gov/ projects/TCGA BLCA) and performed Gene Set Enrichment Analysis (GSEA) with the acquired microarray data to analyze whether RAB14 expression is correlated with that of autophagy genes.

### Immunohistochemical (IHC) analysis

RAB14 levels were evaluated in paraffin-embedded tissue sections. With regards to IHC assay, overnight incubation of sections was done with primary antibodies RAB14 (1:100, ab28639), LC3B (1:100, ab239416), Beclin1 (1:200, ab210498) at 4 °C. Then, they were incubated for 40 min with a biotin-labeled secondary antibody (1:100) at 37 °C. They were incubated in the presence of DAB, counterstained with hematoxylin, after which light microscopy was used for visualization. To establish the negative controls, the primary antibody was replaced with phosphate-buffered saline (PBS) in all samples. Scoring of RAB14 was done with IHC using a semi-quantitative system with a staining index (SI). The formula for SI calculation was: (positive cell percentage score) × (staining intensity score). Staining intensities were scored from 0 to 3 as: 0, background color; 1, light-yellow, slightly higher than the background color; 2, brown, obviously higher than the background color; and 3, tan. Positive cell percentages were classified from 0 to 3, whereby, 0 (no positive cells), 1 ( < 10%), 2 (11%~50%) and 3 (51%~75%). Staining outcome was called based SI values: 0~2 (negative), 3~4 (weak), 5~8 (moderate), and 9~12 (strong).

### Western blot analysis

Proteins were isolated from stably-transfected T24 cells and tumor tissues from nude-mice, after which separation was done by sodium dodecyl sulfate-polyacrylamide gel electrophoresis (SDS-PAGE). Proteins in the gels were moved to polyvinylidene difluoride (PVDF) membranes, which were blocked for 1–2 h using 5% skim milk followed by overnight incubation in the presence of primary antibodies at 4 °C. After washing with PBST, incubation of the blots with goat anti-mouse or anti-rabbit antibodies (1:5000; Abcam, Redlands, CA, USA) at 37 °C was done for 1 h and were subsequently evaluated with a Bio-Rad gel imaging system (Bio-Rad, Hercules, CA, USA) using a WB kit (Advansta, Menlo Park, USA). The Image J software (ver. 6.0; National Institutes of Health, Bethesda, MD, USA) was used to analyze the results, which were normalized to β-actin.

### Electron microscopy

For 4 h, cells were fixed in 2.5% glutaraldehyde (Solarbio, Beijing, China) at 4 °C, pre-embedded in 1% agar to maintain integrity, washed with PBS, and thereafter post-fixed for 2 h in the presence of a 1% OsO4 buffer at 4°C. After washing, cells were dehydrated in graded ethanol concentrations and embedded using Epon812 epoxy resin. Ultrathin sections (90 nm) were obtained on copper grids, double-stained with 0.2% lead citrate and 1% uranyl acetate, followed by examination by HT7800 transmission electron microscopy (Hitachi, Japan).

### Phalloidin staining

F-actin is the main component of microflaments and is capable of binding with phalloidin. For phalloidin staining, cells growing on the glass slide were fixed in 4% formaldehyde at room temperature for 20 min, and then we rinsed the slides 3 times with phosphate-buffered saline (PBS). After that, phalloidin-conjugate working solution (Phalloidin-iFluor 555 Reagent, ab176756) was added on the slides and incubated at 37 °C for an hour. The cells were washed three times with PBS to remove the unbound phalloidin conjugate. DAPI (blue) was applied to stain nuclear DNA. Finally, we observed cell morphology change under the confocal laser-scanning microscopy, and took representative images.

### Immunofluorescence staining

The BLCA cells cultured on cover slips were fixed with 4% paraformaldehyde for 15 min, and then permeabilized for 10 min with 0.5% TritonX100. The cells were blocked with 3% BSA for 30 min. the cells were incubated with anti-LC3B (1:1000, Abcam) or anti-Beclin1 (1:1000, Abcam) primary antibodies for 1 h at 37 °C. Subsequently, the slides were washed and then incubated with corresponding IgG H&L (Alexa Fluor® 488) secondary antibodies (Abcam) for 1 h at 37 °C. Nuclei were stained using 4′,6-diamidino-2-phenylindole (DAPI). The slides were sealed with sealing solution containing anti fluorescence quenching agent, and then observe and collect the image under a fluorescence microscope (DM2500, Leica).

### Statistical analysis

Unless otherwise stated, assays were repeated thrice and findings shown as mean ± SD. SPSS 22.0 was used for data analyses. Statistical significance was determined with a two-tailed Student’s *t*-test and χ^2^ test, with *P* < 0.05 accepted as being statistically significant.

### Supplementary information

Figure S1

Table S1_1

Table S1_2

Table S1_3

Table S2_1

Table S2_2

Table S2_3

Table S2_4

Table S3

Original WB

## Data Availability

The data that support the findings of this study are available from the corresponding author upon reasonable request.
