# Supplementary material for: RAB14 promotes epithelial-mesenchymal transition in bladder cancer through autophagy‑dependent AKT signaling pathway
Source: Cell Death Discov. 2023 Aug 9;9:292. doi: 10.1038/s41420-023-01579-8 (PMC10412633; doi:10.1038/s41420-023-01579-8)
Supplement: Supplementary file 1 — Figure S1 [file 41420_2023_1579_MOESM1_ESM.pdf]

RAB14 promotes epithelial-mesenchymal transition in bladder cancer through autophagy‑dependent AKT signaling pathway

Huanhuan Deng[1,#], Leihong Deng[2,#], Haichao Chao[3], Zhaojun Yu[1], Jianbiao Huang[1], Zhen Song[1], Lifen Peng[4,*], Tao Zeng[1,3,*]

[1]Donghu Campus, Medical College of Nanchang University, Nanchang, Jiangxi 330006, China

[2]Department of Ultrasound Medicine, The First Affiliated Hospital of Nanchang University, Nanchang, Jiangxi 330006, China

[3]Department of Urology, The Second Affiliated Hospital of Nanchang University, Nanchang, Jiangxi 330006, China

[4]Department of Clinical trial center，Jiangxi Provincial People’s Hospital , No. 152, Aiguo Road, Nanchang, Jiangxi 330006, China.

Supplementary Figure and figure legend

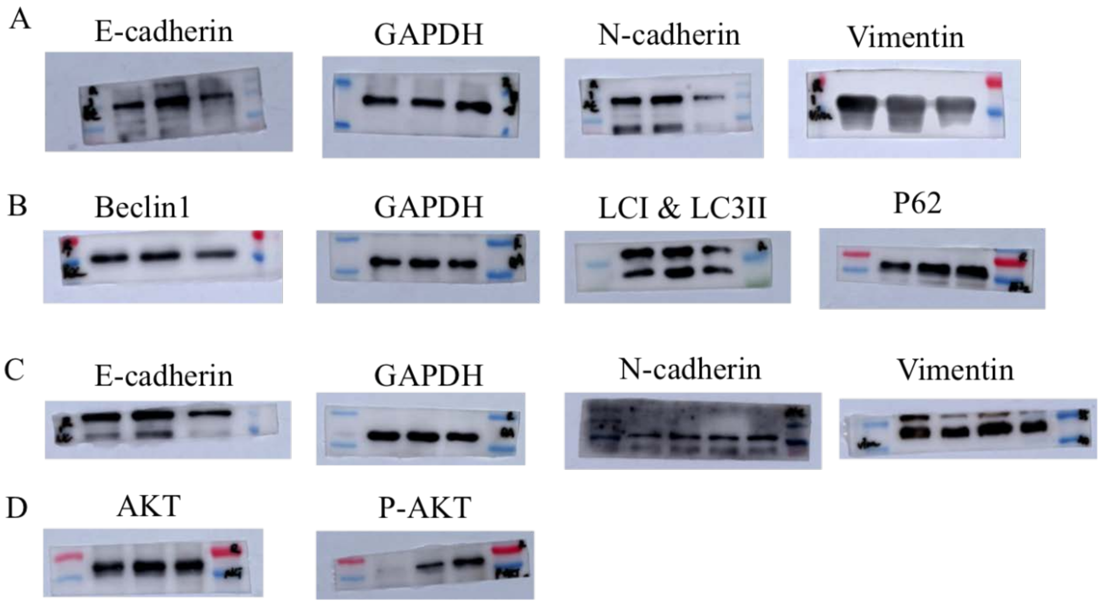

Figure S1. Western blot original gel image in this study. (A) Western blot original gel image in Figure 4D. (B) Western blot original gel image in Figure 6C. (C) Western blot original gel image in Figure 7D. (D)Western blot original gel image in Figure 8B.
