# Supplementary figures and images for: RAB14 promotes epithelial-mesenchymal transition in bladder cancer through autophagy‑dependent AKT signaling pathway
Source: Cell Death Discov. 2023 Aug 9;9:292. doi: 10.1038/s41420-023-01579-8 (PMC10412633; doi:10.1038/s41420-023-01579-8)

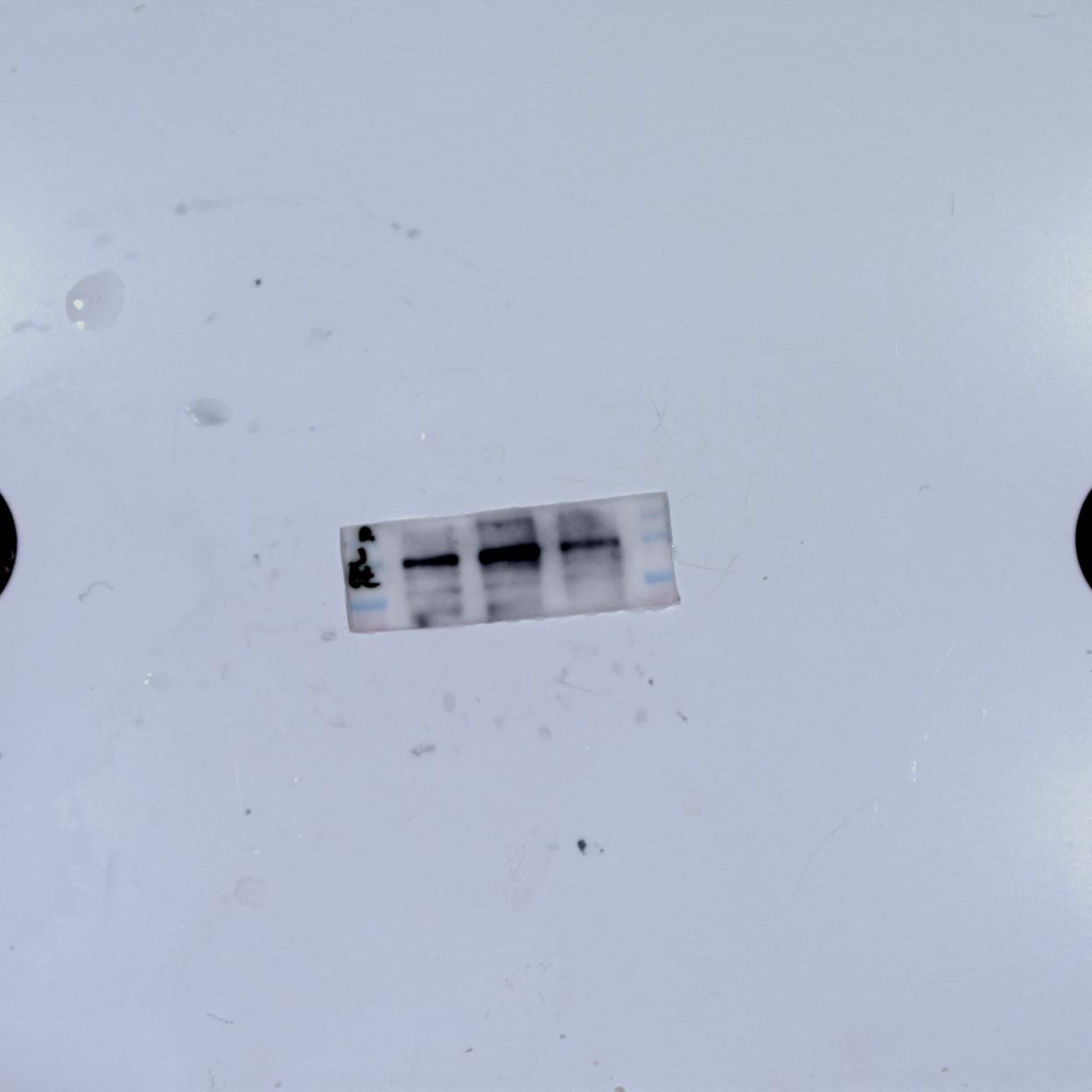

Supplement: Supplementary file 10 — Original WB [file 41420_2023_1579_MOESM10_ESM.zip › Original WB/Figure 4D/R-ECAD 20220329_195801_Ch已用/R-ECAD 20220329_195801_Ch+Marker.jpg]

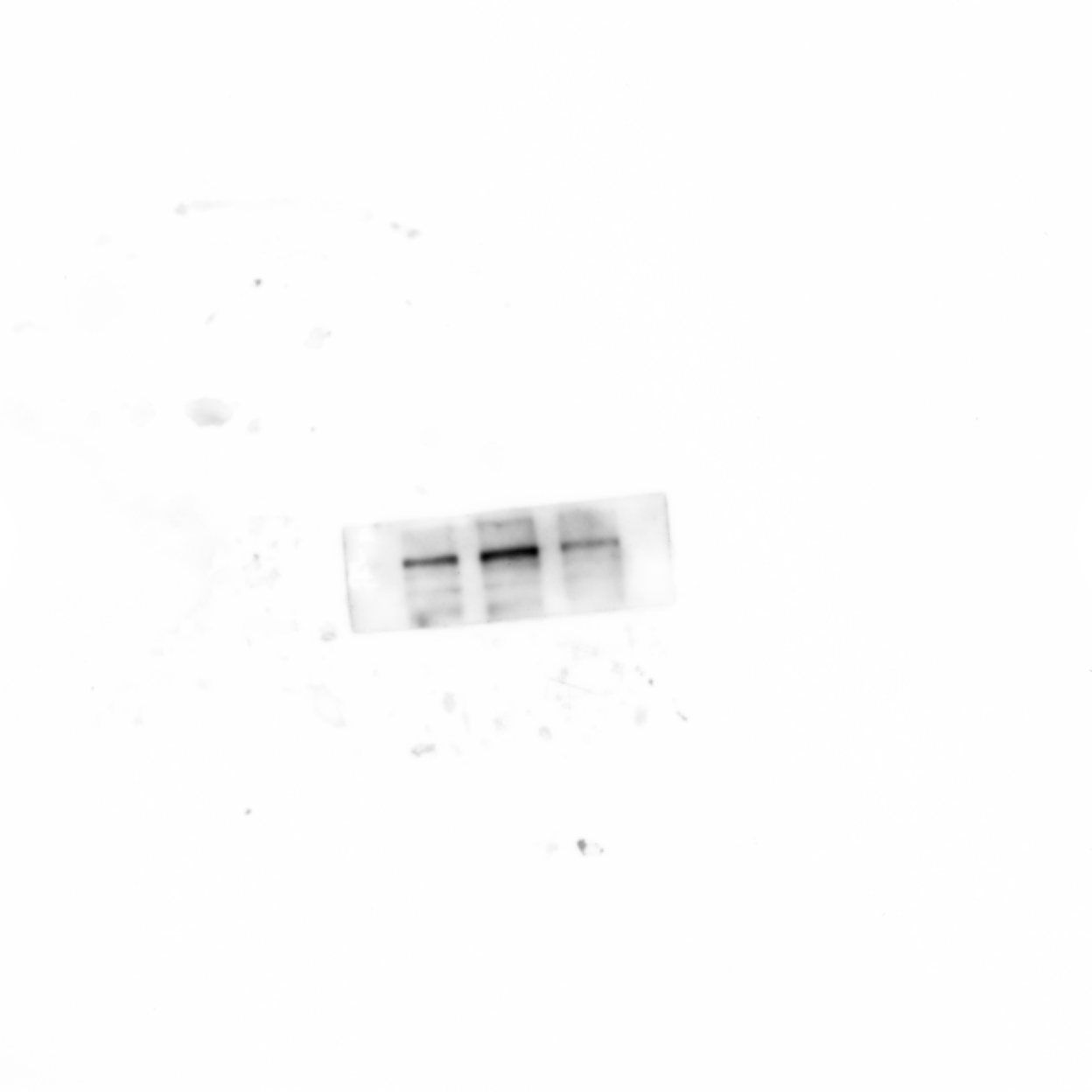

Supplement: Supplementary file 10 — Original WB [file 41420_2023_1579_MOESM10_ESM.zip › Original WB/Figure 4D/R-ECAD 20220329_195801_Ch已用/R-ECAD 20220329_195801_Ch.tif]

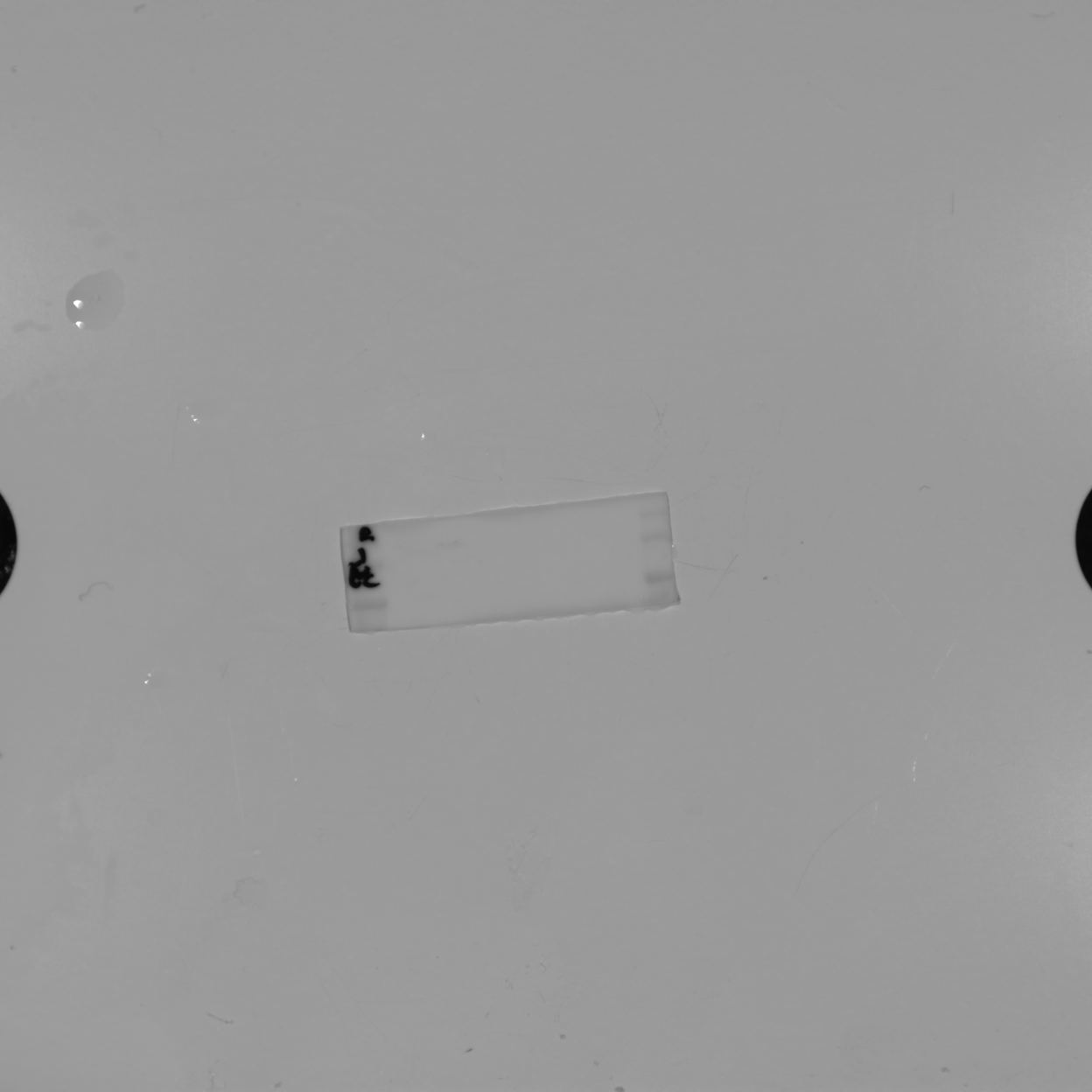

Supplement: Supplementary file 10 — Original WB [file 41420_2023_1579_MOESM10_ESM.zip › Original WB/Figure 4D/R-ECAD 20220329_195801_Ch已用/R-ECAD 20220329_195801_Ch_Marker.tif]

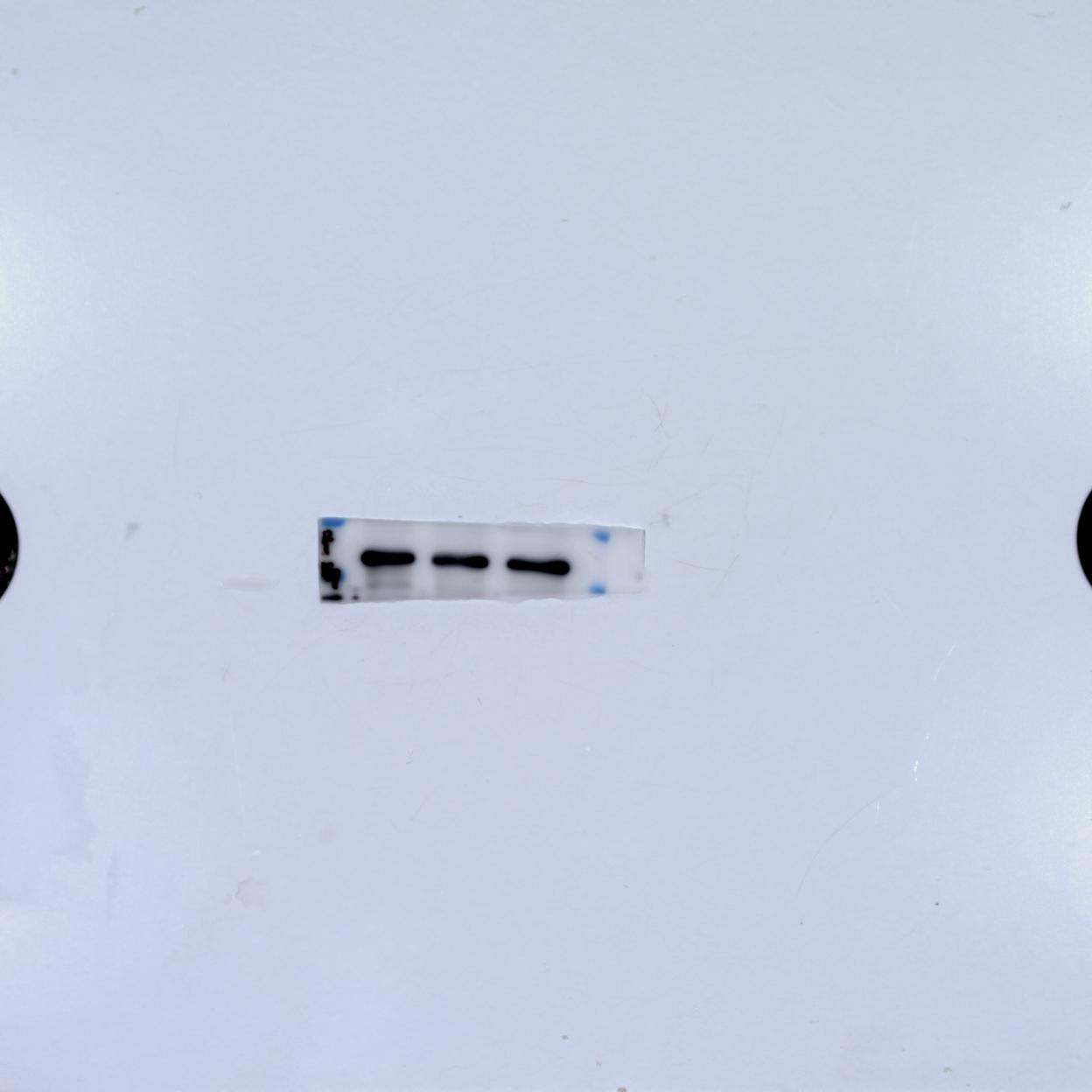

Supplement: Supplementary file 10 — Original WB [file 41420_2023_1579_MOESM10_ESM.zip › Original WB/Figure 4D/R-GA3 20220327_184735_Ch已用/R-GA3 20220327_184735_Ch+Marker.jpg]

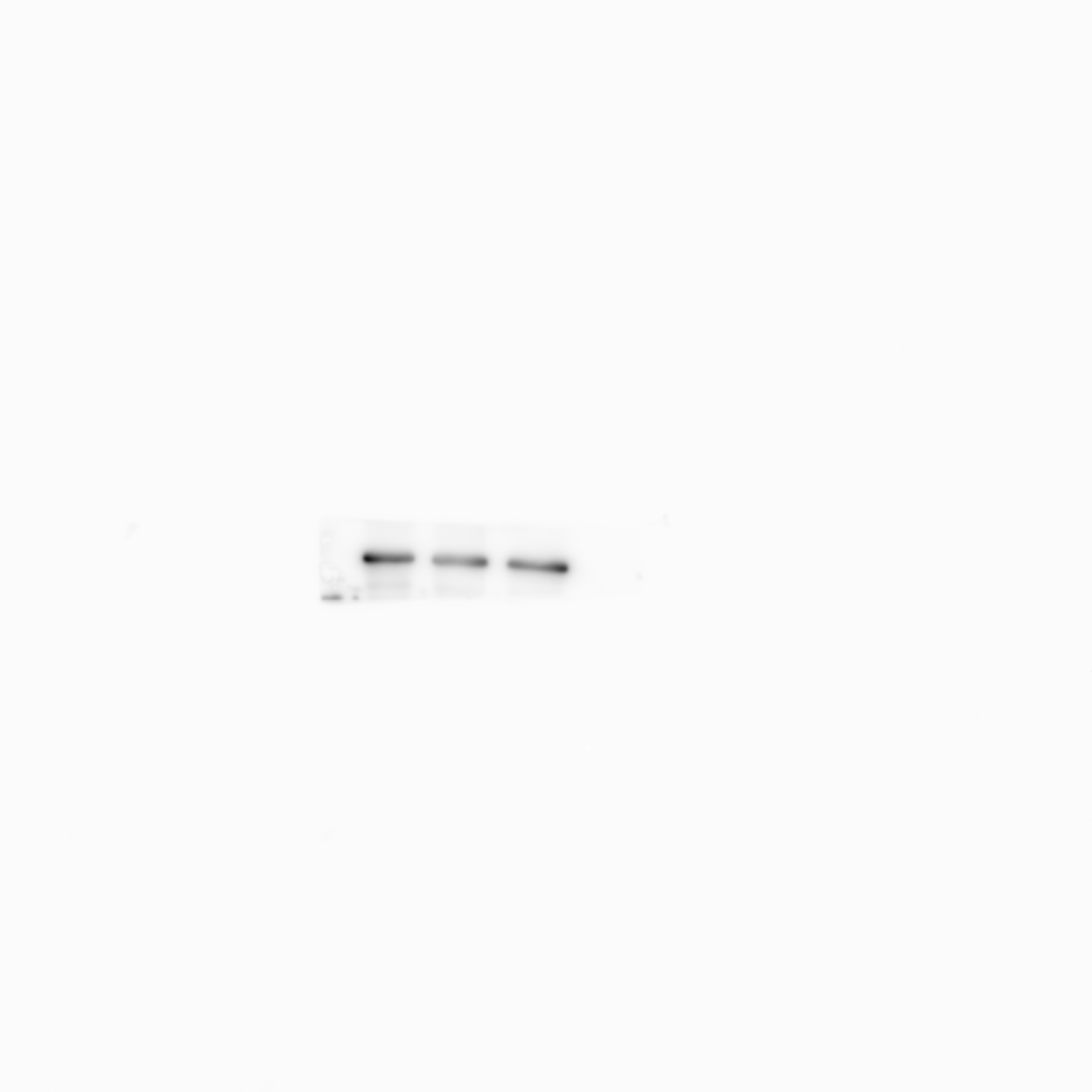

Supplement: Supplementary file 10 — Original WB [file 41420_2023_1579_MOESM10_ESM.zip › Original WB/Figure 4D/R-GA3 20220327_184735_Ch已用/R-GA3 20220327_184735_Ch.tif]

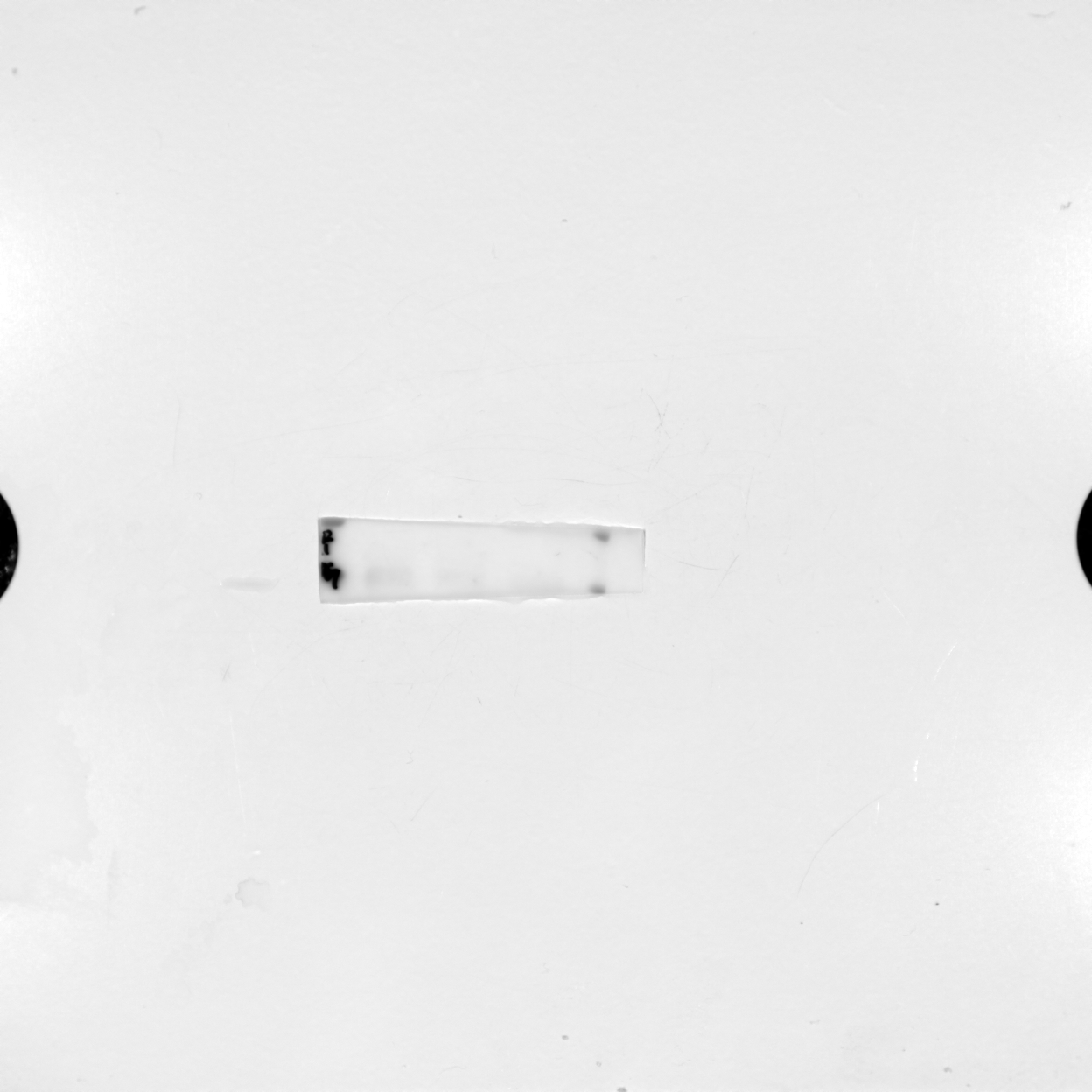

Supplement: Supplementary file 10 — Original WB [file 41420_2023_1579_MOESM10_ESM.zip › Original WB/Figure 4D/R-GA3 20220327_184735_Ch已用/R-GA3 20220327_184735_Ch_Marker.tif]

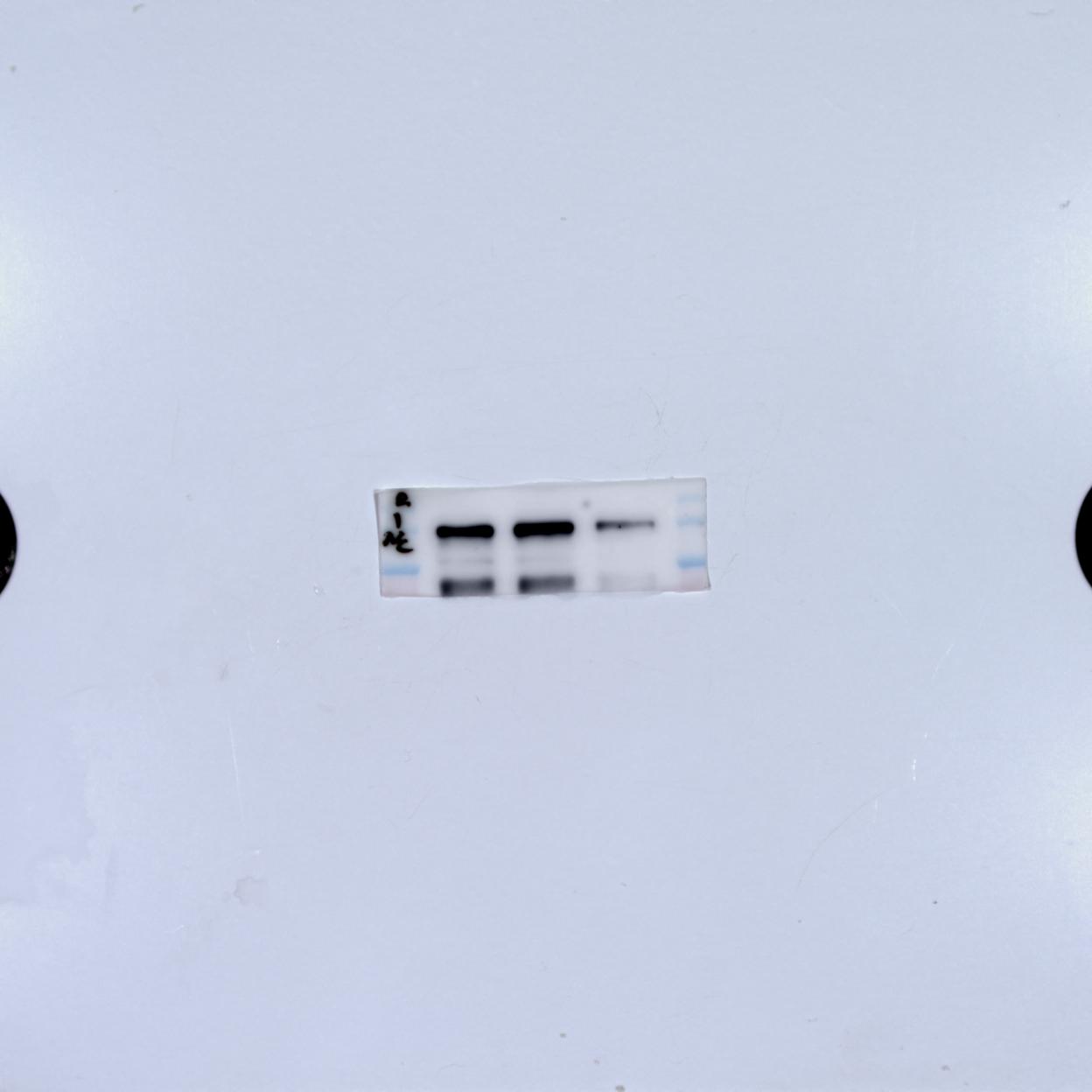

Supplement: Supplementary file 10 — Original WB [file 41420_2023_1579_MOESM10_ESM.zip › Original WB/Figure 4D/R-ncad1 20220327_191608_Ch已用/R-ncad1 20220327_191608_Ch+Marker.jpg]

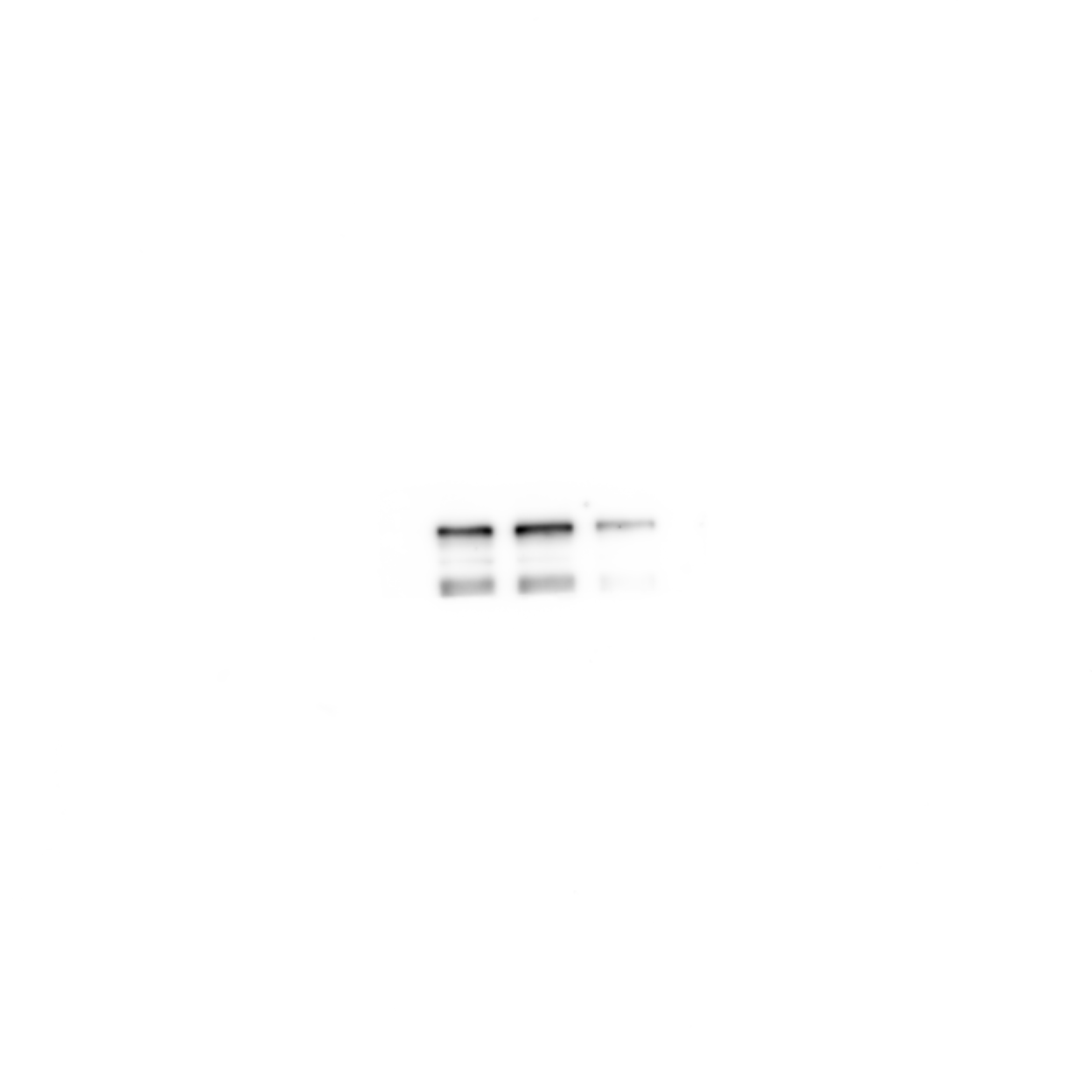

Supplement: Supplementary file 10 — Original WB [file 41420_2023_1579_MOESM10_ESM.zip › Original WB/Figure 4D/R-ncad1 20220327_191608_Ch已用/R-ncad1 20220327_191608_Ch.tif]

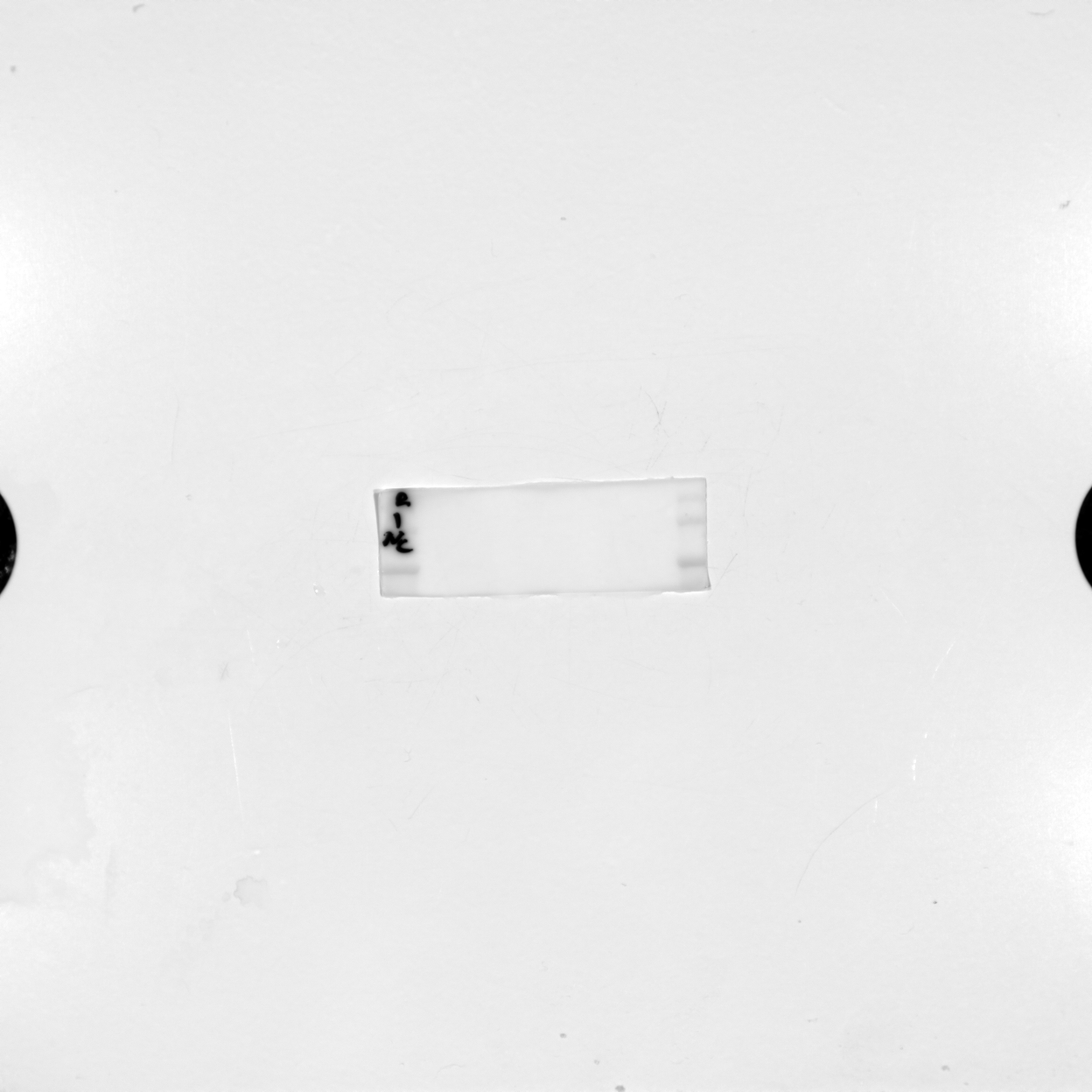

Supplement: Supplementary file 10 — Original WB [file 41420_2023_1579_MOESM10_ESM.zip › Original WB/Figure 4D/R-ncad1 20220327_191608_Ch已用/R-ncad1 20220327_191608_Ch_Marker.tif]

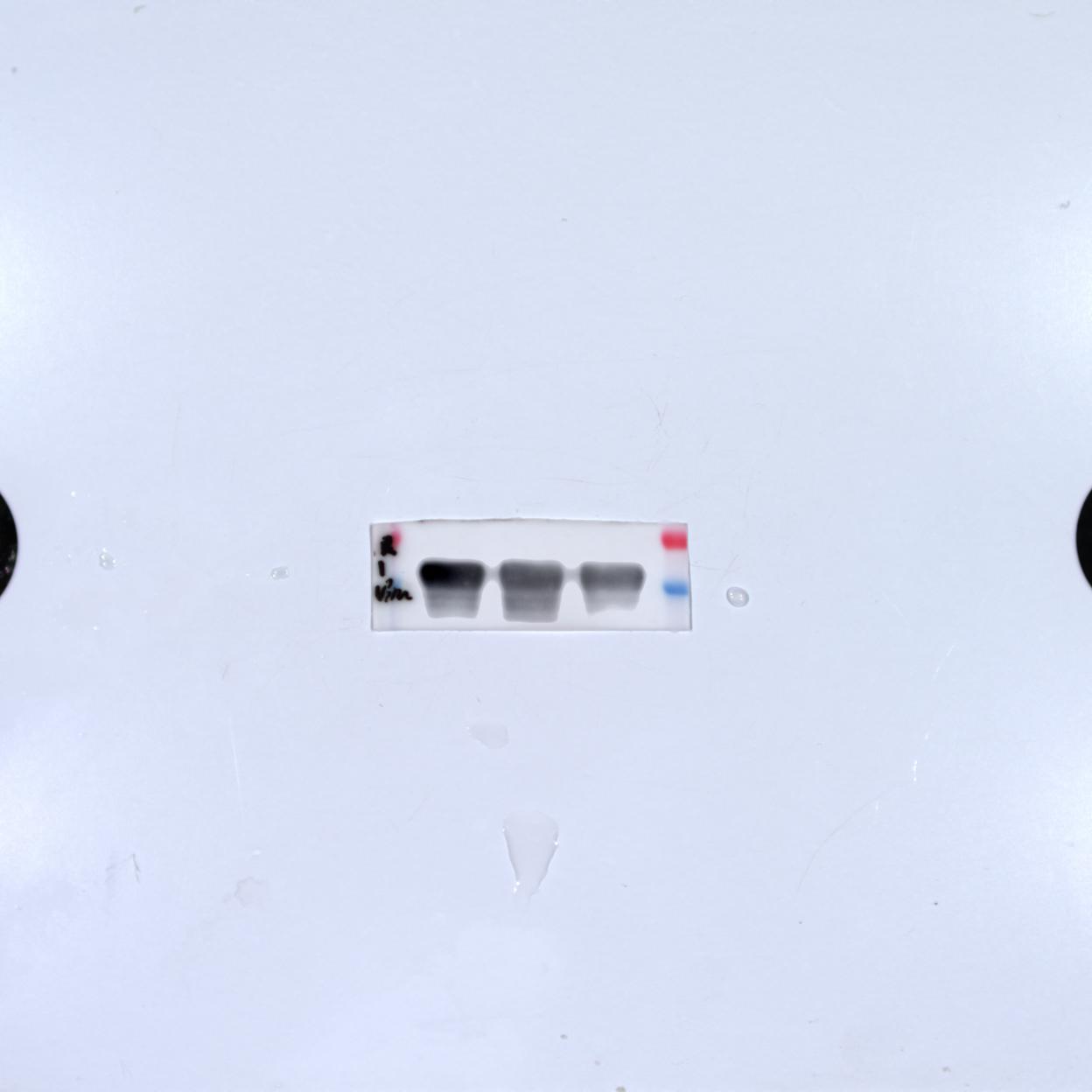

Supplement: Supplementary file 10 — Original WB [file 41420_2023_1579_MOESM10_ESM.zip › Original WB/Figure 4D/R-vim 20220327_193613_Ch已用/R-vim 20220327_193613_Ch+Marker.jpg]

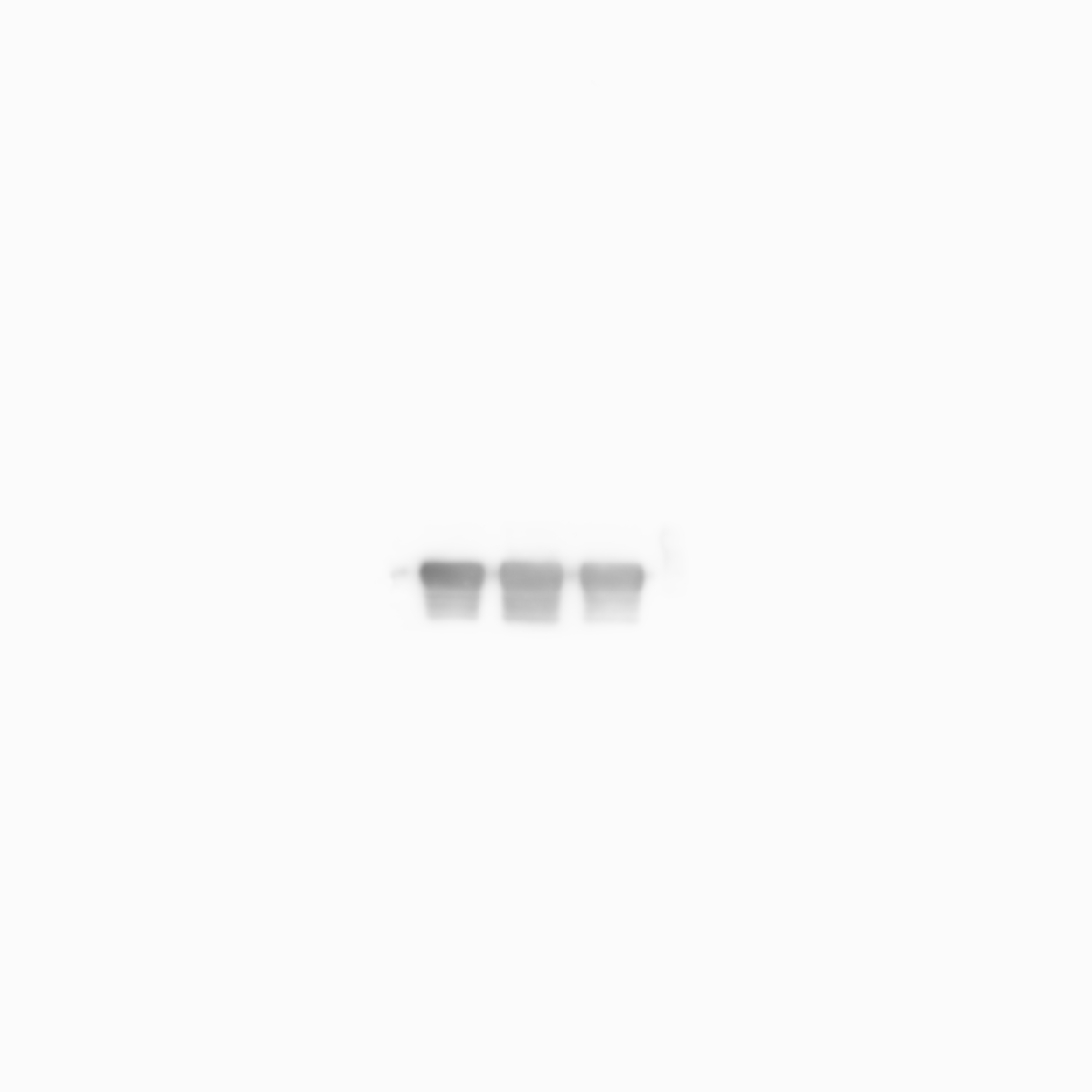

Supplement: Supplementary file 10 — Original WB [file 41420_2023_1579_MOESM10_ESM.zip › Original WB/Figure 4D/R-vim 20220327_193613_Ch已用/R-vim 20220327_193613_Ch.tif]

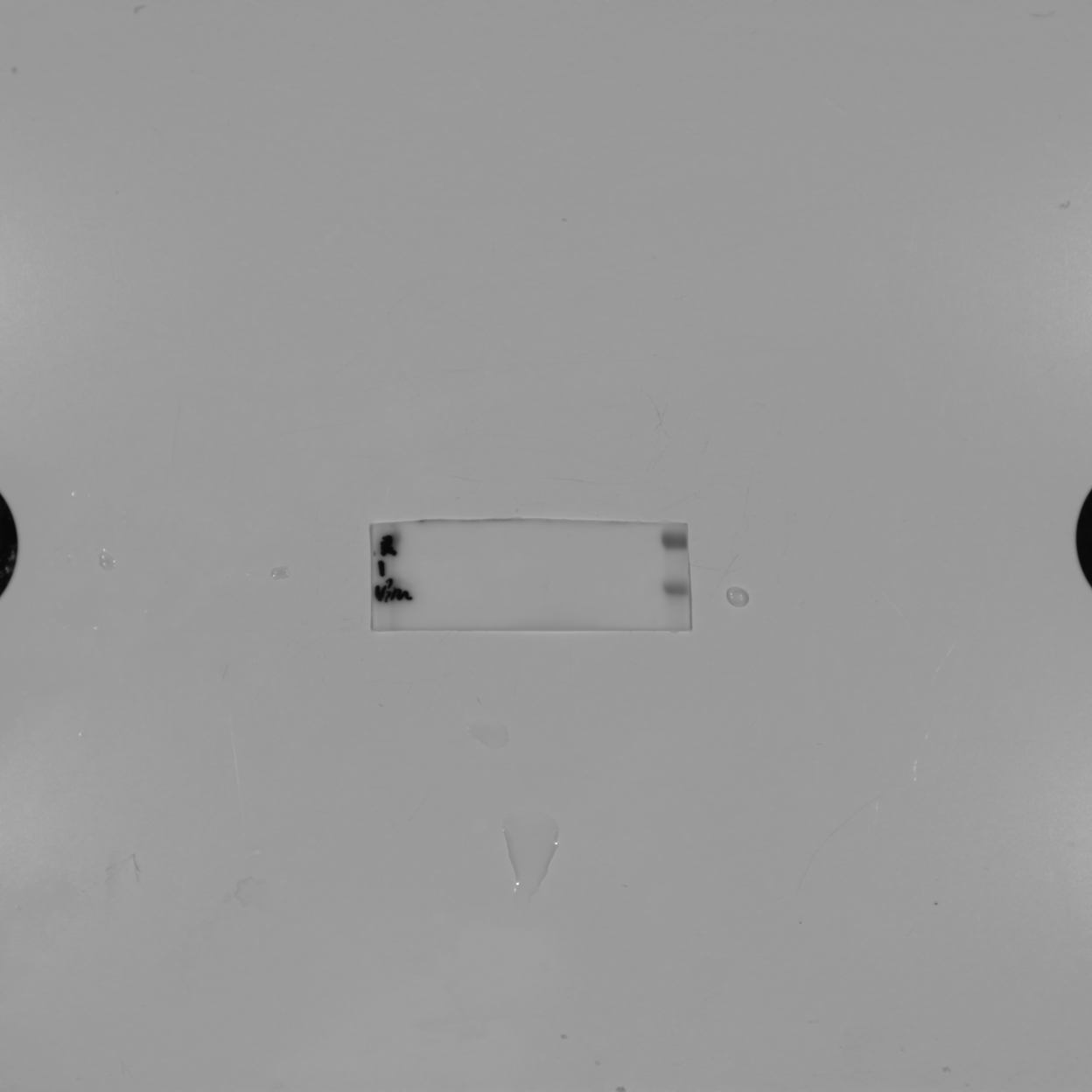

Supplement: Supplementary file 10 — Original WB [file 41420_2023_1579_MOESM10_ESM.zip › Original WB/Figure 4D/R-vim 20220327_193613_Ch已用/R-vim 20220327_193613_Ch_Marker.tif]

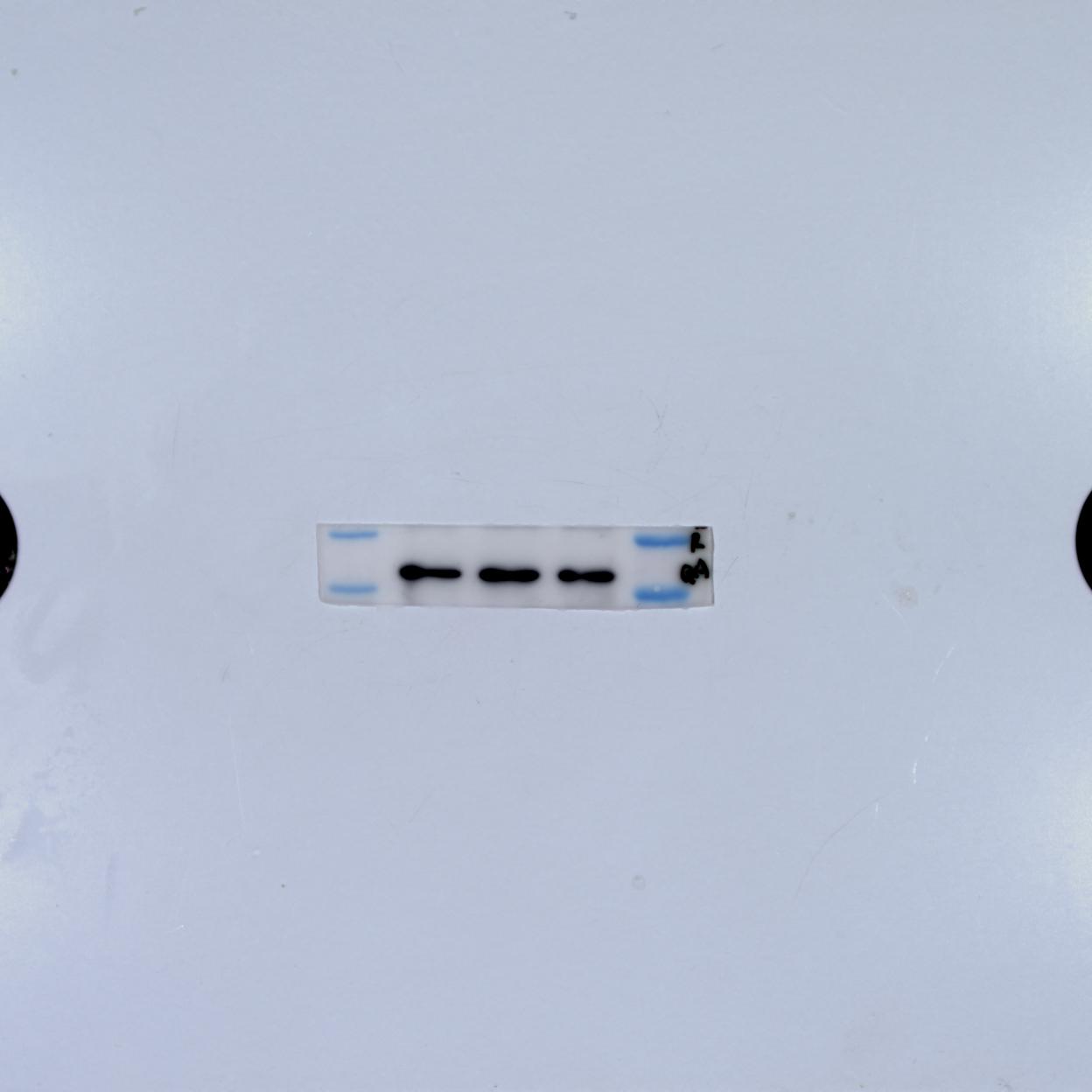

Supplement: Supplementary file 10 — Original WB [file 41420_2023_1579_MOESM10_ESM.zip › Original WB/Figure 6C/R-GA3 20220323_175859_Ch已用/R-GA3 20220323_175859_Ch+Marker.jpg]

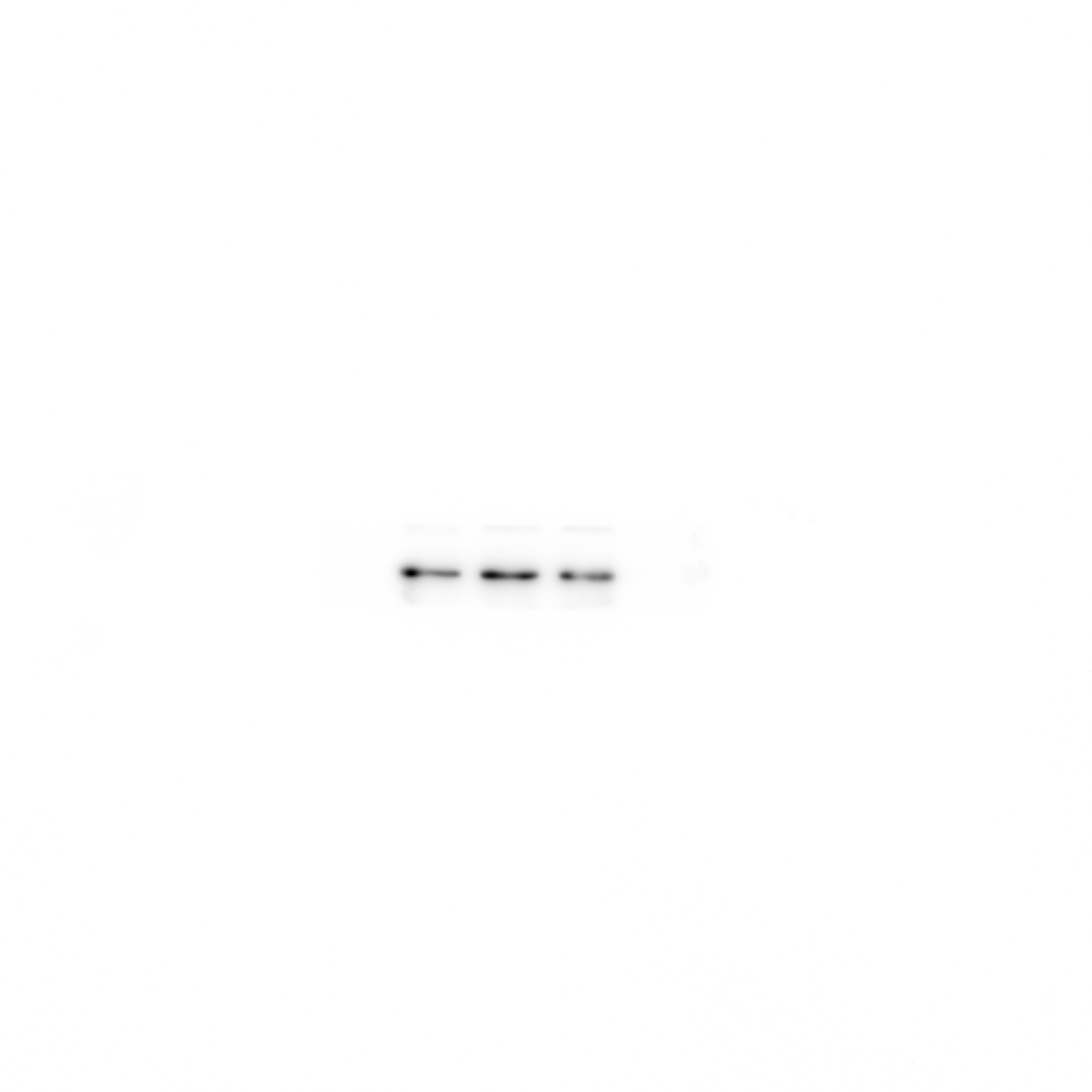

Supplement: Supplementary file 10 — Original WB [file 41420_2023_1579_MOESM10_ESM.zip › Original WB/Figure 6C/R-GA3 20220323_175859_Ch已用/R-GA3 20220323_175859_Ch.tif]

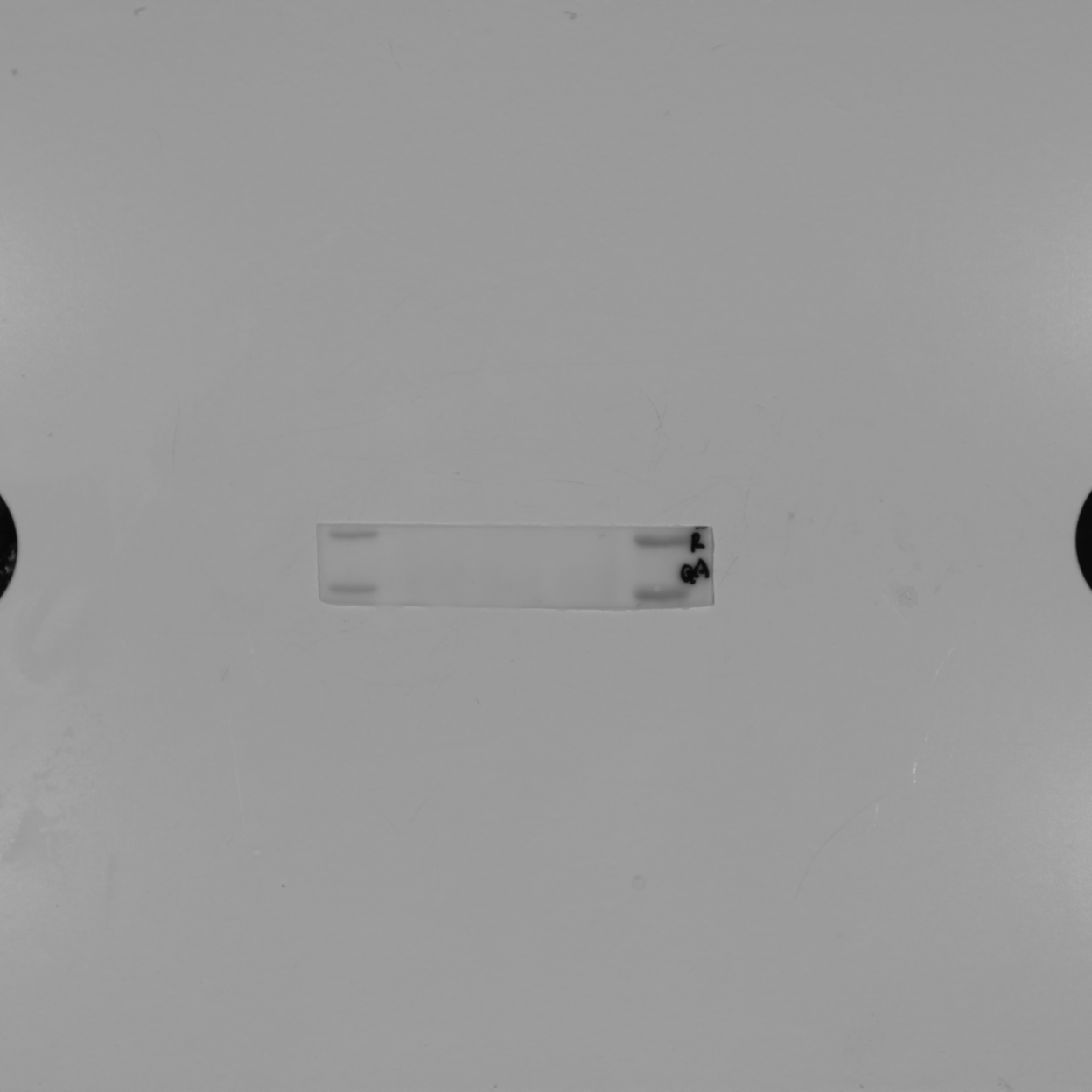

Supplement: Supplementary file 10 — Original WB [file 41420_2023_1579_MOESM10_ESM.zip › Original WB/Figure 6C/R-GA3 20220323_175859_Ch已用/R-GA3 20220323_175859_Ch_Marker.tif]

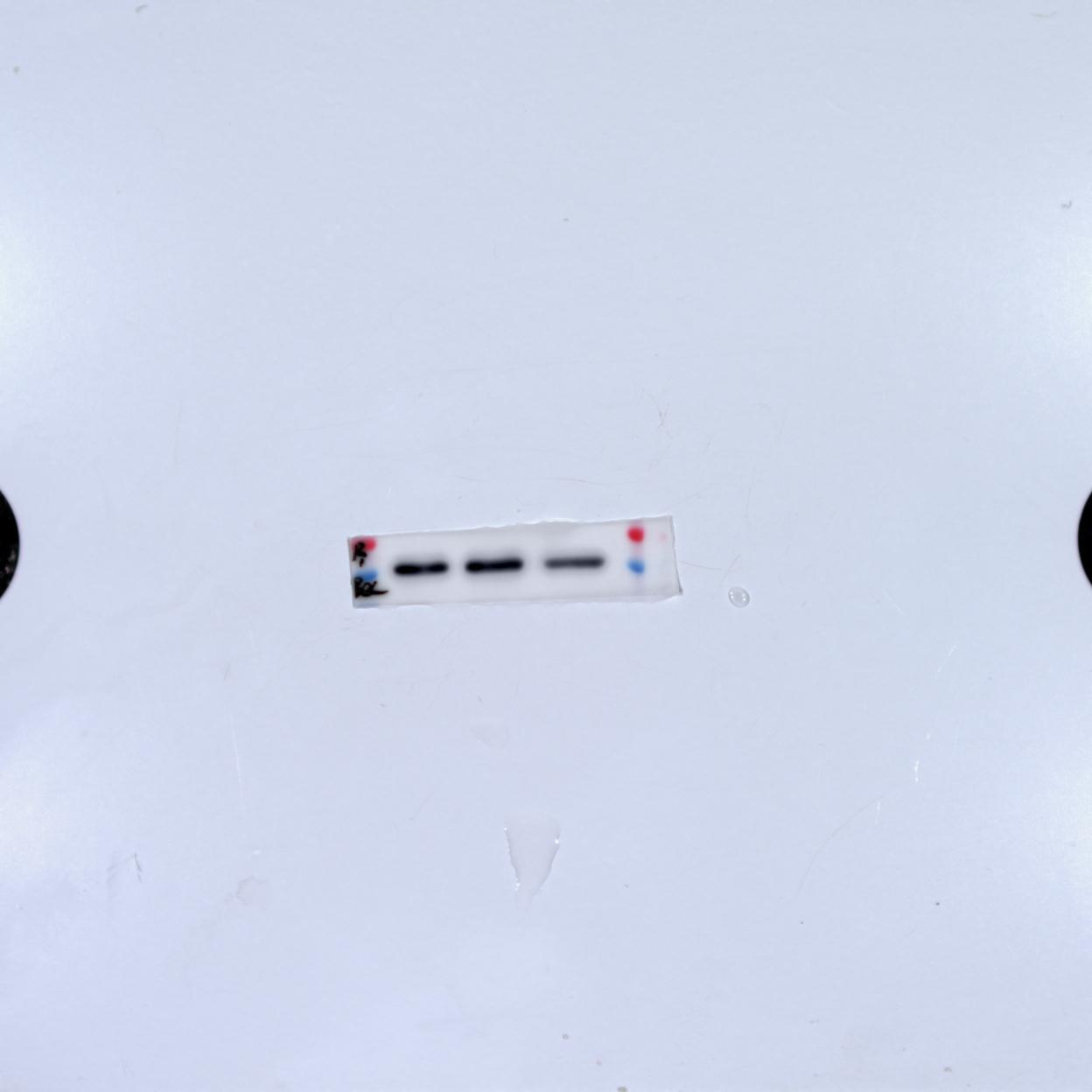

Supplement: Supplementary file 10 — Original WB [file 41420_2023_1579_MOESM10_ESM.zip › Original WB/Figure 6C/R-bec 20220327_193757_Ch已用/R-bec 20220327_193757_Ch+Marker.jpg]

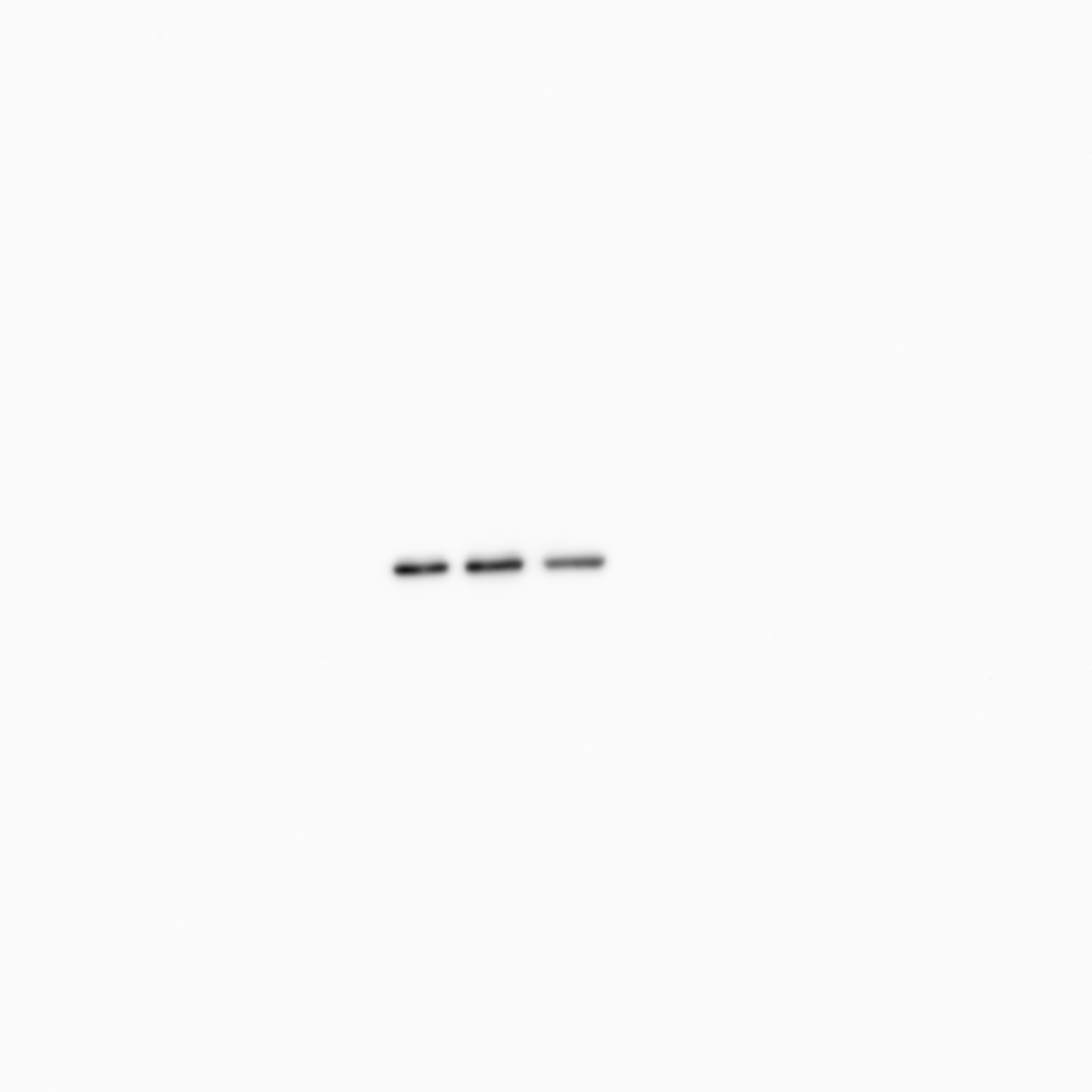

Supplement: Supplementary file 10 — Original WB [file 41420_2023_1579_MOESM10_ESM.zip › Original WB/Figure 6C/R-bec 20220327_193757_Ch已用/R-bec 20220327_193757_Ch.tif]

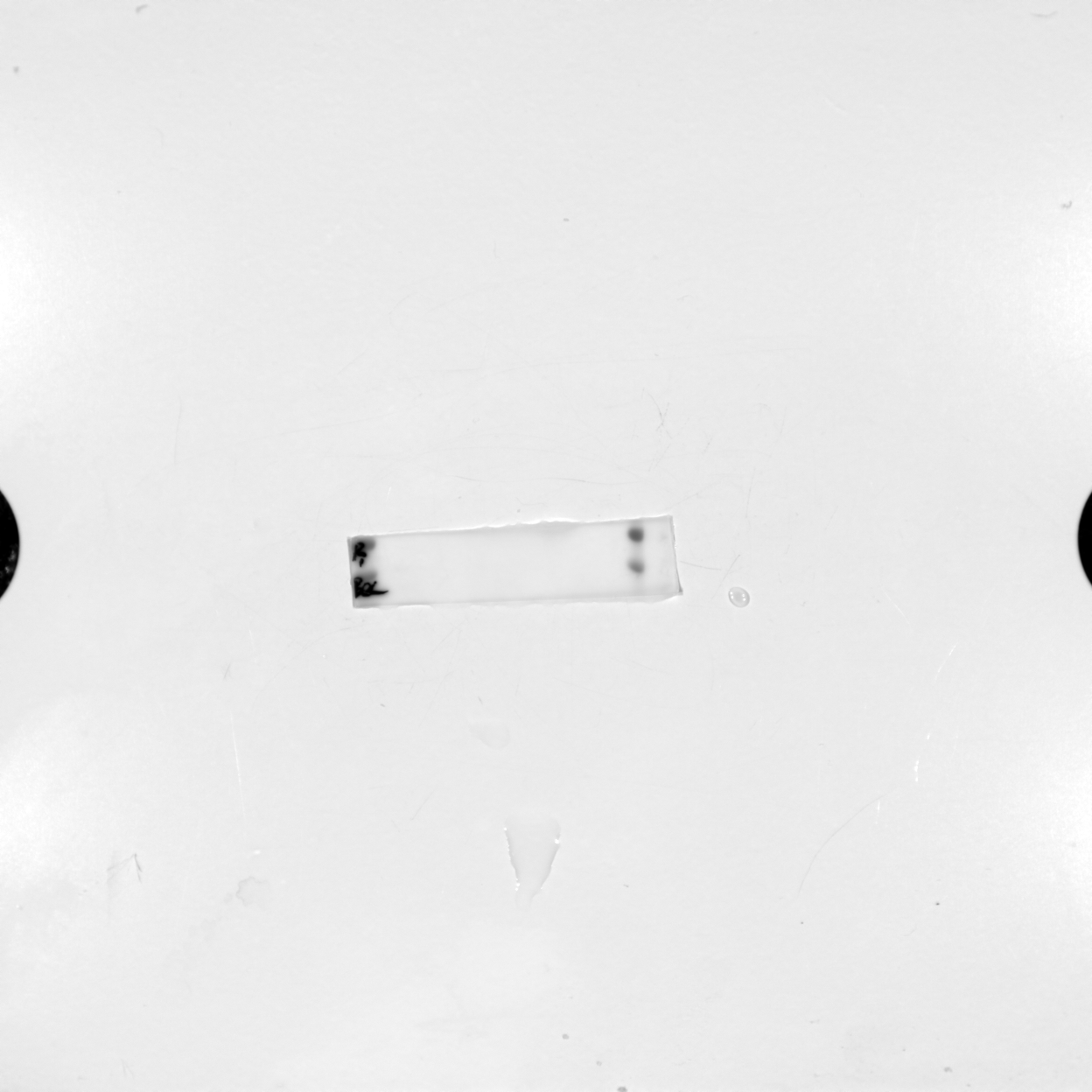

Supplement: Supplementary file 10 — Original WB [file 41420_2023_1579_MOESM10_ESM.zip › Original WB/Figure 6C/R-bec 20220327_193757_Ch已用/R-bec 20220327_193757_Ch_Marker.tif]

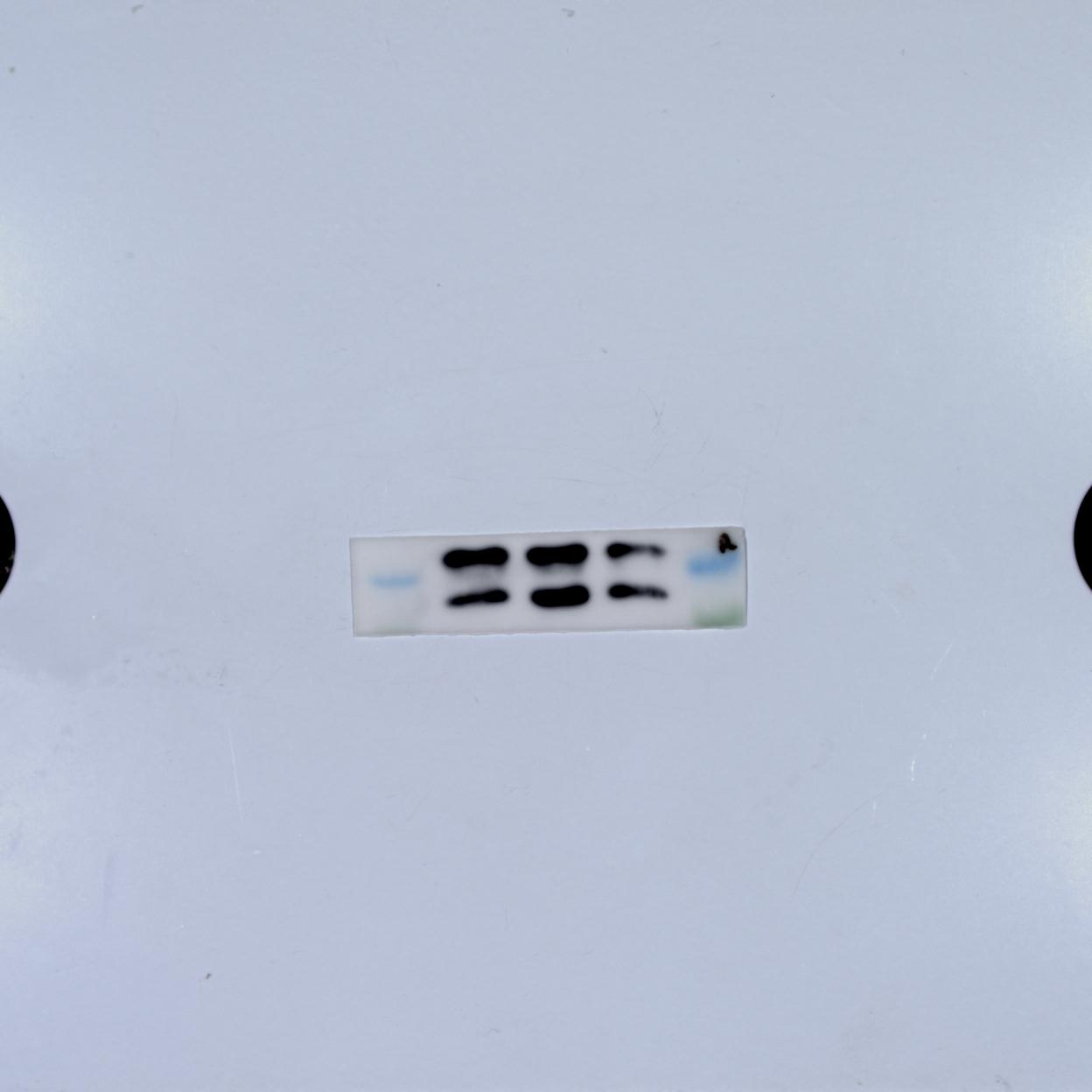

Supplement: Supplementary file 10 — Original WB [file 41420_2023_1579_MOESM10_ESM.zip › Original WB/Figure 6C/R-lc3-1 20220323_182641_Ch已用/R-lc3-1 20220323_182641_Ch+Marker.jpg]

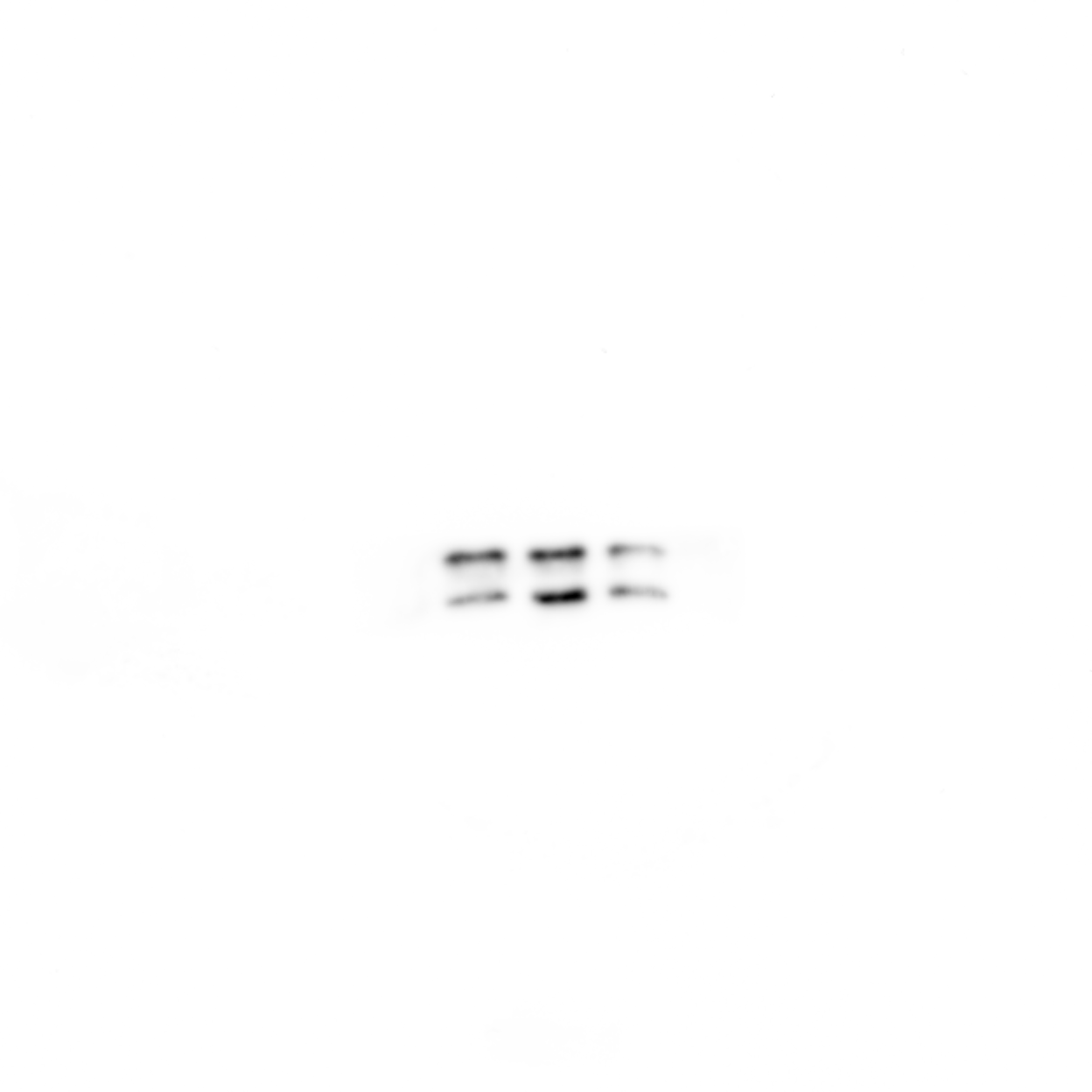

Supplement: Supplementary file 10 — Original WB [file 41420_2023_1579_MOESM10_ESM.zip › Original WB/Figure 6C/R-lc3-1 20220323_182641_Ch已用/R-lc3-1 20220323_182641_Ch.tif]

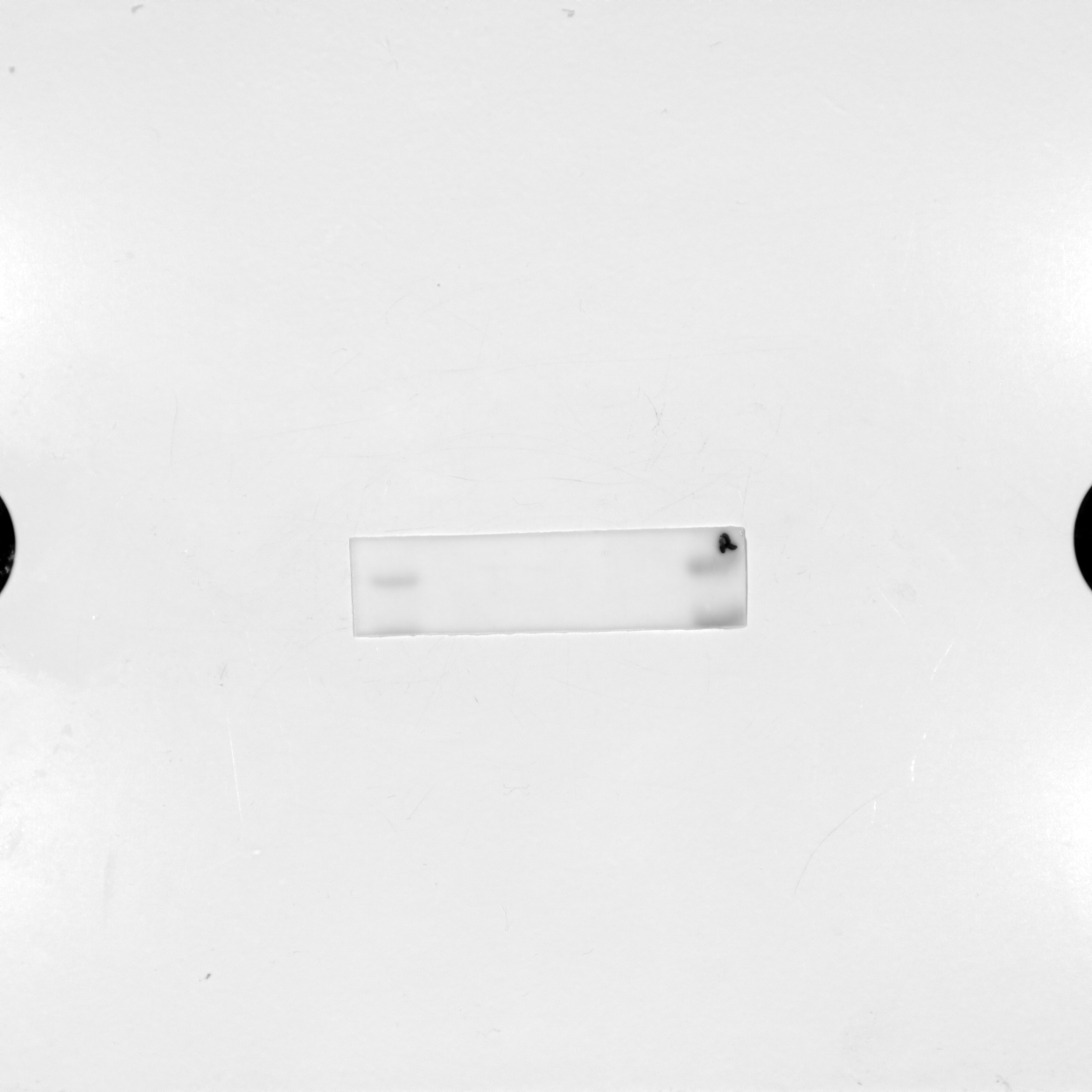

Supplement: Supplementary file 10 — Original WB [file 41420_2023_1579_MOESM10_ESM.zip › Original WB/Figure 6C/R-lc3-1 20220323_182641_Ch已用/R-lc3-1 20220323_182641_Ch_Marker.tif]

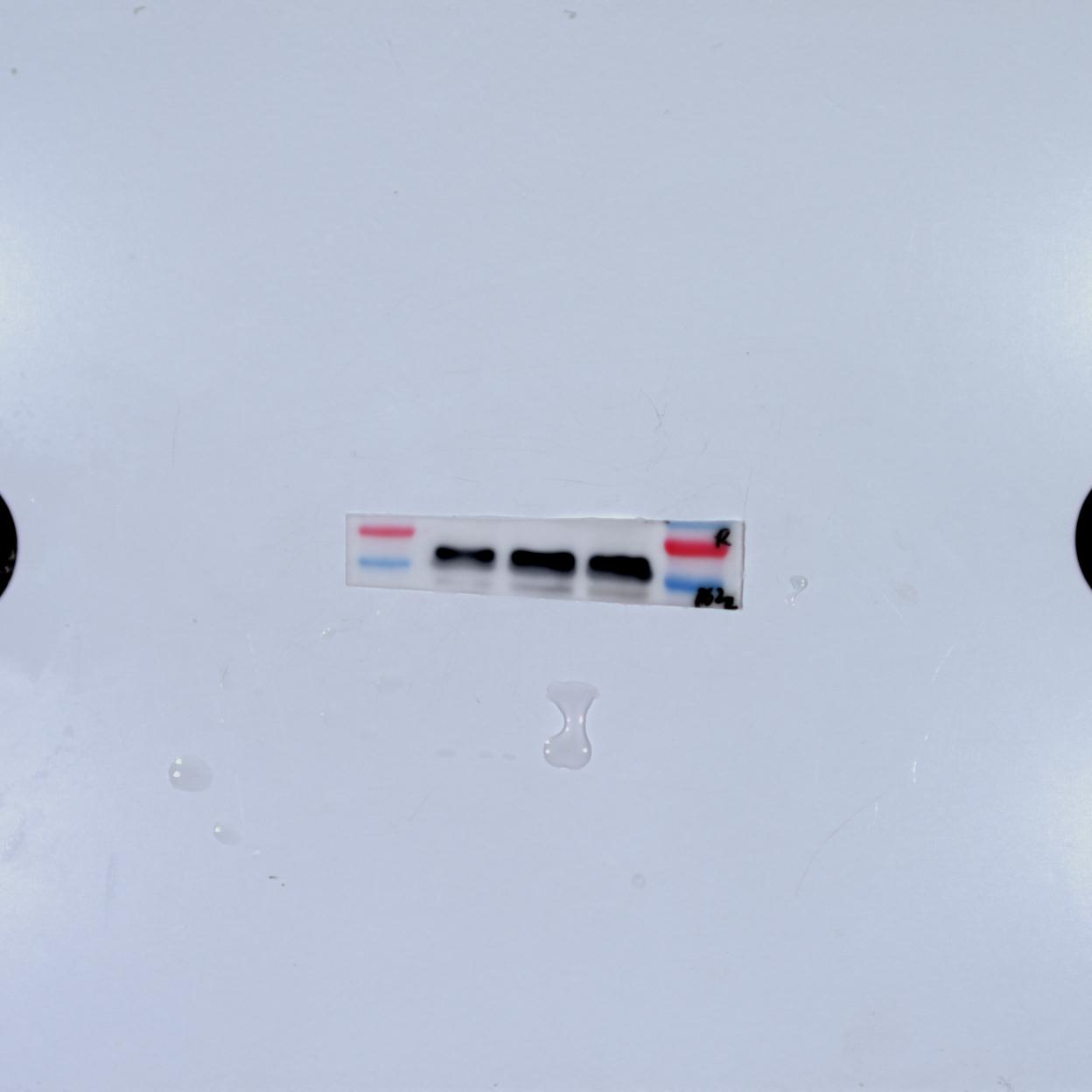

Supplement: Supplementary file 10 — Original WB [file 41420_2023_1579_MOESM10_ESM.zip › Original WB/Figure 6C/R-p62 20220323_180856_Ch已用/R-p62 20220323_180856_Ch+Marker.jpg]

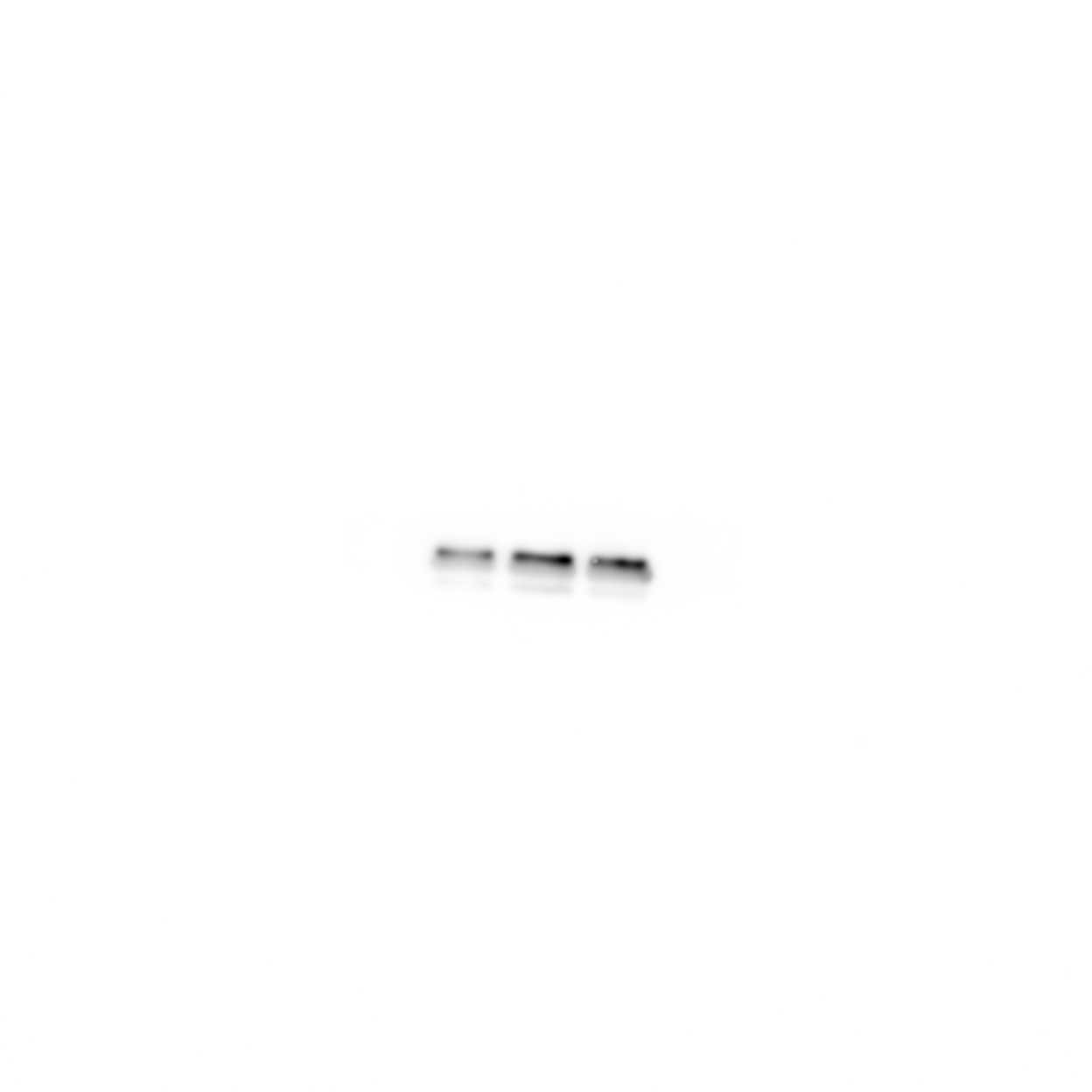

Supplement: Supplementary file 10 — Original WB [file 41420_2023_1579_MOESM10_ESM.zip › Original WB/Figure 6C/R-p62 20220323_180856_Ch已用/R-p62 20220323_180856_Ch.tif]

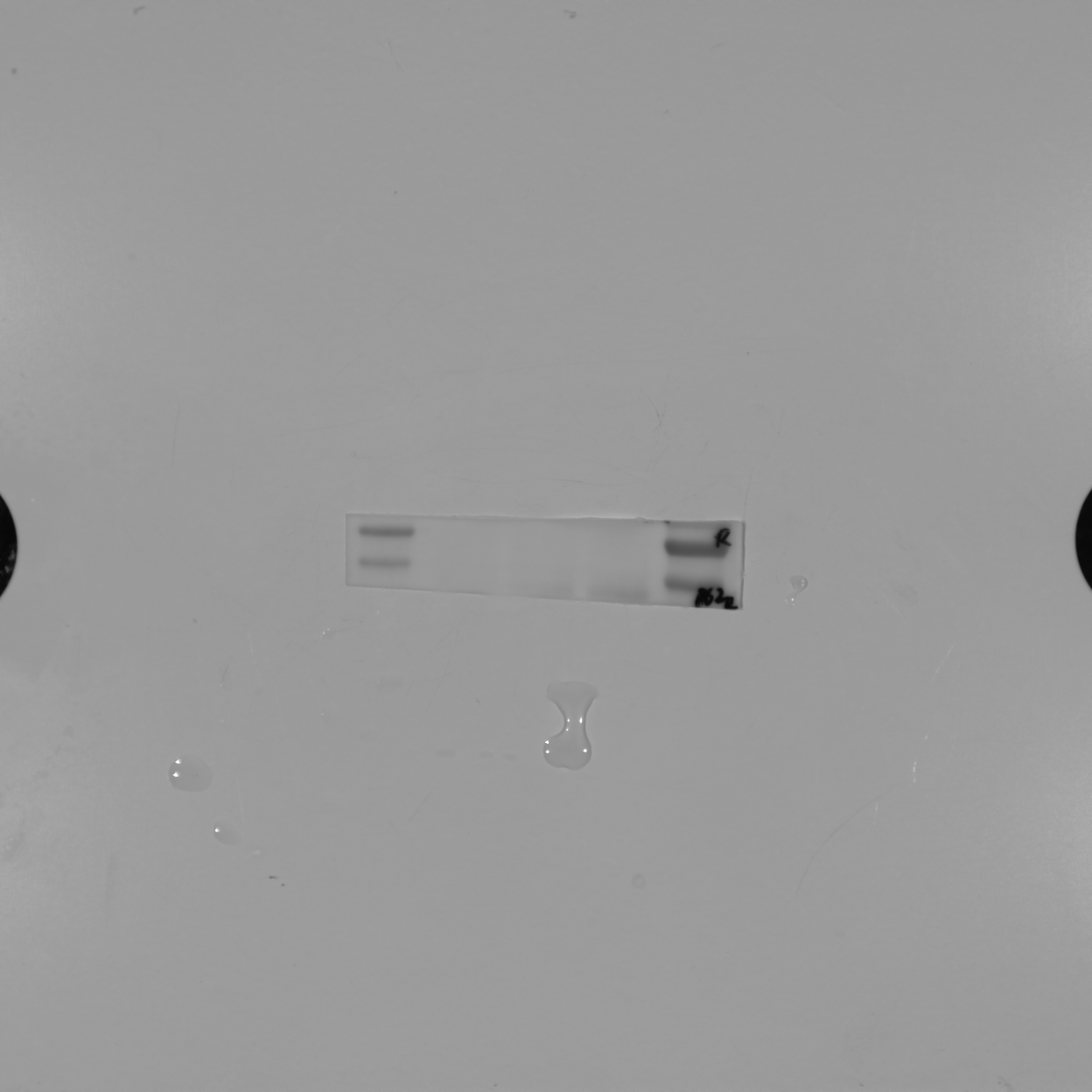

Supplement: Supplementary file 10 — Original WB [file 41420_2023_1579_MOESM10_ESM.zip › Original WB/Figure 6C/R-p62 20220323_180856_Ch已用/R-p62 20220323_180856_Ch_Marker.tif]

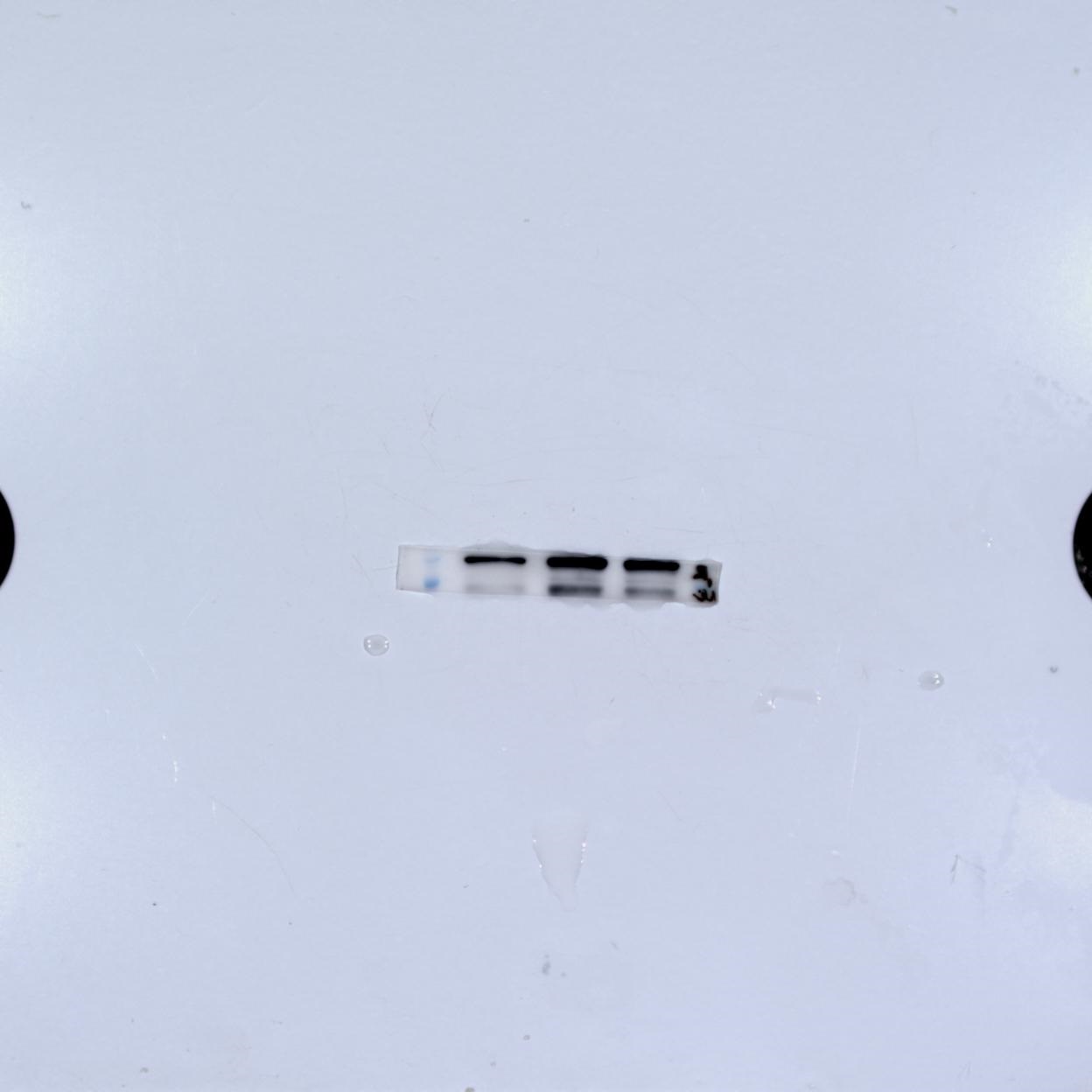

Supplement: Supplementary file 10 — Original WB [file 41420_2023_1579_MOESM10_ESM.zip › Original WB/Figure 7D/E-cad/R-Ecad 20220327_194846_Ch+Marker.jpg]

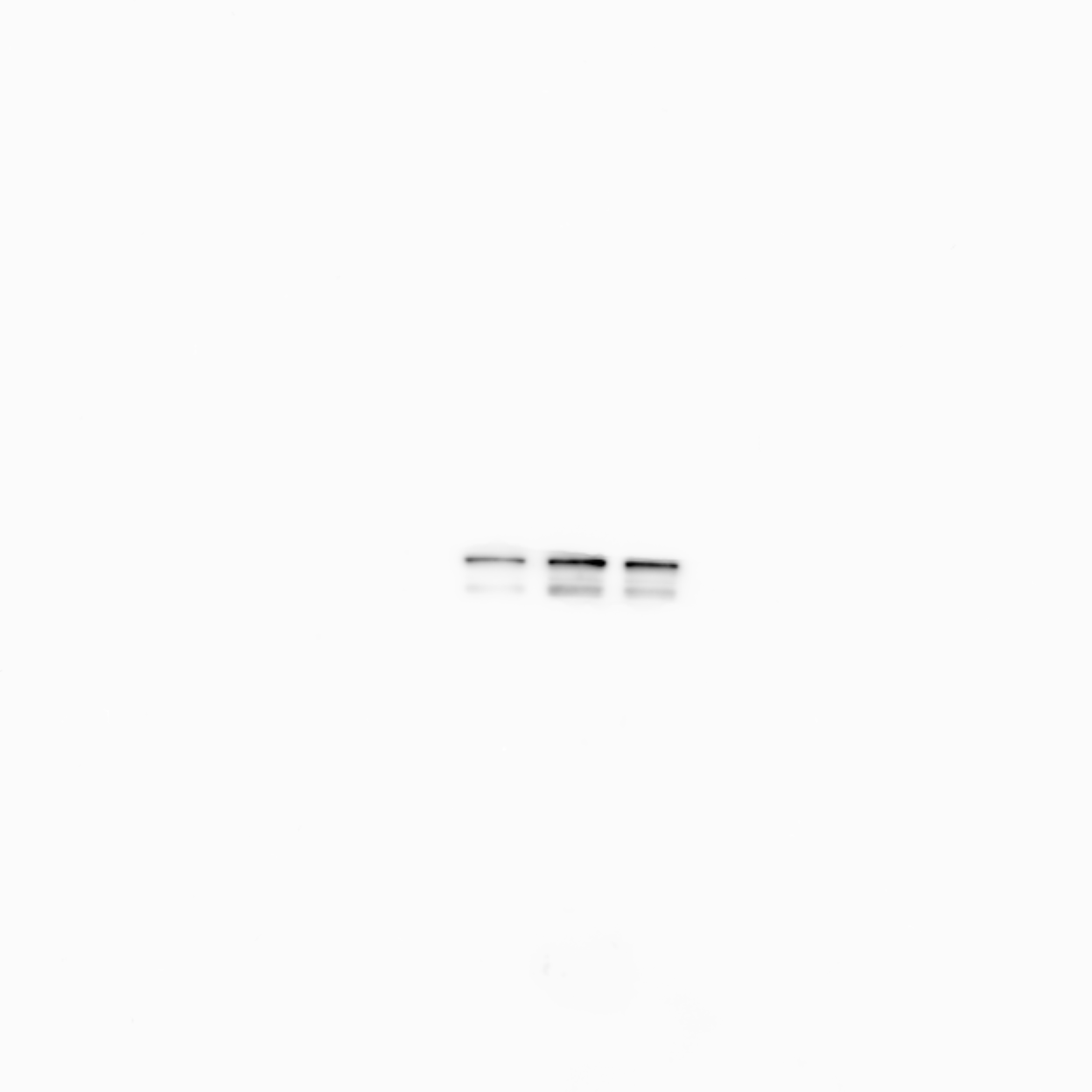

Supplement: Supplementary file 10 — Original WB [file 41420_2023_1579_MOESM10_ESM.zip › Original WB/Figure 7D/E-cad/R-Ecad 20220327_194846_Ch.tif]

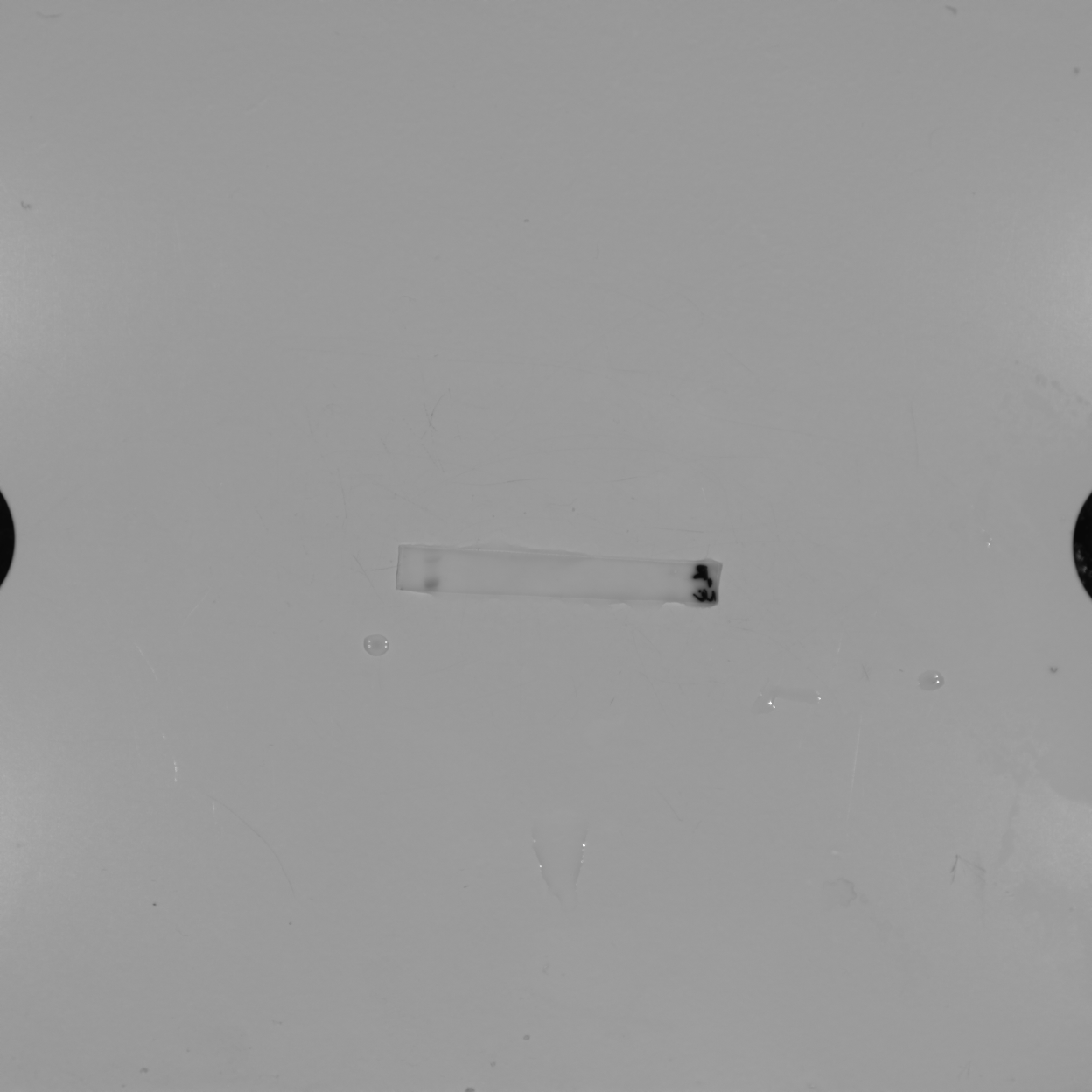

Supplement: Supplementary file 10 — Original WB [file 41420_2023_1579_MOESM10_ESM.zip › Original WB/Figure 7D/E-cad/R-Ecad 20220327_194846_Ch_Marker.tif]

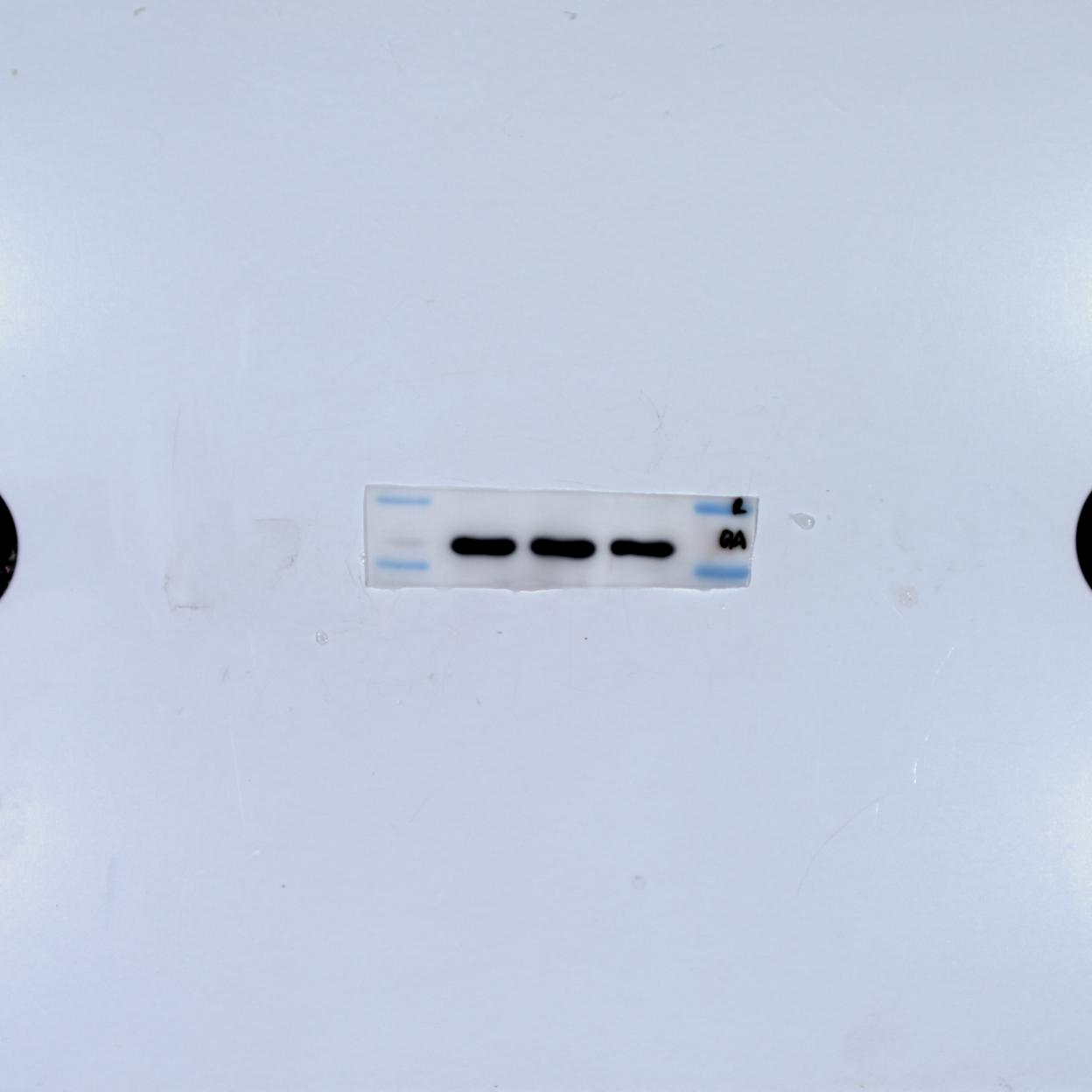

Supplement: Supplementary file 10 — Original WB [file 41420_2023_1579_MOESM10_ESM.zip › Original WB/Figure 7D/R-GA2 20220323_174716_Ch已用/R-GA2 20220323_174716_Ch+Marker.jpg]

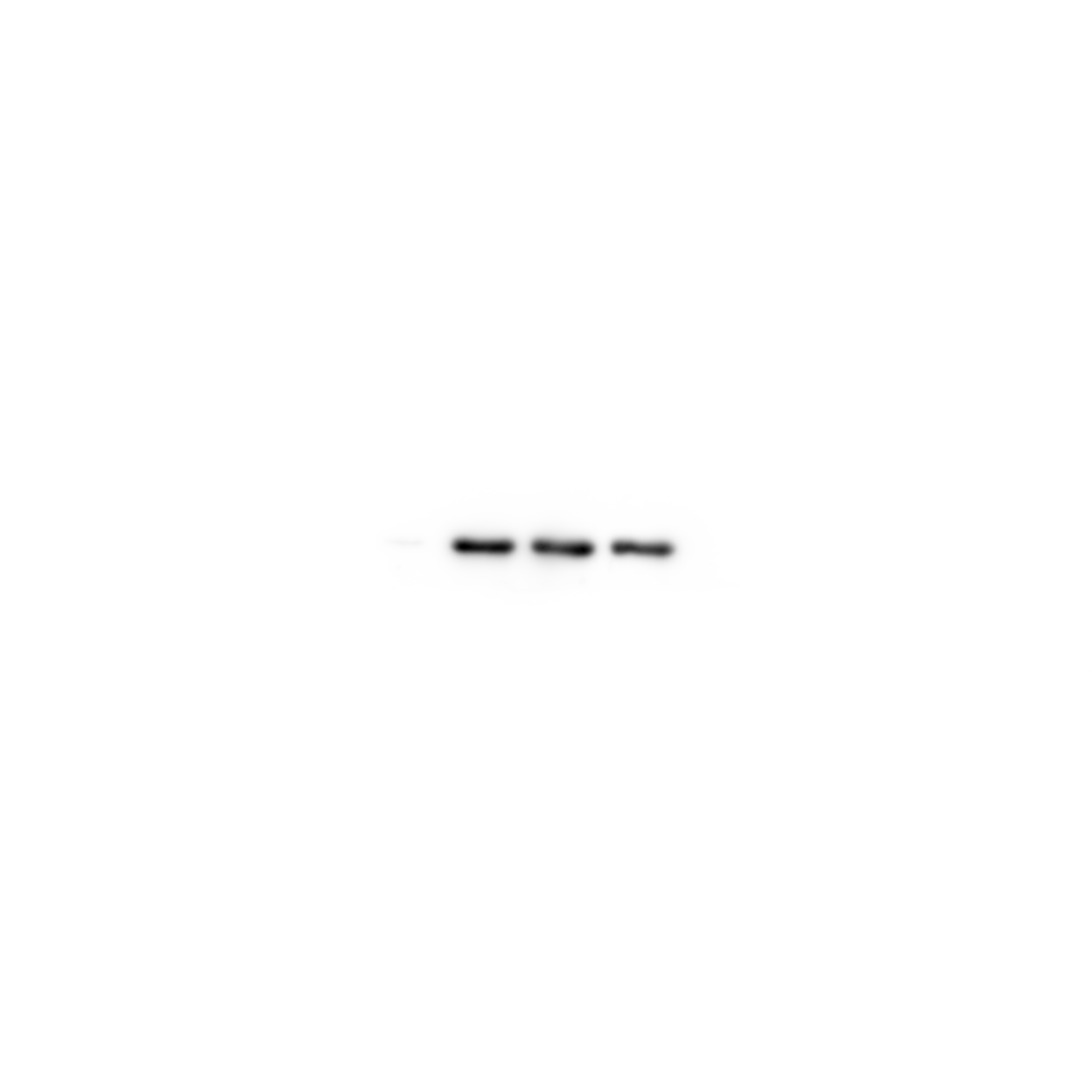

Supplement: Supplementary file 10 — Original WB [file 41420_2023_1579_MOESM10_ESM.zip › Original WB/Figure 7D/R-GA2 20220323_174716_Ch已用/R-GA2 20220323_174716_Ch.tif]

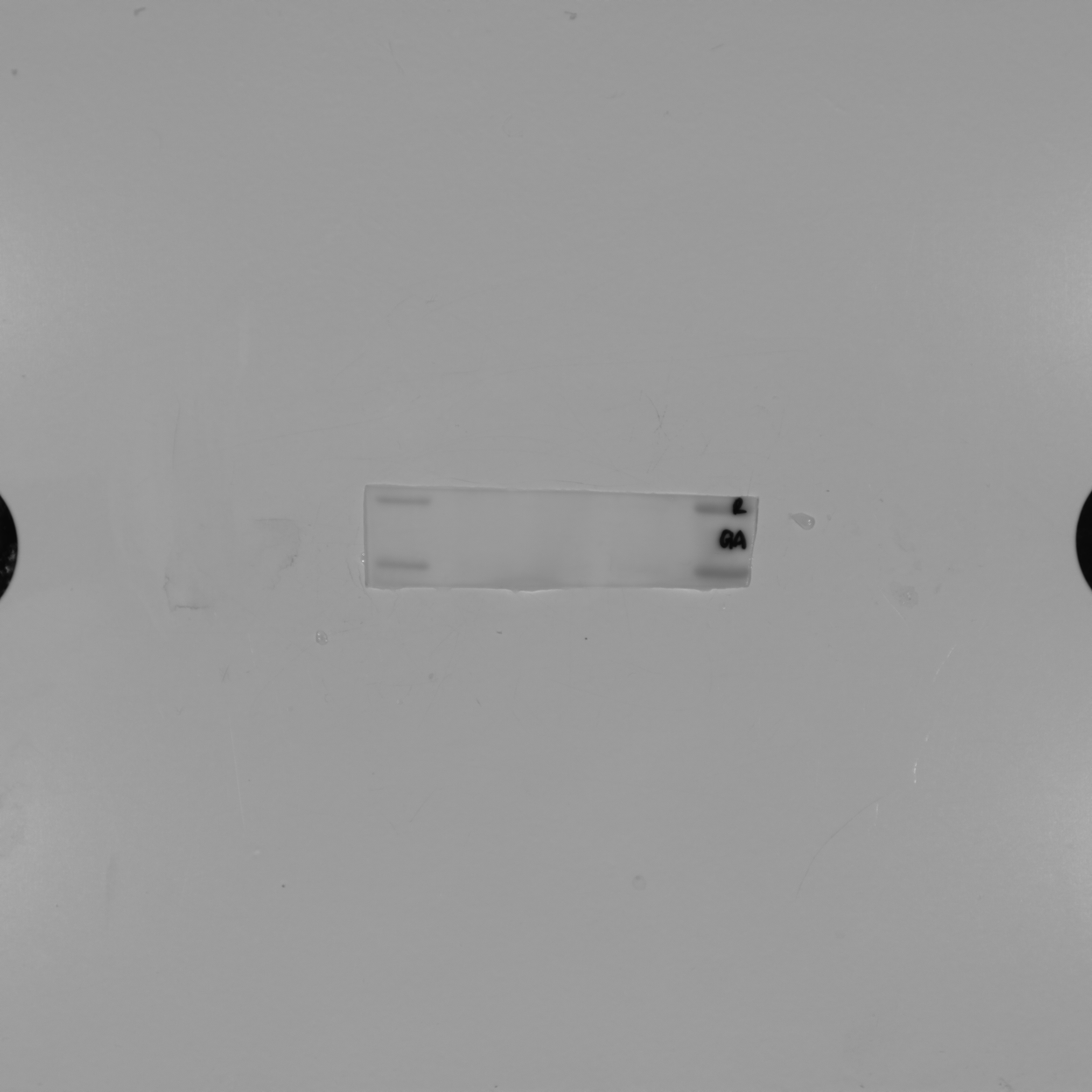

Supplement: Supplementary file 10 — Original WB [file 41420_2023_1579_MOESM10_ESM.zip › Original WB/Figure 7D/R-GA2 20220323_174716_Ch已用/R-GA2 20220323_174716_Ch_Marker.tif]

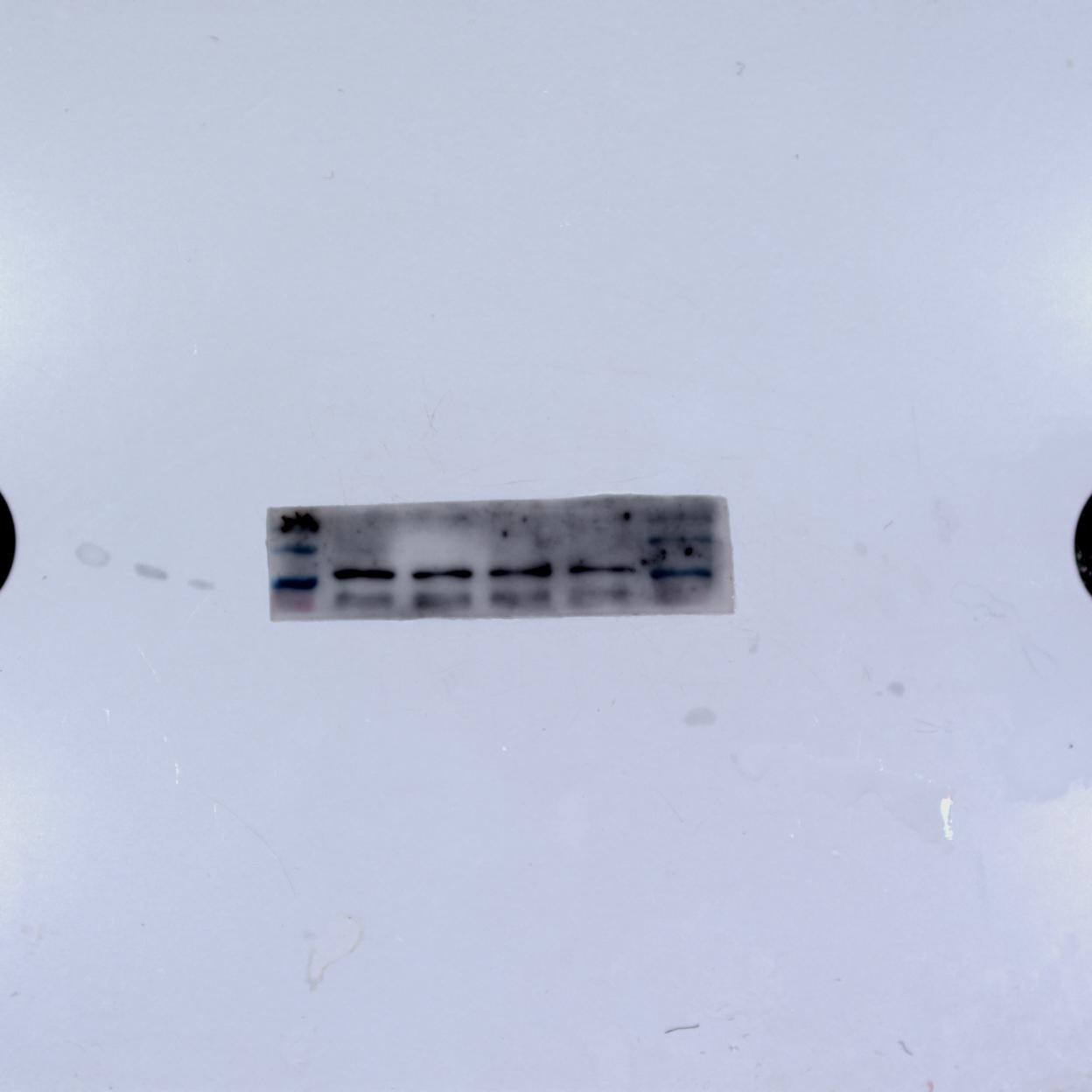

Supplement: Supplementary file 10 — Original WB [file 41420_2023_1579_MOESM10_ESM.zip › Original WB/Figure 7D/c-ncad2 20220409_205455_Ch已用/c-ncad2 20220409_205455_Ch+Marker.jpg]

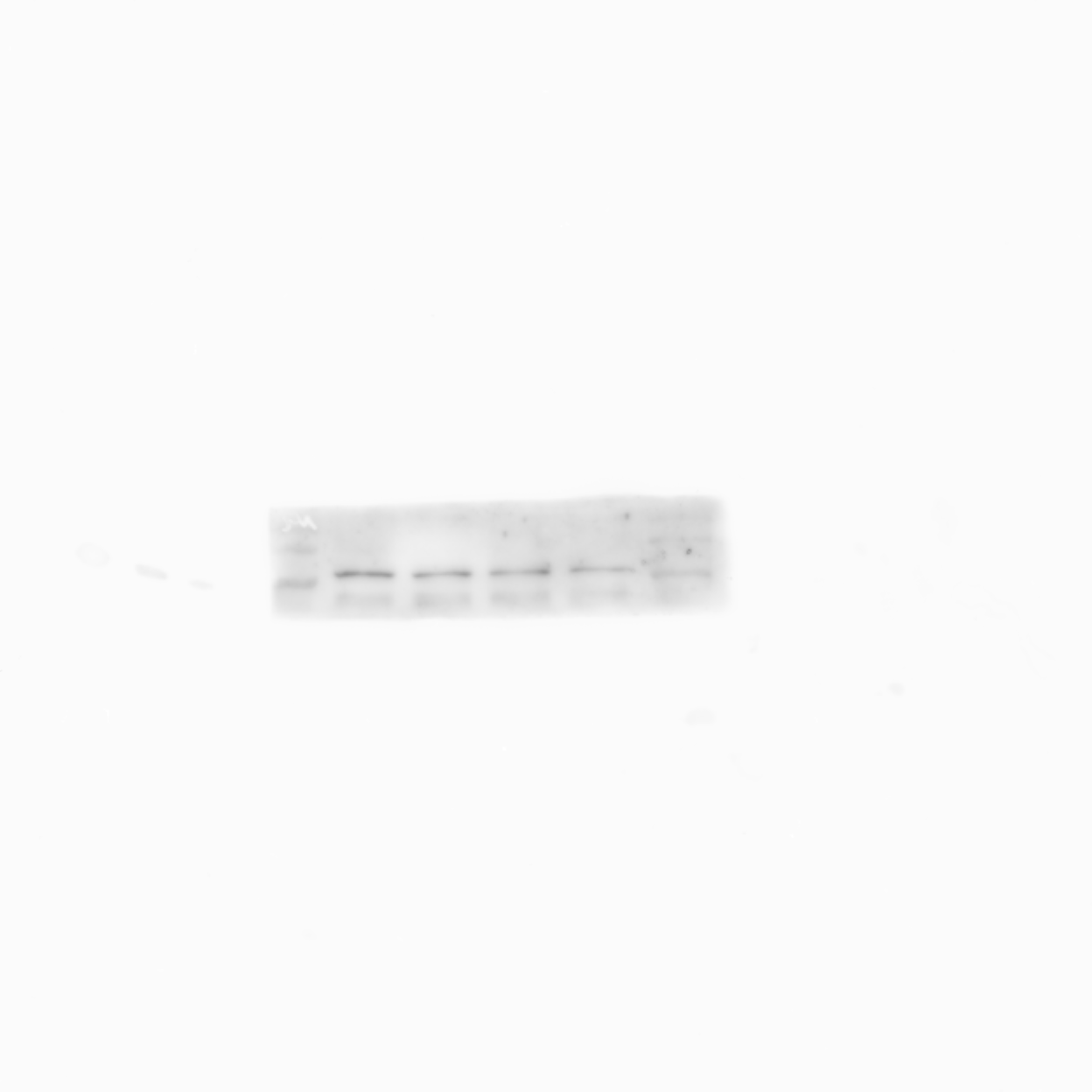

Supplement: Supplementary file 10 — Original WB [file 41420_2023_1579_MOESM10_ESM.zip › Original WB/Figure 7D/c-ncad2 20220409_205455_Ch已用/c-ncad2 20220409_205455_Ch.tif]

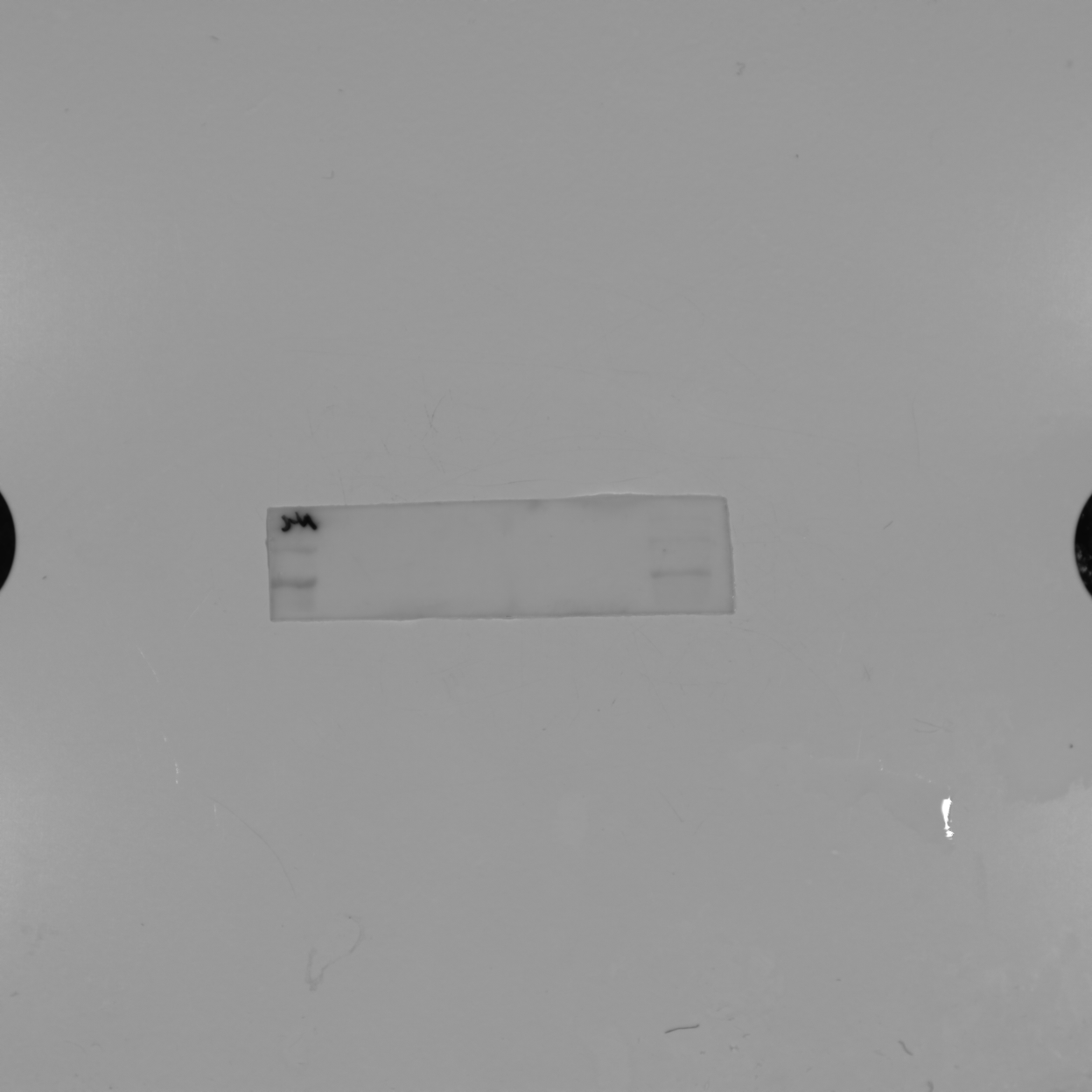

Supplement: Supplementary file 10 — Original WB [file 41420_2023_1579_MOESM10_ESM.zip › Original WB/Figure 7D/c-ncad2 20220409_205455_Ch已用/c-ncad2 20220409_205455_Ch_Marker.tif]

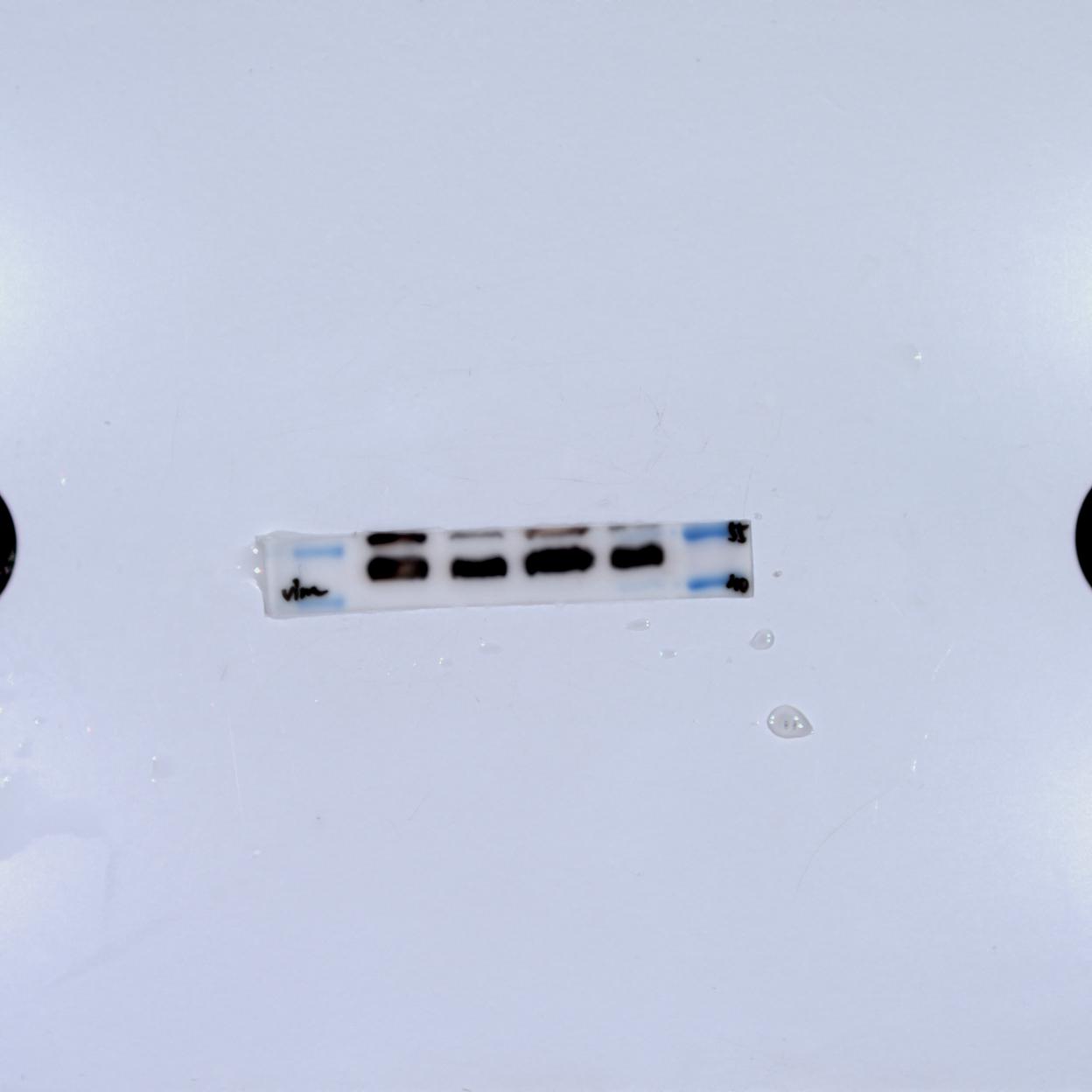

Supplement: Supplementary file 10 — Original WB [file 41420_2023_1579_MOESM10_ESM.zip › Original WB/Figure 7D/vim 20220317_220836_Ch已用/vim 20220317_220836_Ch+Marker.jpg]

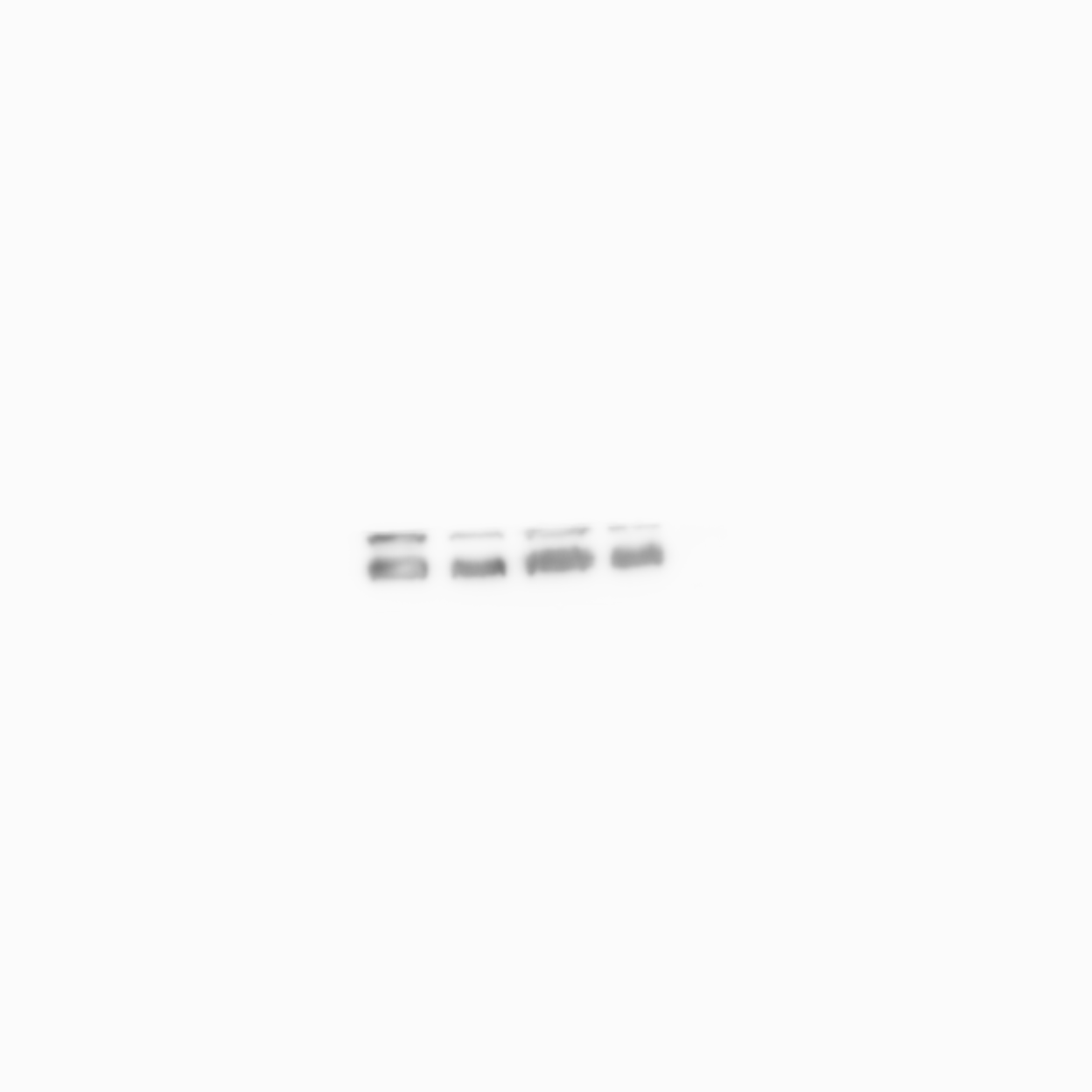

Supplement: Supplementary file 10 — Original WB [file 41420_2023_1579_MOESM10_ESM.zip › Original WB/Figure 7D/vim 20220317_220836_Ch已用/vim 20220317_220836_Ch.tif]

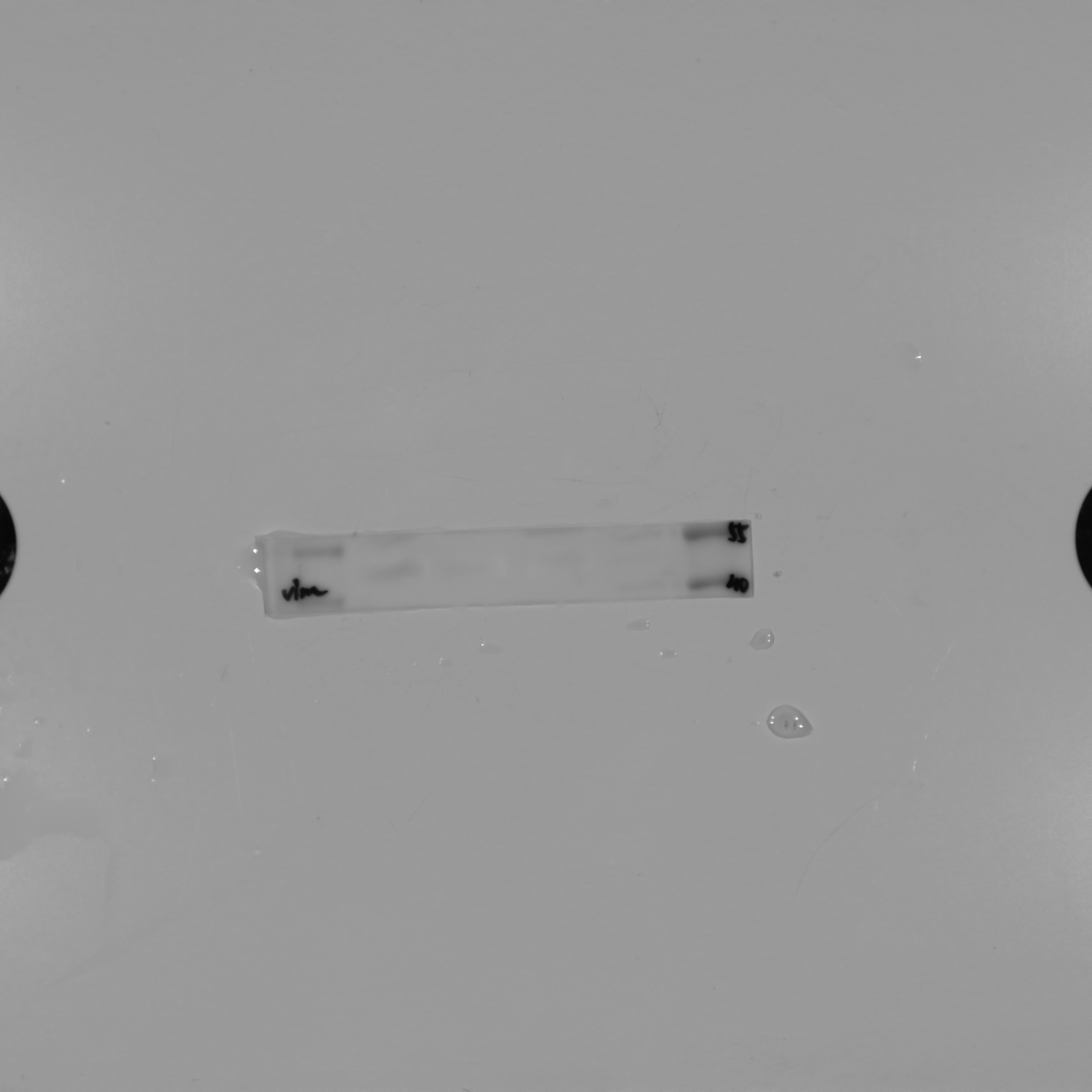

Supplement: Supplementary file 10 — Original WB [file 41420_2023_1579_MOESM10_ESM.zip › Original WB/Figure 7D/vim 20220317_220836_Ch已用/vim 20220317_220836_Ch_Marker.tif]

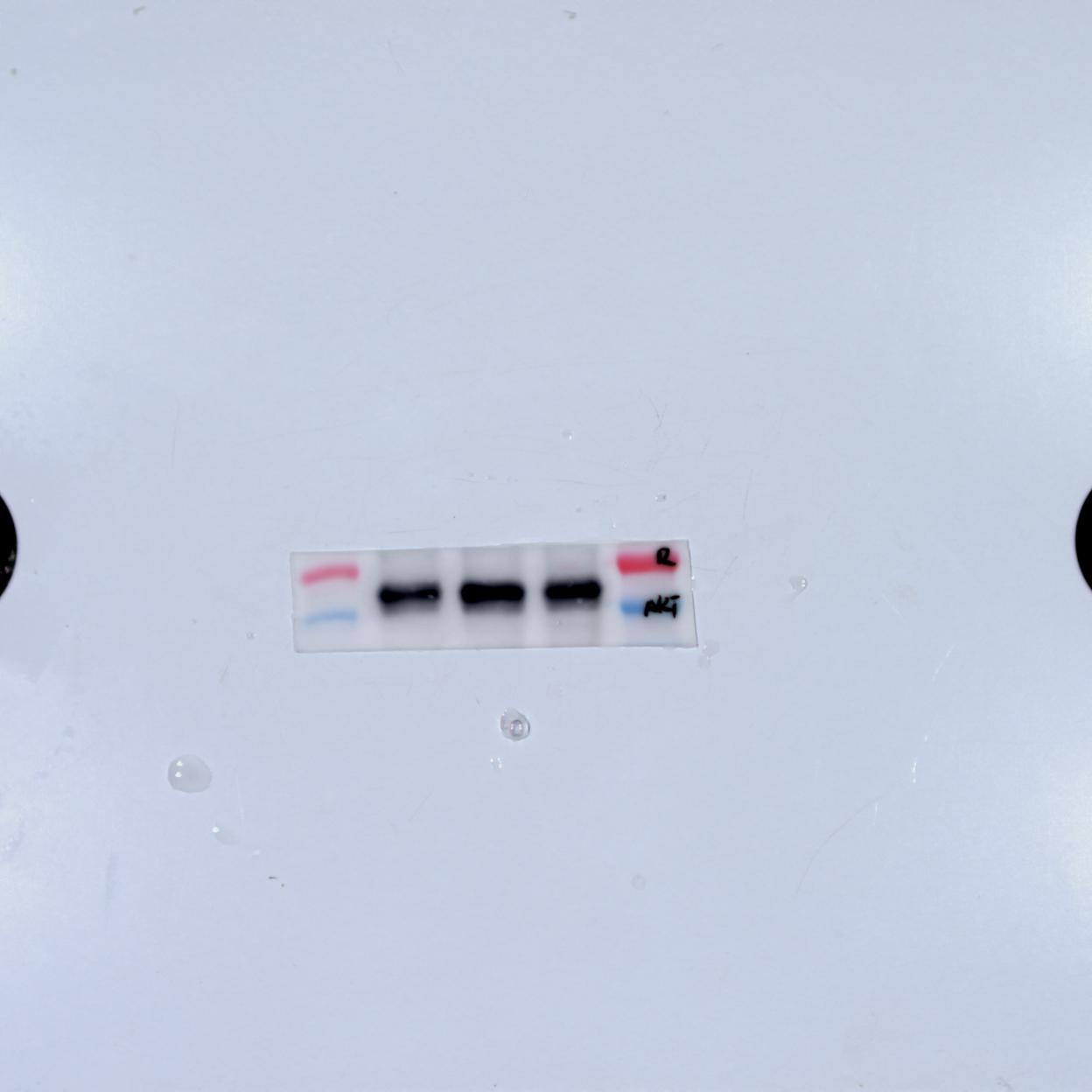

Supplement: Supplementary file 10 — Original WB [file 41420_2023_1579_MOESM10_ESM.zip › Original WB/Figure 8B/R-AKT 20220323_181105_Ch已用/R-AKT 20220323_181105_Ch+Marker.jpg]

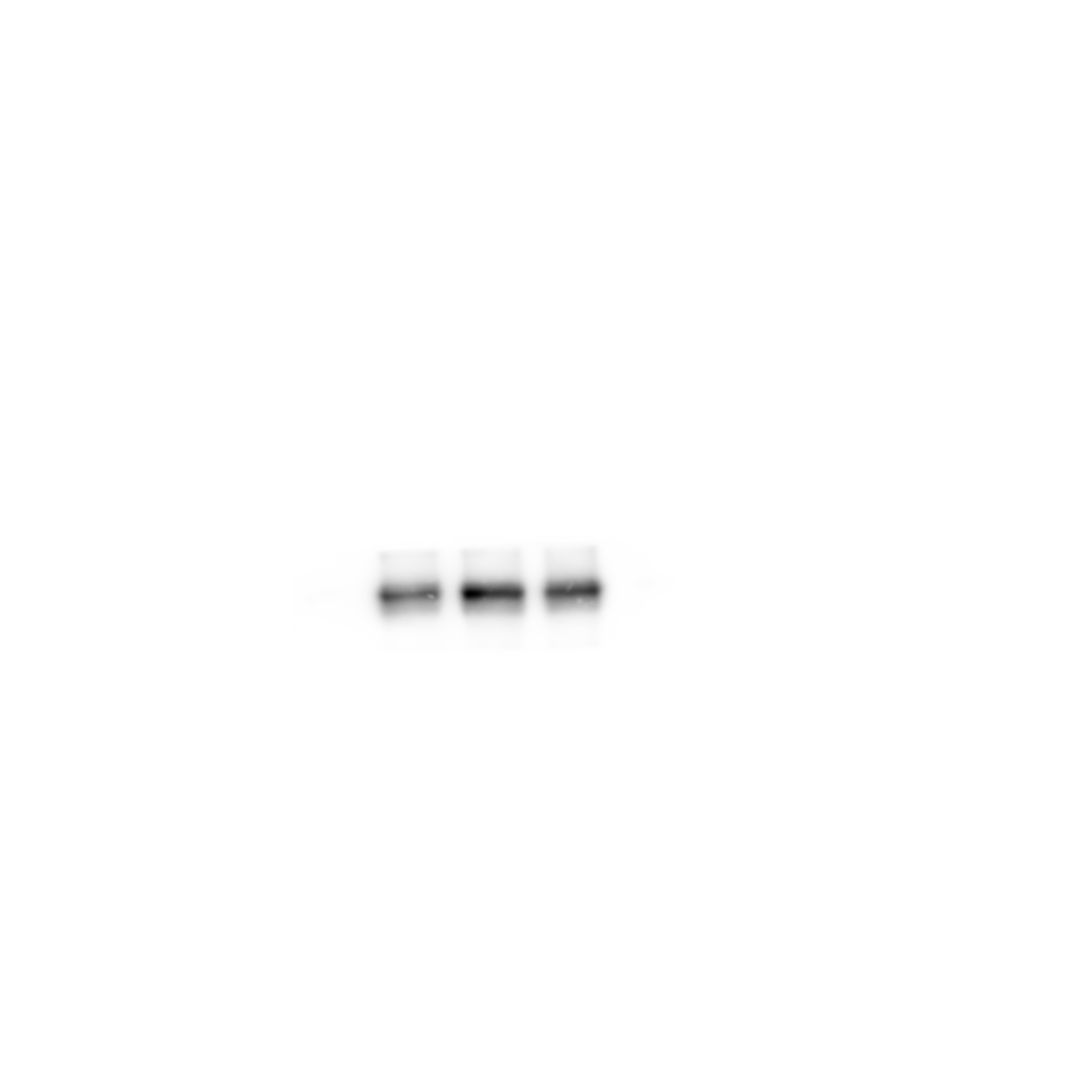

Supplement: Supplementary file 10 — Original WB [file 41420_2023_1579_MOESM10_ESM.zip › Original WB/Figure 8B/R-AKT 20220323_181105_Ch已用/R-AKT 20220323_181105_Ch.tif]

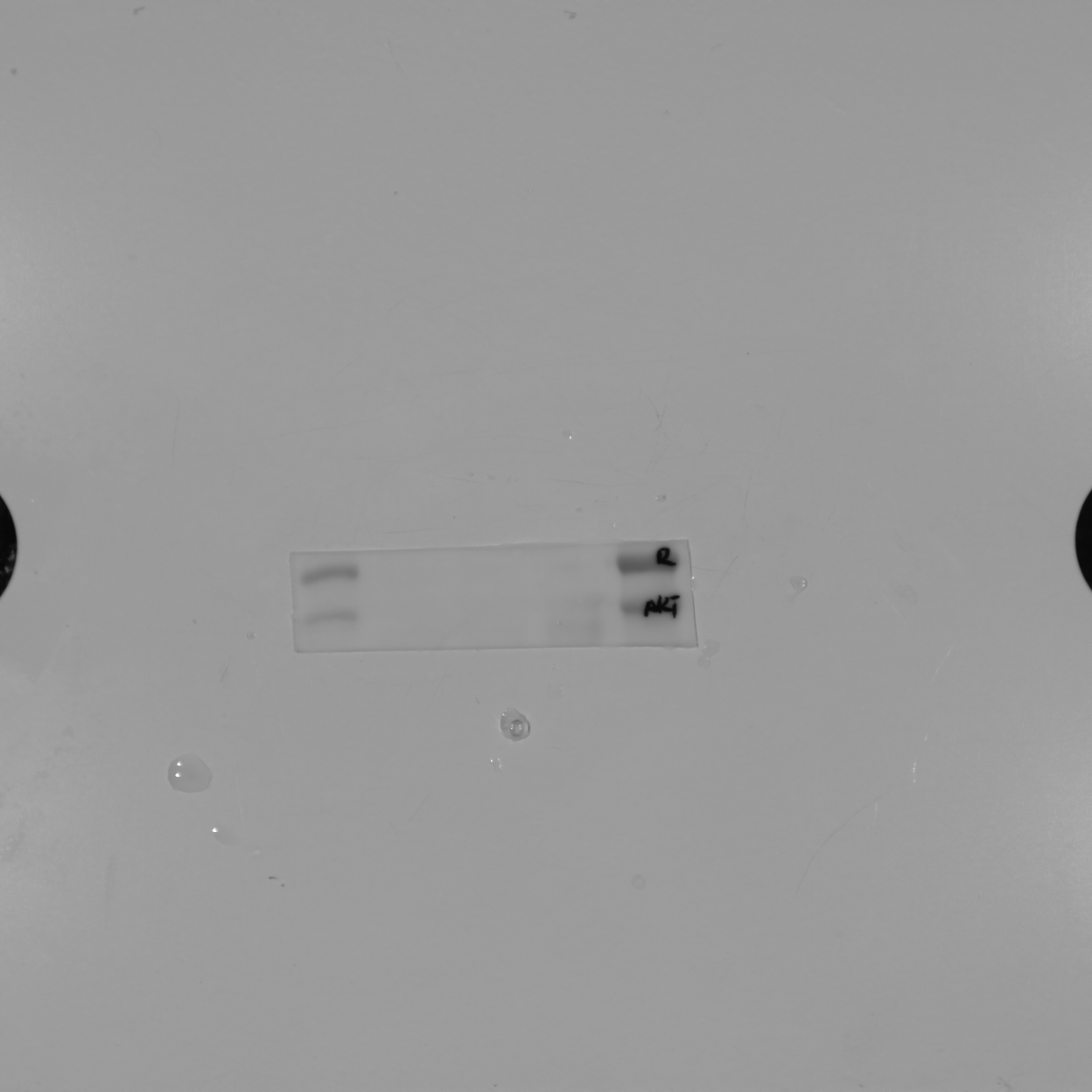

Supplement: Supplementary file 10 — Original WB [file 41420_2023_1579_MOESM10_ESM.zip › Original WB/Figure 8B/R-AKT 20220323_181105_Ch已用/R-AKT 20220323_181105_Ch_Marker.tif]

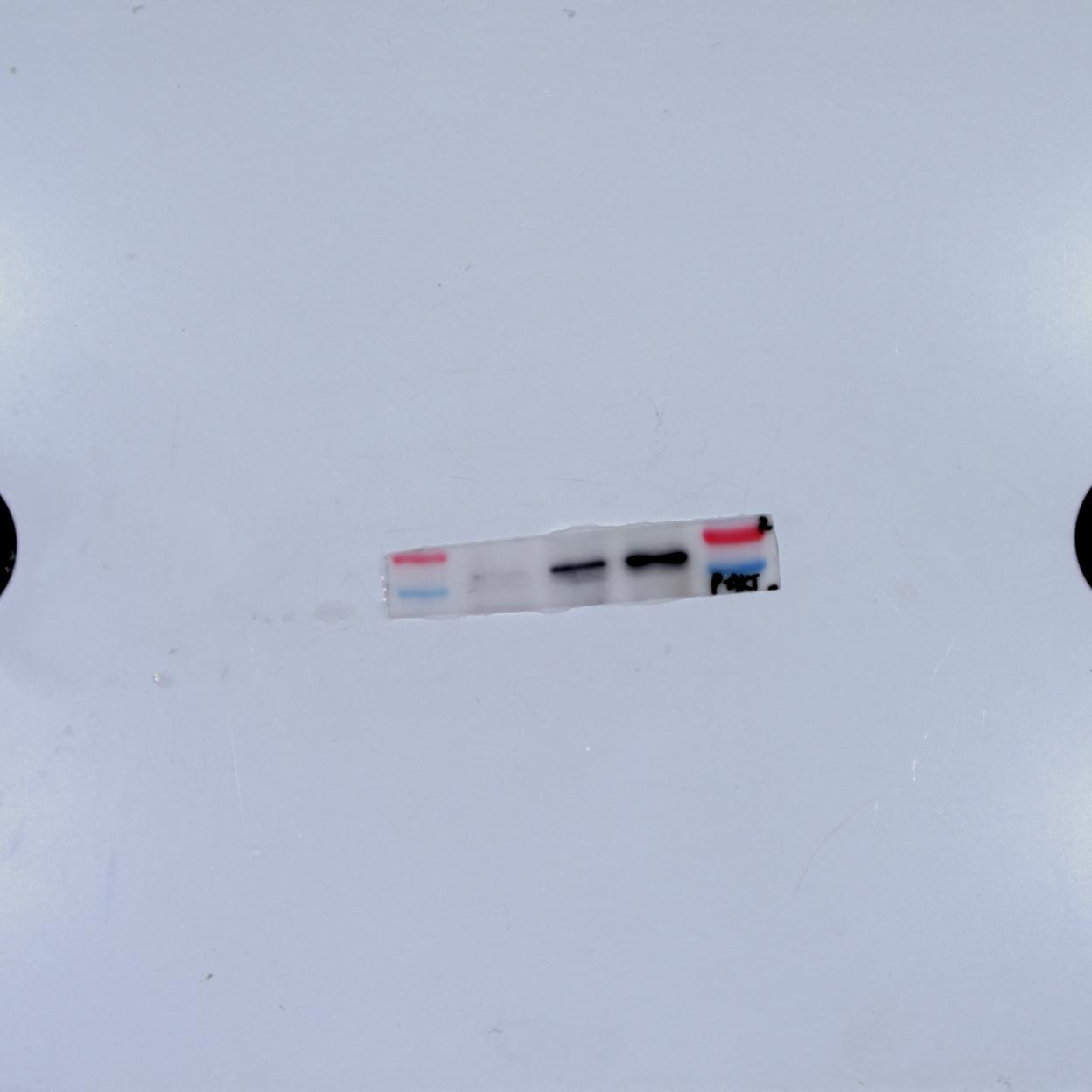

Supplement: Supplementary file 10 — Original WB [file 41420_2023_1579_MOESM10_ESM.zip › Original WB/Figure 8B/R-pAKT 20220323_181902_Ch已用/R-pAKT 20220323_181902_Ch+Marker.jpg]

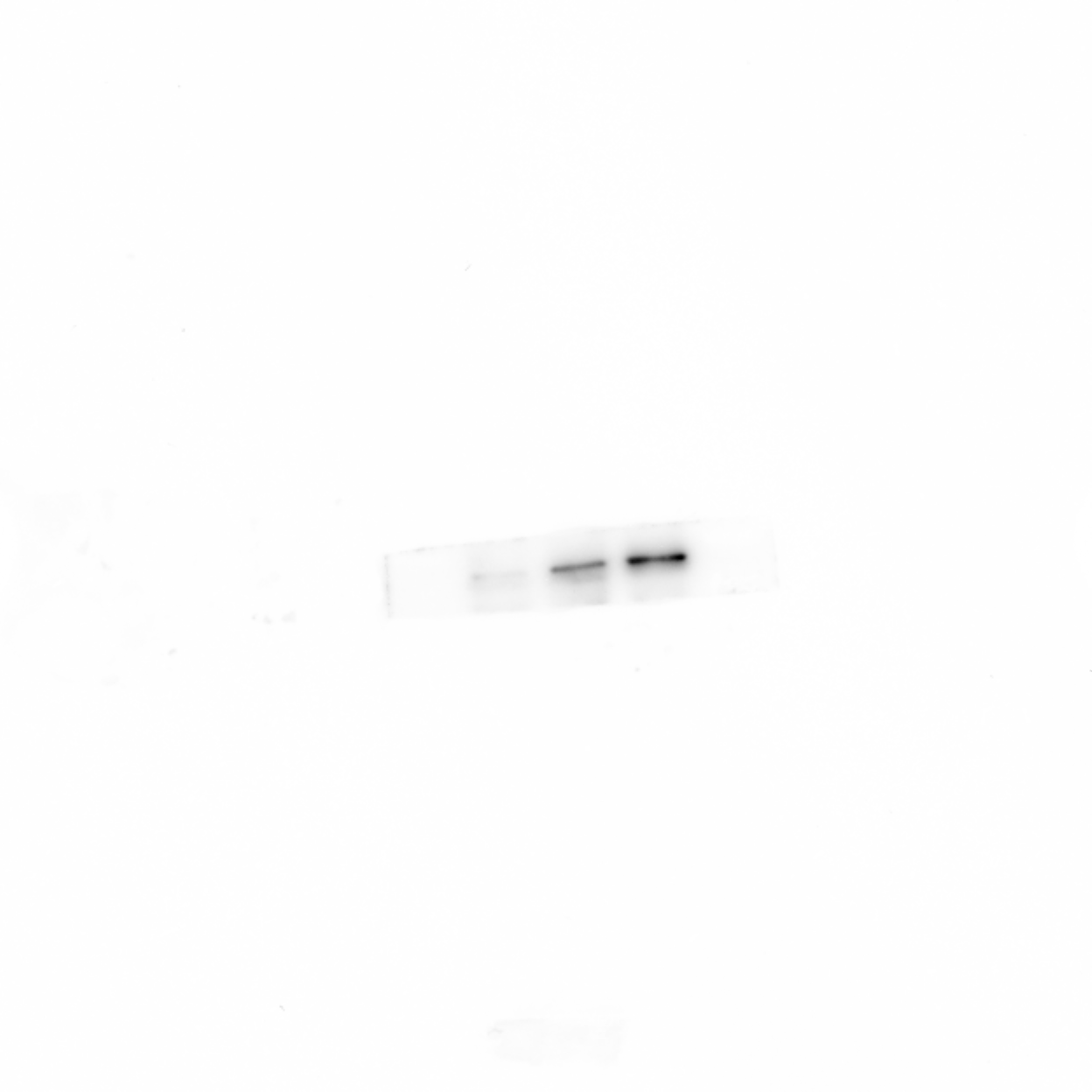

Supplement: Supplementary file 10 — Original WB [file 41420_2023_1579_MOESM10_ESM.zip › Original WB/Figure 8B/R-pAKT 20220323_181902_Ch已用/R-pAKT 20220323_181902_Ch.tif]

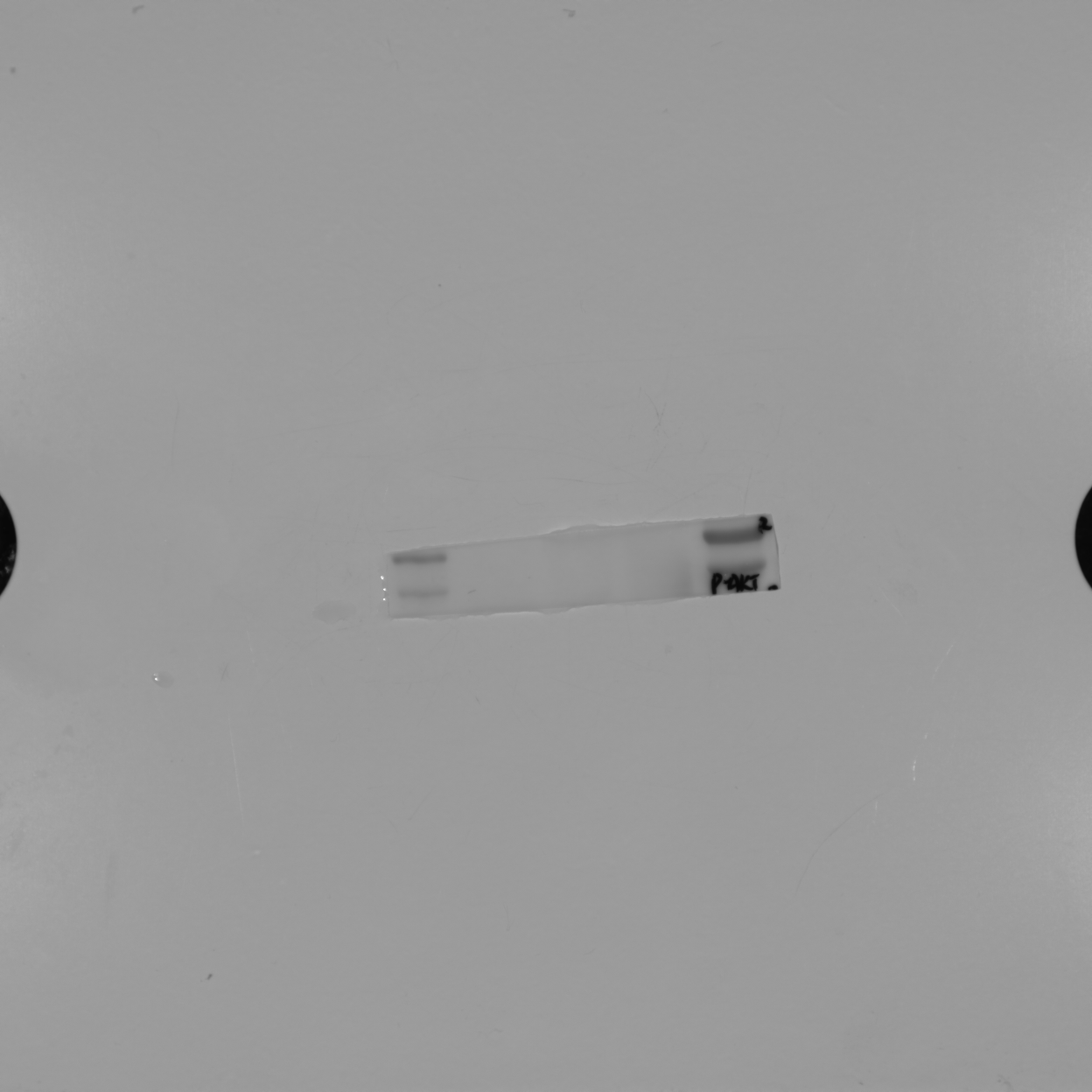

Supplement: Supplementary file 10 — Original WB [file 41420_2023_1579_MOESM10_ESM.zip › Original WB/Figure 8B/R-pAKT 20220323_181902_Ch已用/R-pAKT 20220323_181902_Ch_Marker.tif]
